# A synthetic rainbow trout linkage map provides new insights into the salmonid whole genome duplication and the conservation of synteny among teleosts

**DOI:** 10.1186/1471-2156-13-15

**Published:** 2012-03-16

**Authors:** René Guyomard, Mekki Boussaha, Francine Krieg, Caroline Hervet, Edwige Quillet

**Affiliations:** 1 INRA, UMR1313, Animal Genetics and Integrative Biology, Domaine de Vilvert, 78350 Jouy-en-Josas, France

## Abstract

**Background:**

Rainbow trout is an economically important fish and a suitable experimental organism in many fields of biology including genome evolution, owing to the occurrence of a salmonid specific whole-genome duplication (4^th ^WGD). Rainbow trout is among some of the most studied teleosts and has benefited from substantial efforts to develop genomic resources (e.g., linkage maps. Here, we first generated a synthetic map by merging segregation data files derived from three independent linkage maps. Then, we used it to evaluate genome conservation between rainbow trout and three teleost models, medaka, stickleback and zebrafish and to further investigate the extent of the 4^th ^WGD in trout genome.

**Results:**

The INRA linkage map was updated by adding 211 new markers. After standardization of marker names, consistency of marker assignment to linkage groups and marker orders was checked across the three different data sets and only loci showing consistent location over all or almost all of the data sets were kept. This resulted in a synthetic map consisting of 2226 markers and 29 linkage groups spanning over 3600 cM. Blastn searches against medaka, stickleback, and zebrafish genomic databases resulted in 778, 824 and 730 significant hits respectively while blastx searches yielded 505, 513 and 510 significant hits. Homology search results revealed that, for most rainbow trout chromosomes, large syntenic regions encompassing nearly whole chromosome arms have been conserved between rainbow trout and its closest models, medaka and stickleback. Large conserved syntenies were also found between the genomes of rainbow trout and the reconstructed teleost ancestor. These syntenies consolidated the known homeologous affinities between rainbow trout chromosomes due to the 4^th ^WGD and suggested new ones.

**Conclusions:**

The synthetic map constructed herein further highlights the stability of the teleost genome over long evolutionary time scales. This map can be easily extended by incorporating new data sets and should help future rainbow trout whole genome sequence assembly. Finally, the persistence of large conserved syntenies across teleosts should facilitate the identification of candidate genes through comparative mapping, even if the occurrence of intra-chromosomal micro-rearrangement may hinder the accurate prediction their genomic location.

## Background

Whole genome sequences and dense type II marker linkage maps are available in a fast-growing number of organisms. They have been extensively compared to trace the evolution of genomes in the major vertebrate phyla across large time-scales and to recover the events and mechanisms which could have occurred, such as the number and nature of rearrangements within and between chromosomes and the extent and fate of gene or genome duplications [1-5]. Several major evolutionary issues have emerged from these comparative studies, particularly the validation of the hypothesis that two whole genome duplications occurred in the early vertebrate lineage evolution before the split between ray-fined fishes and land tetrapodes (the 1^st ^WGD before the agnatha-gnatostoma split, the 2^nd ^WGD before the chrondrichthyes-osteichthyes split) and a third one specific to the actinopterygians [6-8]. Additional whole genome duplications have occurred in some fish families such as Acipenseridae, Catostomidae, Cobitidae, Cyprinidae and Salmonidae [9]. A second important outcome has been the reconstitution of the ancestral vertebrate proto-chromosomes and their fate along the different vertebrate lineages, based on the distribution of conserved syntenic blocks among vertebrates. This has facilitated tracing the origins of the proto-vertebrate karyotype back to 10 ancestral chromosomes prior to their radiation in the chordates [10-12]. Such reconstitutions have led to the conclusion that chromosomes have been reshaped through inversions within chromosomes rather than translocations in teleosts while inter-chromosomal rearrangements have been very frequent in the tetrapod lineage [11,12].

Another outcome of comparative genomics is the identification of of candidate genes. Once identified further studies can elucidate the function and/or regulation of these genes with respect to their influence on physiological or morphological traits. In this approach, the identification of the master gene in a given genomic region of the target species is expected to result from the discovery of a strong putative candidate gene in a homologous conserved region of the genome of model organisms. The success of this approach depends on the extent of synteny and linkage conservation between target and model species. In mammals, a high degree of conservation of synteny and, to a less extent, of linkage has been found between species. In this regard, 51 conserved syntenic groups and 173 conserved linkage segments have been identified between pig and human [13], two species which have diverged from their common ancestor 80 million years ago. Comparative approaches have been successful in localizing causal mutations to genes inferred through candidate positional cloning rather than strict positional cloning [14].

Rainbow trout (*Oncorhynchus mykiss*) is one of the most-widely cultivated cold freshwater fish in the world. Its worldwide distribution is mainly due to its great potential for aquaculture production and sport fisheries. In addition to its commercial interest, rainbow trout is a well suited organism for academic studies in the fields of carcinogenesis, toxicology, comparative immunology, disease, ecology, physiology, transgenesis, nutrition and evolutionary genetics [15]. Among salmonid species, accumulating evidence support the hypothesis that a specific whole genome duplication (4^th ^WGD) event occurred 50 to 100 million years ago in the family ancestor [16]. This relatively recent event has made the rainbow trout an interesting model to study evolution of duplicated genes after WGD.

Great efforts have been and are still devoted to the development of genomic tools in rainbow trout. These resources include large expressed sequence tags (EST) [17,18] and BAC end sequence (BES) [19] databases, fingerprinted BAC libraries [20], type I and type II linkage maps [21-23]. More recently, an integrated physical map anchoring BAC contigs on the current linkage maps has been produced [24] and the whole genome sequencing is currently in progress [25,26].

Three linkage maps each consisting of approximately one thousand markers and covering all chromosome arms have been previously constructed independently [21-23]. They provided a more accurate picture of the distribution and organization of the duplicated regions of the rainbow trout genome. They have also facilitated investigations into the extent of conserved syntenies among various model fish species and rainbow trout, which aid in the reconstruction of proto-chromosomes in the salmonid genome [22,23,27].

In this study, we updated the INRA rainbow trout linkage map by including more than two hundred new SNP and microsatellite markers. We then produced a synthetic map of more than two thousand markers by integrating the three most informative rainbow trout linkage maps (ARS [23], UoG (University of Guelph) [22] and INRA [21]). A high degree of consistency between the three linkage maps was observed. The extended information from the synthetic map was then used to further investigate the putative number and extent of duplicated regions in rainbow trout genome and to re-assess the extent of synteny and linkage conservation between rainbow trout and three model species, namely medaka (*Oriza latipes*), stickleback (*Gasterosteus aculeatus*) and zebrafish (*Danio rerio*).

## Results

### Update of the INRA linkage map

A new INRA linkage map was constructed by adding 211 new loci (177 SNPs and 81 microsatellites) to the previously published one [21]. These were listed in Additional file 1, sheet 10. The new map consisted of 1109 markers, out of which, 266 were duplicated (133 pairs of loci). Linkage group assignments or positions in the linkage group have been corrected for a number of markers after comparison with other published maps (see next section). Linkage groups RT04 and RT25 were unlinked in the current INRA map, but were artificially merged to form a metacentric linkage group, as previously reported by Danzmann *et al. *[22] and Rexroad *et al. *[23]. The updated INRA linkage map thus consisted of 29 linkage groups and spanned a total length of 2900 cM (Additional file 1, sheet 2; Additional file 2, sheet 3 and Additional file 3).

### Nomenclature standardization and consistency of data sets

Data sets were filtered to remove problematic markers using the following filtration steps. First, linkage groups which were merged in the UoG data sets (*i.e*. RT-5 and 31f; see Additional file 3 in Danzmann *et al. *, [22]) were excluded from the analysis. This led to elimination of 16 markers from the original UoG data sets. Second, standardization of locus names led to the identification and elimination of 16 redundant loci being used under two different names in the same map (Additional file 2, sheet 2). Third, linkage group assignments were not consistent for 6 markers (OMM1034, Omi84TUF, OmyFGT32TUF, OmyRGT42TUF, OmyUW1552 and Ssa407UoS) across the different data sets; these were indicated by a suffix indicating the source data set. Fourth, analyses of consistencies of marker orders within each linkage group across all data sets revealed 133 cases of discrepancies. Out of these, 77 markers involved a single data set *versus *several other ones (Additional file 1; sheet 8). These markers were removed from the deviant data set. The remaining 56 cases involved markers for which segregation data were available in two mapping parents only (Additional file 1; sheet 9). In 35 cases, these discrepancies resulted from one or two inconsistent genotypes in one of the two sets and those inconsistencies were therefore ignored; the remaining 21 loci corresponded to more important differences. A suffix was added to these markers in order to identify the source data set.

### Construction of the synthetic map

**Figure 1:**
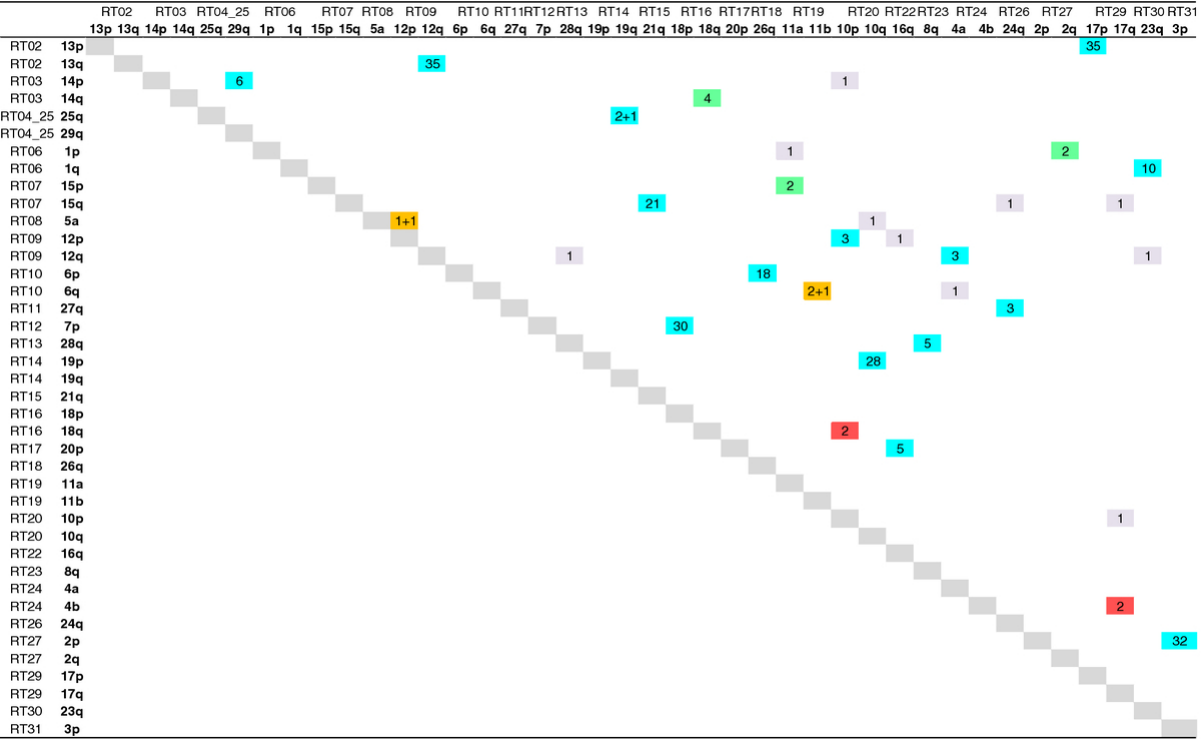
**Oxford grid showing the homeologous relationships which have been conserved between rainbow trout chromosome arms after the 4^th ^Whole Genome Duplication specific to salmonids**. Linkage group and chromosome arm numbers are indicated on first and second lines and rows respectively. Numbers of markers shared between duplicated regions are given. When two numbers per cell are given (*i.e*. 1 + 1), the second one corresponds to additional homeologies found by comparing the rainbow trout and model species linkage groups. Blue cells: previously well-characterised homeologies. Green and orange cells: homologies respectively based on a single marker or suggested in Danzmann *et al. *[22] and confirmed with additional duplicated markers in the current study. Red cells: newly identified homeologies supported by two or more markers. Grey cells: homeologies based on a single marker and not considered in this study.

The final synthetic map consisted of 2226 loci assigned to 29 linkage groups and spanned a total length of approximately 3600 cM (Additional file 2, sheet 4 and Additional file 4). Marker orders were identical to those of the INRA map in 20 linkage groups (RT03 to RT06, RT08 to RT12, RT14, RT15, RT17 to RT19, RT22, RT24 to RT30) (Additional file 2, sheets 6 to 34). The remaining nine linkage groups showed very minor inversions between some consecutive loci (Additional file 2, sheets 6 to 34). Approximate borders of centromeric regions were defined as described in [21] and included all the markers mapped within these borders. Two hundred and eighty-two pairs of duplicated loci (576 loci, *i.e*. 26% of the total number of loci) were found on all chromosomal arms, with the exception of Omy22p, Omy7q, Omy21p and Omy20q (Additional file 2, sheet 35). Numbers of shared duplicated loci between homeologous arms ranged from one to 35 between Omy12q and Omy13q and 21 pairs of arms had two or more duplicated loci in common (Figure 1 and Additional file 2, sheet 35).

### Homology with other fish genomes and identification of syntenic regions

The rainbow trout sequence homology searches were conducted using both blastn and blastx alignment tools. Homology search results were summarized in Additional file 5 (sheets 4 to 9) which also contains blastn and blastx search results previously reported in Danzmann *et al. *[22] and Rexroad *et al. *[23] (Additional file 5, sheets 2 and 3). Blastn searches resulted in 778, 824, and 730 sequence reads having significant hits to the medaka, stickleback, and zebrafish genomes, respectively. For blastx analysis, we identified 505, 512, and 510 sequence reads having significant gene hits to the medaka, stickleback, and zebrafish genomes, respectively. Comparison of homology search results between the 3 studies [22,23], this study), revealed very few differences (Additional file 6; sheets 2 and 9). Identification of syntenic regions was primarily conducted using blastn and blastx results of the present study. When ambiguities were observed between blastn and blastx results or between the 3 different studies [22,23] and herein), preferment was given to the homology which was consistent with the pattern of conserved synteny identified with other markers in the same region of the rainbow trout linkage group. Details on marker assignments to linkage groups in rainbow trout and each of the three model species are summarized in Additional file 6 (sheets 2, 5 and 9).

### Correspondence between rainbow trout and model species linkage groups

Oxford grids were generated between rainbow trout chromosome arms and linkage groups of each model species (Additional file 6, sheets 4, 8 and 12). A total of 60, 50 and 89 homologies between a linkage group of a model species and a linkage group of rainbow trout were "orphan" homologies supported by a single marker in medaka, stickleback and zebrafish respectively. None of the orphan homologies found in comparison between medaka (alternately stickleback) and rainbow trout could be confirmed by an equivalent homology with another sequence in a comparison between stickleback (alternately medaka) and rainbow trout (this was not checked with zebrafish owing to the more complex pattern of conserved syntenies found with this species). Therefore, these orphan homologies were considered to be meaningless and were subsequently ignored.

In rainbow trout metacentric chromosomes, when both chromosome arms displayed homology with the same chromosome of a model species and if homology on one arm was supported by one marker only, then we assumed that this marker was assigned to the wrong arm and to a wrong position with respect to the centromere. Therefore, the marker was re-assigned to its putatively correct arm in the Oxford grids (11 cases in medaka and 15 in stickleback; Additional file 6, sheets 2 and 5).

Under these assumptions, rainbow trout chromosome arms displayed homology with a number of zebrafish chromosomes ranging from one to six (two to nine for chromosomes) and the total number over the whole rainbow trout genome was 126 (Table 1). For medaka and stickleback, the corresponding numbers were substantially lower: one to four chromosomes per rainbow trout chromosome arm and a total number of 87 in medaka, 74 in stickleback. Medaka and stickleback showed very similar patterns of synteny conservation with rainbow trout and a tight correspondence between medaka and stickleback linkage groups could easily be found.

**Table 1:** Syntenies found between rainbow trout linkage groups and medaka, stickleback and zebrafish chromosomes (figures correspond to chromosomes numbers of medaka, stickleback and zebrafish)

Rainbow trout linkage group	Arm number	Syntenic linkage group in		
		
		medaka	stickleback	zebrafish
RT01	1	12, 13, 14	groupVII, groupXIV	10, 15, 21

RT02	2	01, 08	groupIX, groupXI	01, 03, 06, 13, 22

RT03	2	10, 11	groupIV, groupX	12, 14, 16, 17, 19

RT04_25	2	01, 02, 10, 22	groupI, groupIV, groupXV	14, 17

RT05	2	21	groupXVI	09, 22

RT06	2	04, 06, 15, 19	groupV, groupVI, groupVIII, groupXIX	13, 17, 18, 22, 25

RT07	2	06, 17, 20, 23	groupIV, groupXXI	02, 04, 24

RT08	2	04, 12, 17	groupVIII, groupXIV	02, 06, 10, 17, 21, 22

RT09	2	08, 12, 14	groupVII, groupXI, groupXIII	03, 05, 10, 17, 21

RT10	2	03, 06, 09	groupII, groupXIII, groupXIX	05, 07, 08, 10, 18, 25

RT11	1	13	groupI	15, 17, 18

RT12	2	02, 04, 05, 07, 10, 21, 22	groupI, groupVIII, groupXII, groupXV, groupXVI, groupXVII	01, 02, 06, 08, 09, 11, 21, 22, 23

RT13	1	17	groupIII	02, 08, 17, 20

RT14	2	01, 02, 22	groupI, groupIX, groupXV	01, 17

RT15	2	06, 18, 23	groupIV, groupVII	04, 07

RT16	2	02, 03, 07, 11, 21, 22	groupI, groupII, groupX, groupXV, groupXVI	01, 02, 06, 09, 11, 12, 19, 22

RT17	2	15, 19	groupV, groupVI	07, 12

RT18	1	03, 06	groupII, groupXIX	07, 25

RT19	2	05, 09, 20, 21	groupXIII, groupXXI	02, 05, 08, 09, 11, 21

RT20	2	01, 14	groupI, groupVII, groupIX	01, 10, 17, 21

RT21	2	05, 07, 18	groupVII, groupXII	07, 08, 11, 23

RT22	2	07, 15, 19	groupV, groupVI, groupXII	08, 12, 13, 17, 23

RT23	2	17, 24	groupIII, groupXVIII	02, 11, 20

RT24	2	04, 05, 06, 08, 24	groupVIII, groupXI, groupXVIII, groupXIX	03, 06, 11, 17, 20, 23, 25

RT26	1	13	groupI	15, 18

RT27	2	06, 16	groupXIX, groupXX	04, 15, 16, 18, 25

RT29	2	01, 03, 05, 06, 07, 08, 15	groupVII, groupIX, groupXI, groupXII, groupXVII	01, 03, 05, 06, 08, 11, 13, 22, 23

RT30	1	15, 19	groupV, groupVI	05, 12, 13, 17

RT31	2	16, 21	groupXVI, groupXX	07, 09, 16

Total	52	87	74	126

Details are given in Additional file 5, Additional file 6, and Additional file 7

Duplicated regions of the rainbow trout genome showed conserved syntenies with the same chromosomes in each of the model species (Table 1 and Additional file 6, sheets 4, 8 and 12). Further evidence for duplication of the rainbow trout genome were revealed by the identification of additional sequences in the model species which had homology with sequences on two putative duplicated regions in rainbow trout. These sequences confirmed the presumptions of homeology between Omy6q and Omy11b and between Omy5a and Omy12p [22], bringing the total number of homeologies supported by two or more markers to 22 (Figure 1). In most cases, chromosome arm homologies had a one to one ratio, but we observed four cases where the ratio was one to two arms (Omy12p with Omy5a and Omy10p, Omy12q with Omy13q and Omy4a, Omy18q with Omy14q and Omy10p, and Omy10p with Omy12p and Omy18q).

### Affinities between rainbow trout and model species chromosomes and 3^rd ^WGD

Homeologies between chromosomes due to the 3^rd ^WGD have been detailed for medaka [12]. Homeology relationships between chromosomes in zebrafish and stickleback were retrieved from the Additional file 2: Table S2 (Duplicate genes in zebrafish, stickleback, medaka and fugu, derived from whole-genome duplication) provided by Lee *et al. *[28] (Additional file 6, sheets 6 and 10). When we considered the rainbow trout chromosome arms for which homologies with more than one medaka chromosome were found, these homologies involved two homeologous medaka chromosomes in more than half of the cases, i.e. 15 out of 24 (12 out of 24 if we do not account for chromosome arms Omy7p, Omy18p, Omy17q which stand out with regard to their complex patterns of homology with medaka chromosomes) (Additional file 6, sheet 2); for example, chromosome arm Omy1p (RT06) was homologous to Ola04 and Ola06 which are not homeologous and chromosome arm Omy1q was homologous to Ola15 and Ola19 which are homeologous. If we assume that the probability to sort the homeologous chromosome of a chromosome in medaka is 1/24 (where 24 is the chromosome number in stickleback), a proportion of 12 for 24 has the probability $\left(\begin{array}{c} 24\\ 12 \end{array}\right){\left(1/24\right)}^{12}{\left(23/24\right)}^{24}$ and the probability to observe 12 cases or more is $\sum_{k=12}^{24}\left(\begin{array}{c} 24\\ k \end{array}\right){\left(1/24\right)}^{k}{\left(23/24\right)}^{24-k}$, i.e. ≈ 5 × 10^-11^. This low probability suggests that these associations did not occur randomly or that the aforementioned cases do not really correspond to true translocations and neo-syntenies (see discussion). This conclusion is likely to hold for stickleback where similar numbers were found (Additional file 6, sheet 5).

### Alignment of conserved syntenies between model species and rainbow trout on the synthetic map

**Figure 2:**
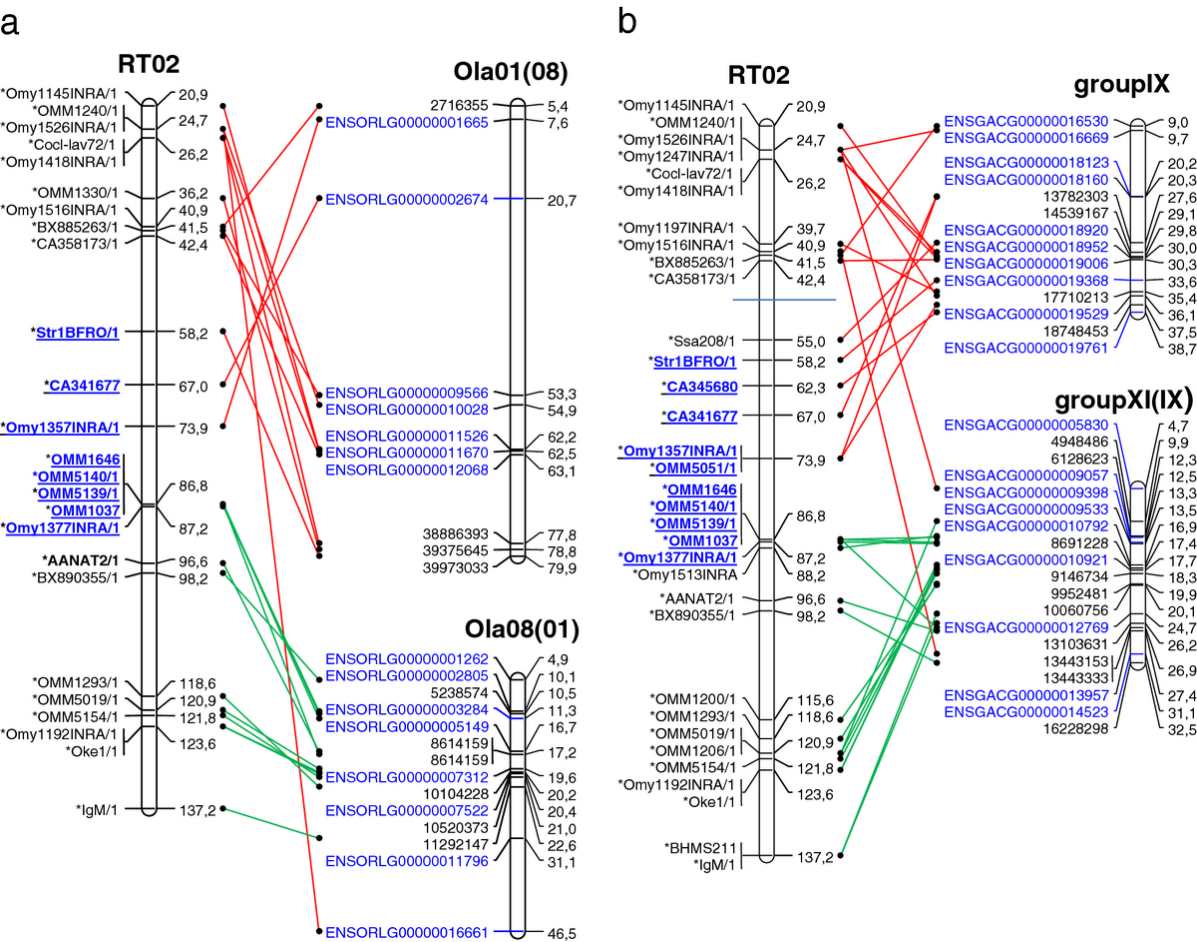
**Conserved syntenies between the rainbow trout RT02 linkage group and two homeologous chromosomes of the medaka (Figure 1a) and stickleback (1b)**. Ola01 and Ola08 in medaka, group IX and group XI in stickleback, result from the duplication of the same ancestral chromosome at the time of the 3^rd ^Whole Genome Duplication in fish. Centromeric regions are approximately delineated by underlined, blue bold type, marker names following [21]. Red and green lines identify the two chromosome arms of RT02. See Additional file 7, Additional file 8 and Additional file 9 for complete graphic representation of the conserved syntenies between rainbow trout and the three model species.

Shared homologies involving a rainbow trout arm and two medaka (or stikleback) homeologous chromosomes arisen from the 3^rd ^WGD could not be clearly interpreted (see above and discussion) and could reflect homology with only one of the two homeologs. Therefore, when such a situation occurred, only the medaka (or stickleback) homeolog (thereafter named "expected homologous chromosome") which had the highest number of sequence homologies with the rainbow trout arm was conserved in graphic representations of the alignments of the medaka or stickleback chromosomes to rainbow trout ones. For example, only groupVIII and groupVI were kept for RT06 in the comparison between rainbow trout and stickleback; homology of RT06 with groupV, which is homeologous to groupVI, was ignored. This led to parsimonious graphic representations of the genome evolution between rainbow trout and these two model species (Additional file 7 and Additional file 8). Alignment of the stickleback linkage groups on the rainbow trout map is partly represented in Figure 2. Under these conditions, despite the high number of chromosome arms in rainbow trout (n = 52), a rather limited number of syntenic fragments were found between this species and each of the two model species, medaka (68) and stickleback (63). In the case of zebrafish, which showed the highest number of synteny disruptions with rainbow trout, all the homologies based on more than one marker, *i.e*. 123, were conserved (Additional file 9). Localisation of centromeres on the rainbow trout map showed that, in many cases, a trout chromosome arm was syntenic to a single large chromosome fragment in medaka or stickleback. However, gene orders were poorly conserved between species in both the synthetic (Figure 2 and Additional file 7, Additional file 8 and Additional file 9) and the second generation INRA maps (data not shown).

**Figure 3:**
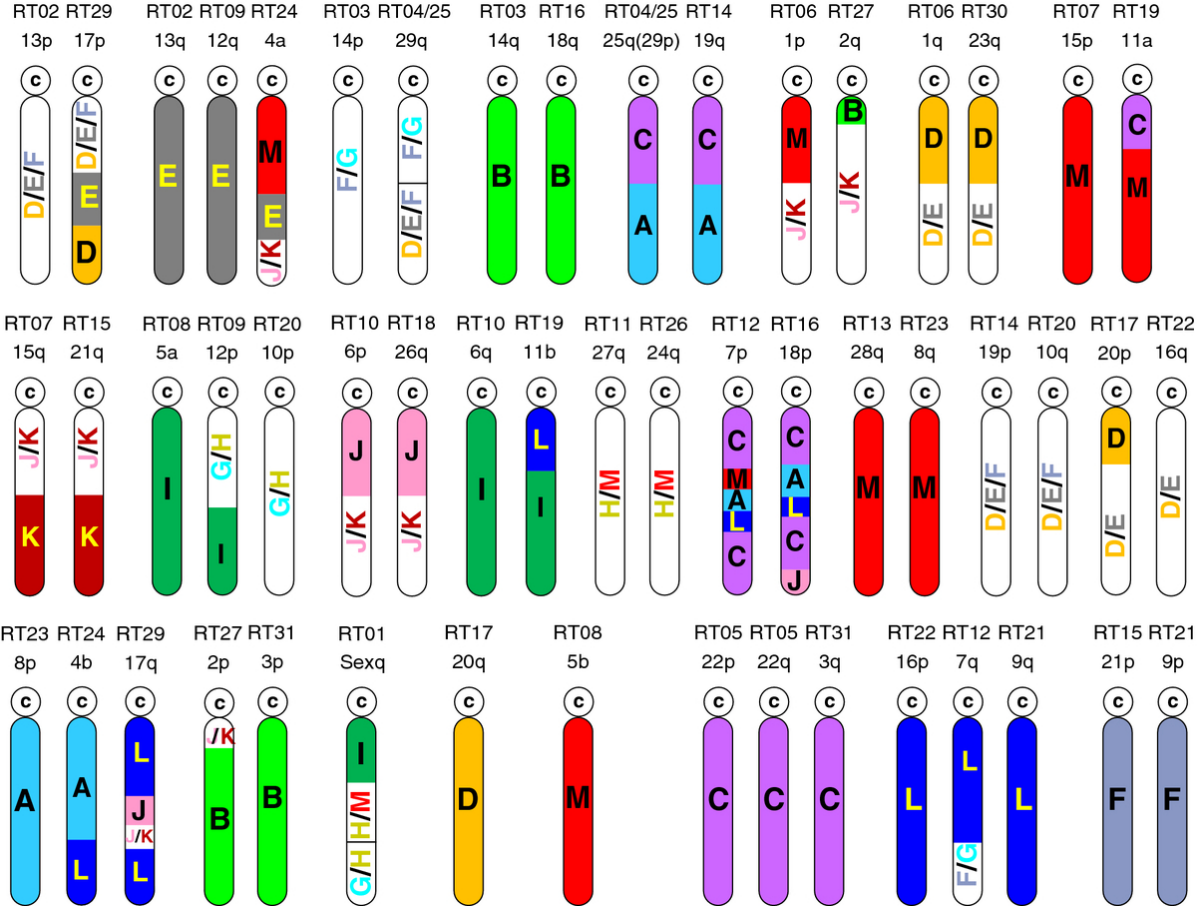
**Graphic representation of evolutionary relationships between rainbow trout chromosome arms and teleost ancestor proto-chromosomes**. In lines 1 and 2 (top to bottom), rainbow trout chromosome arms are arranged in identified homeologous pairs. In line 3 (bottom), chromosome arm pairing is based on putative homeologies (see text). Teleost ancestor proto-chromosome names and colours are the same as in [12].

Finally, since medaka has not undergone any major interchromosomal rearrangement after those occurred in the teleost ancestor, we used the chromosome affinities found between medaka and the reconstructed teleost ancestor genome [12] to recover the contribution of this teleost ancestor to the rainbow trout chromosome arms. Twenty chromosome arms, out of 52, were traced back to only one ancestral chromosome (Figure 3; Additional file 6, sheet 2; column H). In contrast, Omy7p(RT12), Omy18p(RT16) and Omy17q(RT29) were assemblages of 4 and 5 fragments of ancestor chromosomes (Figure 3; Additional file 6, sheet 2; column H)

## Discussion

A prerequisite to the construction of a synthetic map is the accuracy of segregation data and their consistency across the different sets. Here, we found that all the markers, except six, had the same linkage group assignment from one study to the other. We also found the same marker orders across the three studies for a high proportion of markers. Only 21 loci displayed important order differences between the three individual maps. Nevertheless, since comparisons for gene order were not done for approximately half of the total number of the synthetic map loci (namely, loci which could be mapped in one mapping parent only and for which no comparison was possible), a higher number among the studied markers could be localized at a wrong position. The comparison of marker orders between rainbow trout and the two closest model species, medaka and stickleback, shows that some markers could not be assigned to the expected chromosome arm in rainbow trout and need to be checked for their position. For example, it is the case for markers One1141DFG and OtsG83bUCD on RT08 in the rainbow trout *versus *medaka comparison (Additional file 7). Erroneous positions did not necessarily result from genotyping errors, but could also be due to the fact that the map was constructed from several segregation data files of different types. One way to avoid this drawback would be to start from individual male and female original data sets, to generate and process a single large table of raw genotype data. In this case, it would be possible to explore for marker orders which would be consistent across data sets.

Our results are in agreement with those reported by Danzmann *et al. *[22]. In this regard, we observed the same conserved syntenies between rainbow trout and medaka, with exceptions for RT12, RT19 and RT31. In the case of RT19 and RT31, the syntenies observed with Ola17 and Ola11 respectively are replaced by syntenies with their homeologous chromosomes (*i.e*., Ola20 and Ola16 respectively). Homologies based on a single marker in Danzmann *et al. *[22] were not confirmed by additional sequences in the present study, but a few previously reported syntenies between medaka and Atlantic salmon [22] were also observed between medaka and rainbow trout in this study.

Results presented here also confirmed the conclusions from previous studies showing that a high proportion of syntenies are conserved among teleosts [6,10-12,22]. As expected, the number of conserved syntenic fragments between two species was consistent with the phylogeny [29]. Few differences were found between the genomes of medaka and stickleback which are the two closest species in our comparisons [29]. At the opposite, a substantially larger number of conserved syntenic blocks were required to align the zebrafish genome to that of any of the other species.

The proportion of cases where two stickleback (or medaka) homeologous chromosomes were syntenic to the same rainbow trout chromosome arm was unexpectedly high. Several explanations could account for such a proportion. First, the orthologous sequence to the rainbow trout marker is present on the expected model species homologous chromosome, but was not detected, due to the current degree of accuracy of sequence assemblies or to the sensitivity and specificity of blast search settings used. In a few cases, the second best hit involved a sequence located on the expected homologous chromosome (*e.g*. the second hit for OMM1389 on RT04_25 corresponds to a sequence located on the expected chromosome in medaka, Additional file 6, sheet 2). Moreover, 60 of the loci which were found on the medaka homeologous chromosome, instead of the expected homologous one in the comparison between rainbow trout and the medaka, gave significant hits in the comparison between rainbow trout and stickleback. For one third of these loci, the most significant hit recognized a sequence located on the stickleback expected homologous chromosome. For example, in blastx against the medaka database, the most homologous sequence to BX084660 (RT03) was located on Ola16 instead of Ola11 (Additional file 6, sheet 2), while, in the case of stickleback, it was located on group X as expected (Additional file 6, sheet 5).

Secondly, conserved syntenic blocks of duplicates between species were often punctuated by differential retention of genes and the loss of a duplicated copy on one homeolog was not always consistent across species. This would be facilitated when genes have evolved very slowly or if some tetrasomy has occurred and persisted for a long period after speciation in some lineages. For example, residual tetrasomy still exists in salmonids despite the fact that the 4^th ^WGD is dated to late cretaceous-early tertiary, c.a. 50-100 My ago [22].

Alternatively, this relatively high number of rainbow trout chromosome arms showing affinities with two homeologous medaka (or stickleback) chromosomes could be explained by the fact that exchanges between homeologous chromosomes resulting from the 3^rd ^WGD would have been quite common. Such exchanges have been reported in polyploids, including fish [30]. Under this assumption, the occurrence of rainbow trout chromosomes arms showing conserved syntenies with two homeologous chromosomes of a model species would reflect true inter-chromosomal rearrangements.

Sequence alignments reported in the present study (Additional file 7, Additional file 8 and Additional file 9) clearly reflect the major lines of chromosome evolution between the different species. Several studies have shown that, in medaka, stickleback and tetraodon, few major inter-chromosomal translocations have occurred after speciation in the three species and that the genomes of the well studied teleost species have remained remarkably stable, with the exception of zebrafish. No major inter-chromosomal exchange has occurred in the medaka genome and only three in tetraodon [6,10,12]. In contrast, a high number of inversions seem to have occurred in these species. The pattern of genome evolution which emerged here for rainbow trout is in agreement with previously reported results [21,23]. We found that 39 rainbow trout chromosome arms, out of 52, were homologous to only one single medaka chromosome and to its homeologous in some cases (Additional file 6 and Additional file 7). Fifteen out of 20 pairs of duplicated rainbow trout chromosome arms revealed that the two homeologous linkage groups had the same pattern of homology with medaka chromosomes (Additional file 6, sheets 2 and 4). This suggests that most of the inter-chromosomal exchanges occurred before the WGD specific to the salmonid lineage. Altogether, these findings also support the hypothesis that the salmonid chromosome arms have experienced few exchanges and that the evolution of salmonid chromosomes has mostly occurred through Robertsonian translocation [31,32].

This relative genome stability in the teleost lineage during evolution has made it possible to recover the contribution of the ancestral teleostean proto-chromosomes to those of the extant species and the traces of the 3^rd ^WGD. Since the medaka genome did not undergo inter-chromosomal exchange [12], we used it to find affinities between rainbow trout linkage groups and teleostean proto-chromosomes (Additional file 6, sheets 2 and 4). We also compared our findings to those reported in Danzmann *et al. *[22]. Reconstituting of the contribution of ancestral teleostean chromosomes to the rainbow trout ones was identical in the present study and Danzmann *et al. *[22] for 10 linkage groups. No true discrepancy was noticed between the two studies. Differences resulted from either additional homologies found in Danzmann *et al. *[22], but supported by a single sequence only, or additional homologies found in the present study. Overall, the rainbow trout genome seems to have undergone more rearrangements than medaka, stickleback or tetraodon in a shorter evolutionary time span. This could eventually reflect an acceleration of the chromosome rearrangement rates in salmonids after a whole genome duplication, an hypothesis which is still debated [33,34].

Due to the additional WGD in the salmonid lineage, each ancestral linkage group should be represented at least four times in the rainbow trout genome, with the exception of linkage group M which seems to have been triplicated in the ancestral teleost [35] and should be represented six times at least. This number is necessarily higher because of subsequent translocations before the teleost radiation and in the salmonid lineage. Since a fragment of one of the triplicates of the ancestral chromosome M has been translocated to one of the two copies of ancestral group H ([12,35]), eight salmonid chromosome arms are expected to show traces of linkage group M, which is the observed number. In the same way, due to partial translocations, a minimum expected number of 6 should be observed for ancestral groups D, E and F. This expectation is compatible with our results since we found a total of 18 rainbow trout chromosome arms displaying homologies with D, E or F. Two ancestral groups, C and L, were found in more than 4 rainbow trout linkage groups, 8 and 7 respectively. In each case, more than one translocation in the salmonid lineage is required to explain these patterns.

Alignment of the model species chromosomes to the rainbow trout linkage map (Figure 3) provided additional information on the homeologous affinities within the rainbow trout genome due to the salmonid-specific WGD. First, homology searches resulted in the uncovering of additional common sequences within a few pairs of duplicated linkage groups. Thus, we consolidated the homeologies previously described [21-23]. Five homeologies (Omy5a(RT08) and Omy12p(RT09), Omy6q(RT10) and Omy11b(RT19), Omy14p(RT03) and Omy18q(RT16), Omy1p(RT06) and Omy2q(RT27), Omy15p(RT07) and Omy11a(RT19) which were suggested or supported by a single marker in Danzmann *et al. *[22] were further confirmed by the mapping of additional duplicated markers to these regions in the current study. Finally, we reported two new putative homologies supported by two or more loci: Omy18q(RT16) and Omy10p(RT20), Omy4b(RT24) and Omy17q(RT29), Second, putative homeologous pairs resulting from the 4^th ^WGD and identified on the basis of shared duplicated loci had identical or very similar patterns of contribution from the same ancestral proto-chromosomes in all cases, with the exception of pair Omy18q(RT16)/Omy10p(RT20). For example, we found a very similar ancestral contribution between Omy25q(RT04_25) and Omy19q(RT14) which shared only three common microsatellite loci (Figures 1 and 3). Although such homologies could also result from more ancient duplications, they are more likely to reflect the most recent whole genome duplication. Accordingly, the rainbow trout chromosome arm affinities which emerged from the sequence homologies with either medaka or stickleback and affinities with the teleost ancestor chromosomes provide some clues regarding potentially duplicated chromosome arms in rainbow trout, such as Omy22p(RT05)/Omy22q(RT05)/Omy3q(RT31), Omy16p(RT22)/Omy9q(RT21) and Omy21p(RT15)/Omy9p(RT21) (Figure 3). Future dense mapping and genome assembly projects would allow validating these assumptions.

## Conclusions

In the present study, we reported a method to generate a synthetic map using very composite raw data files. This approach is iterative and the present synthetic map could be easily incorporated to any new data set following the same method. When possible, original raw data files rather than reconstructed ones should be used. This synthetic map will represent a valuable genomic resource for QTL fine mapping and for improving assembly of the rainbow trout genome sequence, provided that care is taken to explore the possible alternative gene orders in the regions under investigation and avoid misleading orders. The present study strongly supports the view that, overall, large blocks of synteny have been conserved for most chromosomes between rainbow trout, stickleback and medaka. In contrast, intra-chromosomal rearrangements seem to have been very frequent. This finding is corroborated by the moderate proportions of conserved microsyntenies found between rainbow trout and the same model species [19]. Altogether, these observations suggest that comparative mapping between rainbow trout and model species remains a valuable approach to identify candidate genes in functional studies, despite the fact that the occurrence of intra-chromosomal micro-arrangements should, to some extent, make difficult to accurately and rapidly predict the location of such genes.

## Methods

### Update of the INRA linkage map

#### Mapping families

Reference families used for linkage mapping are the same two doubled haploid (DH) lines as previously described [21]. Centromere mapping was established using the same meiotic gynogenetic line as previously described [21].

#### Microsatellite markers

Sequences of markers previously used for linkage mapping were extracted from NCBI databases. DNA extraction and genotyping methods for newly added microsatellite markers were previously described [21].

#### Single nucleotide polymorphisms

EST sequences were extracted from NCBI or SIGENAE http://www.sigenae.org EST databases. They were masked for repeated sequences using Mreps 2.5 [36] and the cGRASP salmonid specific repeat masker [37]. Masked EST sequences were blasted against zebrafish genomic database in order to localize the position of intronic region and to avoid designing primer pairs encompassing large intronic sequences or two exonic region extremities. Primers for PCR amplification and sequencing were designed with Primer3 [38]. PCR products showing single band pattern after migration in agarose gels were sequenced for SNP detection in female parents and grandparents of the two DH lines. Purified PCR products were sequenced using ABI Prism Big Dye v3.1 Terminator Cycle sequencing Kit (*Applied Biosystems*). DNA sequences were then analysed using an ABI 3730 DNA Analyzer (*Applied Biosystems*). SNP or indel polymorphisms were detected by aligning female and grandparents sequences. Since grandparents were DH homozygous individuals, true polymorphisms at one locus could be distinguished from fixed single nucleotide differences between duplicated loci. DH progenies were genotyped using the SNPlex genotyping technology (Applied Biosystems).

#### Linkage map construction

Linkage groups were constructed using CARTHAGENE software [39] and optimized with the Annealing option (argument values: 15, 300, 0.1, 0.5) (see Carthagene help for argument meaning). Since interference is close to one in salmonids, we used the percentage of recombination as mapping function. Graphical representations were obtained with MAPCHART [40]. Chromosome arms were identified as previously described [41] except for Omy5 (RT08), Omy11 (RT19) and Omy4 (RT24) for which no arm correspondence could be clearly established.

### Screening of linkage data and construction of a synthetic map

To construct the synthetic map, we used raw segregation data from INRA (this study), linkage data from UoG (Additional file 3, Additional file 4, Additional file 5 and Additional file 6 in [22]) and ARS (Additional file 1, sheet 11 in [23]). In total, six data sets were handled, one for INRA, one for ARS and four for UoG (UoG_F25, UoG_M25, UoG_F44 and UoG_M44). Linkage data files were loci listed per linkage group. Each locus was associated with a recombination rate to the next locus in UoG files, to a Kosambi distance in ARS file.

#### Standardisation of locus names and accession numbers

Depending on authors, different locus names and accession numbers were used, precluding the identification of identical loci in some instances. Here, we used the original locus names, except for some short names which received a suffix to avoid misidentification and to comply with the current nomenclature (*e.g*.: Ocl1UW instead of Ocl1 or Ssa16DU instead of Ssa16). Duplicated loci were distinguished by addition of suffixc /1 or /2 to the locus name. Marker and accession number synonymies were checked through database queries and blastn.

#### Consistency of linkage maps

Ee checked consistency of linkage assignments and marker orders across the six data sets. Groups of loci which were not unambiguously assigned to one single linkage group in UoG files (e.g. RT05 + 31f, Additional file 3 in [22]) were discarded. When a discrepant linkage group assignment or position in the linkage group was observed for a locus in a data set with respect to the other ones, this locus was removed from this data set. When assignments to different linkage groups or when two different positions in the same linkage group were equally possible, the two possibilities were kept and an additional suffix identified the origin of the data (ARS, INRA or UoG).

#### Construction of the synthetic linkage map

In ARS file, Kosambi distances between adjacent loci were converted into recombination rates using the formula r = 0.5(e^4k^-1)/(e^4 k ^+ 1); gamete vectors were then recovered by converting the recombination rates associated to the loci into lines of zeros and ones; the total number of 0 and 1 on the line equalled the size of the mapping family/line. For example, the first locus in any linkage group corresponded to a line of 25 zeros followed by 25 ones for a family size of 50 offspring (see Additional file 1, sheet 3 (UoG_F25) for example). If the recombination rate between the first and the second locus was 4%, two recombinants (4% × 50 offspring) were introduced between the two loci by changing the first zero to one and the first one to zero in the line corresponding to the second locus (see Additional file 1, sheet 3 for example). This procedure was iterated along the linkage group. Family/line sizes were those given in [21-23], except for UoG_F44 and UoG_M44 which were assigned a size of 50 individuals each (about the same size as the two other UoG families) instead of 86 to avoid a too high weight of the UoG data. Linkage groups were constructed one by one by merging the individual data sets with the Mergen option of CARTHAGENE. Marker orders in linkage groups were optimized with Flips (argument values: 5, 0, 1), Taboo (1, 0, 1, 15, 0) and Annealing (15, 300, 0.1, 0.5) options in CARTHAGENE. Centromere position ranges were primarily derived from [21].

### Rainbow trout sequence homology searches and filtration of results

Rainbow trout sequences were masked as described in [19] using RepeatMasker http://www.repeatmasker.org/ and the two repeat databases, Salmon Raw Repeat DB V1_6 available at http://web.uvic.ca/grasp and INRA Rainbow Trout Rep1.0. Masked sequences were analysed for sequence homology by BLASTN using ENSEMBL DNA databases for zebrafish (Danio_rerio.Zv9.61.dna_rm.toplevel.fa), stickleback (Gasterosteus_aculeatus.BROADS1.61.dna_rm.toplevel.fa), and medaka (Oryzias_latipes.MEDAKA1.61.dna_rm.toplevel.fa) and for gene content by BLASTX using the ENSEMBL non redundant protein databases for zebrafish (Danio_rerio.Zv9.61.pep.all.fa), stickleback (Gasterosteus_aculeatus.BROADS1.61.pep.all.fa), and medaka (Oryzias_latipes.MEDAKA1.61.pep.all.fa).

Blastn and blastx searches were carried out using an e-value cut off of 1E^-5^. Blastn searches were carried out with the following parameters: -m9 -r1 -q-1 -G4 -E2 -W9 -F "m D" -U. The blastn search results were filtered to remove non specific sequences using the following filtration steps: (1) for each sequence read with blastn hit, results were filtered to keep only the hits with the minimal e-value score; (2) sequence reads with several hits having the same minimal e-value were further filtered to keep the hits with the highest HSPs (high-scoring segment pairs; calculated as the product of % identity multiplied by alignment length); and (3) only sequence reads with single hits following filtration steps 1 and 2 were kept.

For blastx, the Ensembl protein IDs were renamed by their corresponding Ensembl gene IDs (as each gene may encode several peptides due to alternative splicing) and then subjected to the above filtration steps.

### Alignment of rainbow trout linkage groups and model species genomes

Rainbow trout linkage groups were aligned with the chromosomes of each of the three model species using results of the sequence homology searches. To obtain an adequate graphical representation of linkage groups with MAPCHART, we estimated genetic distances between markers in cM by the nucleotide position on the model species chromosome divided by 0.5 × 10^6 ^(this conversion rate was preferred to the usual rate of 10^6 ^bp per cM because it allowed us a more convenient graphic representation). Marker names on these reconstituted linkage groups were the name of the gene Ensembl ID when a protein was identified, otherwise the start position of the sequence on the model species chromosome. Correspondence between medaka and teleost ancestor linkage groups were found in [12].

## Authors' contributions

RG conceived the study, carried out the linkage maps and drafted the manuscript. MB carried out the blast searches and revised the manuscript. EQ contributed to the conception of the study and the draft of the manuscript. FK and CH carried out the production and analysis of SNP and microsatellite data. All authors approved the final manuscript.

## Supplementary Material

Additional file 1 **Raw INRA, ARS and UoG genotype files (worksheets 2 to 7); loci discarded in each data set (worksheet 8); loci located at the same linkage group and showing two different positions in two data sets (worksheet 9); new loci added in the updated INRA map (worksheet 10); full legends are given in worksheet 1**. Click here for file

Additional file 2 **Accession numbers for loci used in the INRA and synthetic maps (worksheet 2); data files to produce the INRA and consensus graphical maps with MAPCHART (worksheets 3, 4 and 5); comparison of marker orders in the INRA and synthetic maps for each linkage group (worksheets 6 to 34); oxford grid showing homeologous affinities between rainbow trout chromosome arms (worksheet 35); full legends are given in worksheet 1**. Click here for file

Additional file 3 **Graphic representation of the updated INRA map; duplicated loci are in red bold type; underlined bold type marker names localize centromeric regions**. Click here for file

Additional file 4 **Graphic representation of the synthetic map (same captions as in Additional file **3). Click here for file

Additional file 5 **Significant blast results found in this study, in Danzmann *et al. ***[22]**and Rexroad *et al. ***[23]; **see worksheet 1 for full legends**. Click here for file

Additional file 6 **Conserved syntenies from between rainbow trout and, respectively, medaka, stickleback and zebrafish (worksheets 2, 5 and 9); Oxford grids showing chromosome homologies within stickleback and zebrafish (worksheets 6 and 10); files used to generate map alignments between rainbow trout and each of the three model species (worksheets 3, 8 and 11); Oxford grids showing homologous affinities between the rainbow trout arm chromosomes and the chromosomes of each model species (workheets 4, 7 and 12); full legends in worksheet 1**. Click here for file

Additional file 7 **Map alignments between chromosomes of rainbow trout and zebrafish; underlined blue bold type marker names approximately localize centromeric regions; green and red lines distinguish between the two arms in acrocentric rainbow trout chromosomes; homologous marker positions in model species chromosomes are identified by Gene ID and sequence start position when blastx hits and blastn hits are used respectively**. Click here for file

Additional file 8 **Map alignments between chromosomes of rainbow trout and medaka, stickleback and zebrafish; underlined blue bold type marker names approximately localize centromeric regions; green and red lines distinguish between the two arms in acrocentric rainbow trout chromosomes; homologous marker positions in model species chromosomes are identified by Gene ID and sequence start position when blastx hits and blastn hits are used respectively**. Click here for file

Additional file 9 **Map alignments between chromosomes of rainbow trout and medaka, stickleback and zebrafish; underlined blue bold type marker names approximately localize centromeric regions; green and red lines distinguish between the two arms in acrocentric rainbow trout chromosomes; homologous marker positions in model species chromosomes are identified by Gene ID and sequence start position when blastx hits and blastn hits are used respectively**. Click here for file
