# Supplementary figures and images for: A synthetic rainbow trout linkage map provides new insights into the salmonid whole genome duplication and the conservation of synteny among teleosts
Source: BMC Genet. 2012 Mar 16;13:15. doi: 10.1186/1471-2156-13-15 (PMC3368724; doi:10.1186/1471-2156-13-15)

## RT02

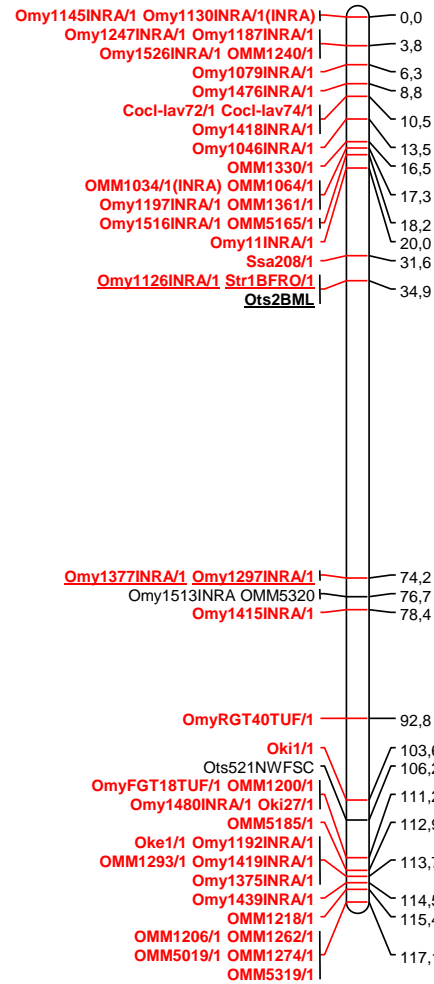

## RT01

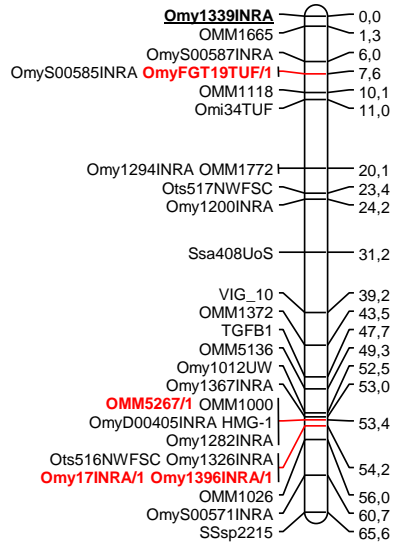

## RT03

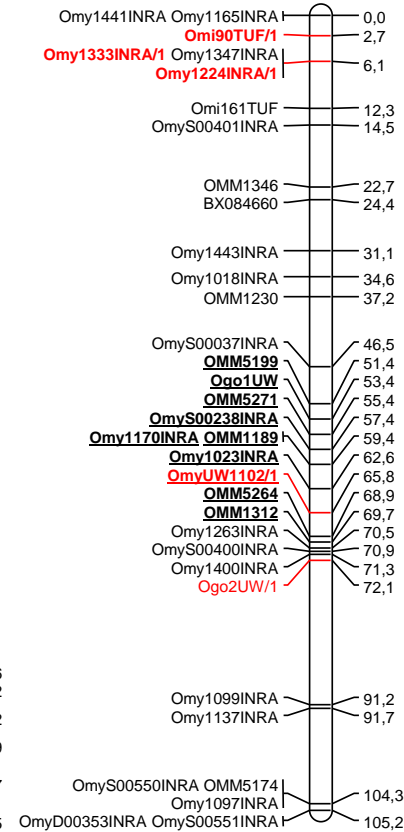

## RT04\_25

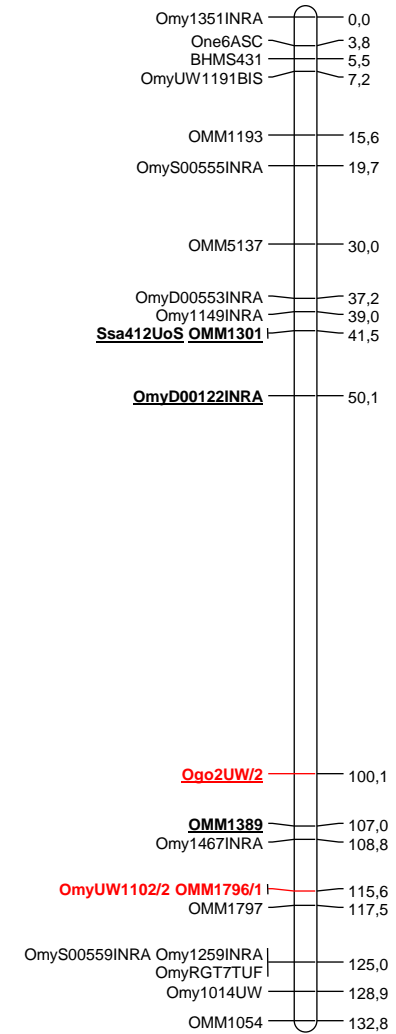

## RT06

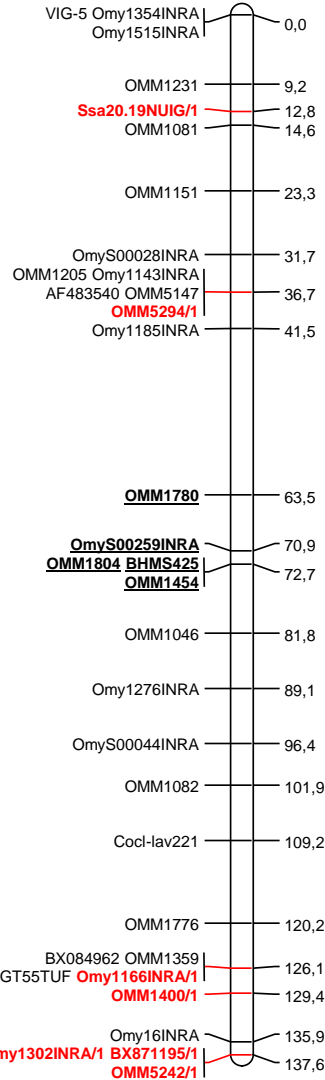

## RT07

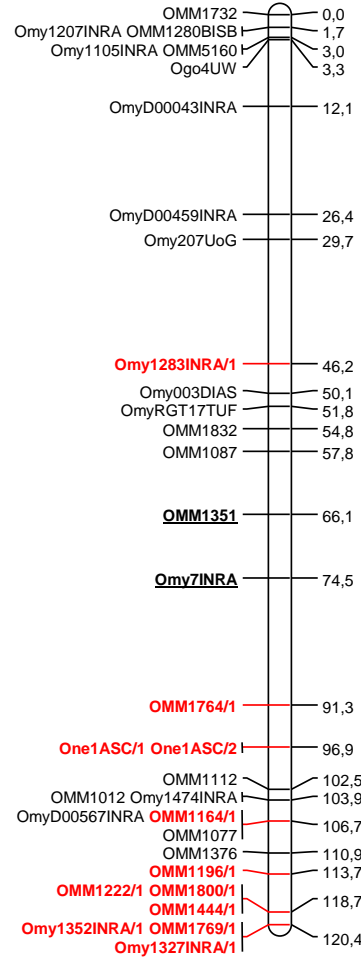

## RT08

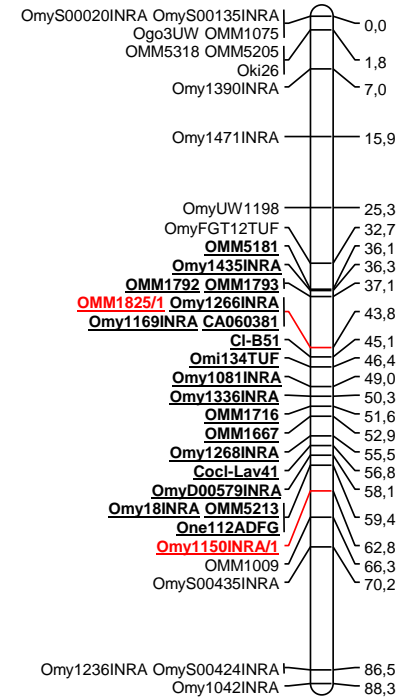

## RT05

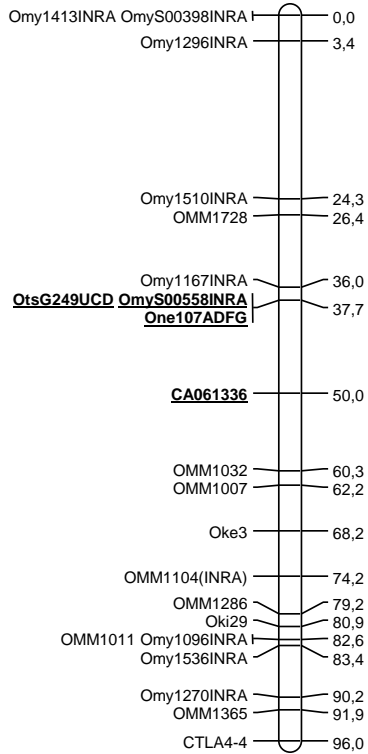

## RT05

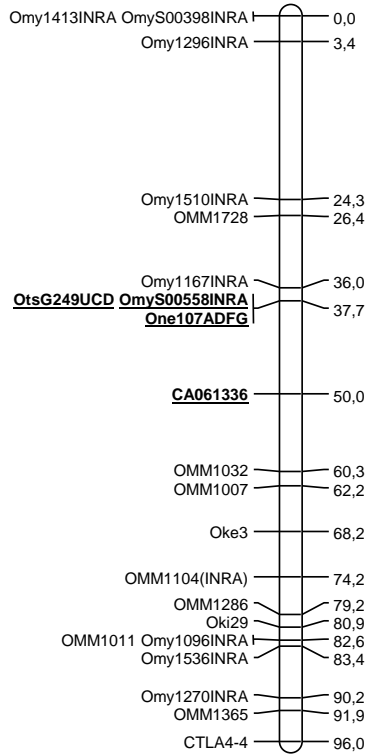

## RT06

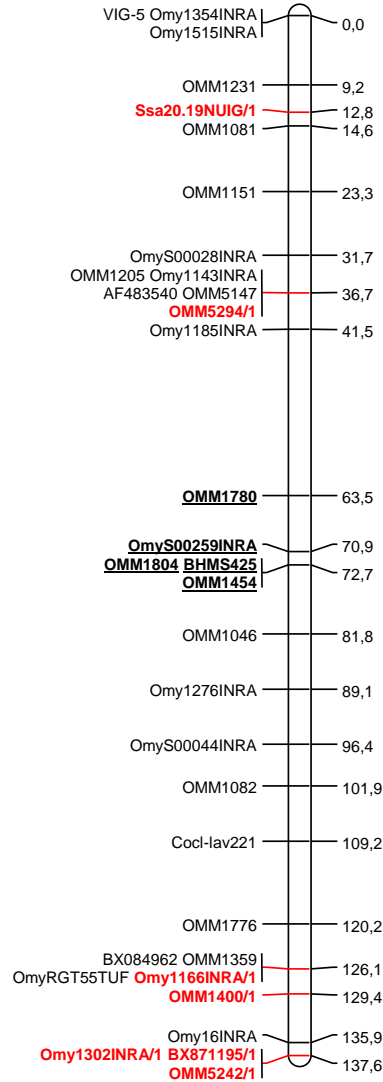

## RT07

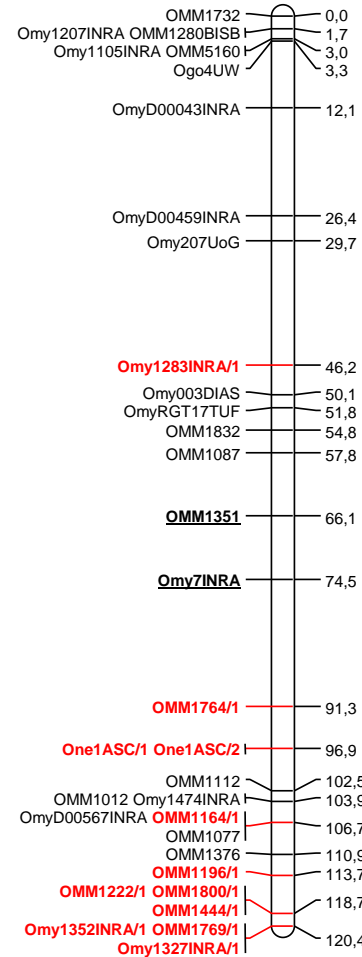

## RT08

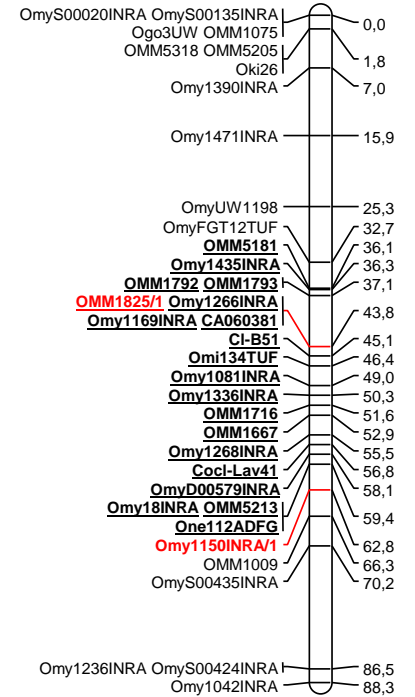

## RT09

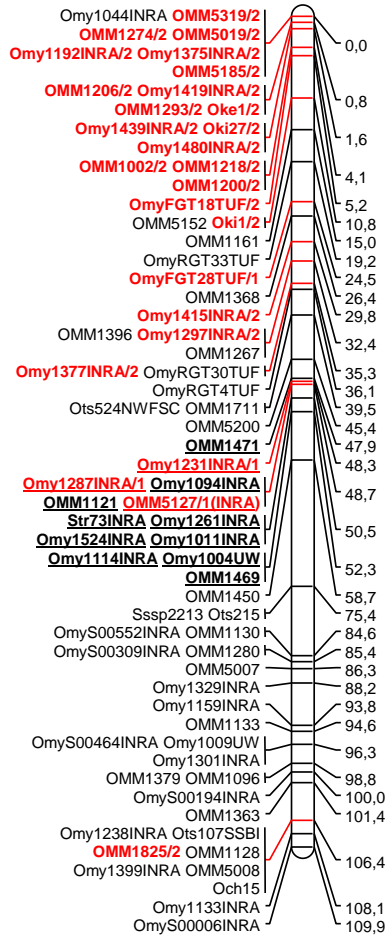

## RT10

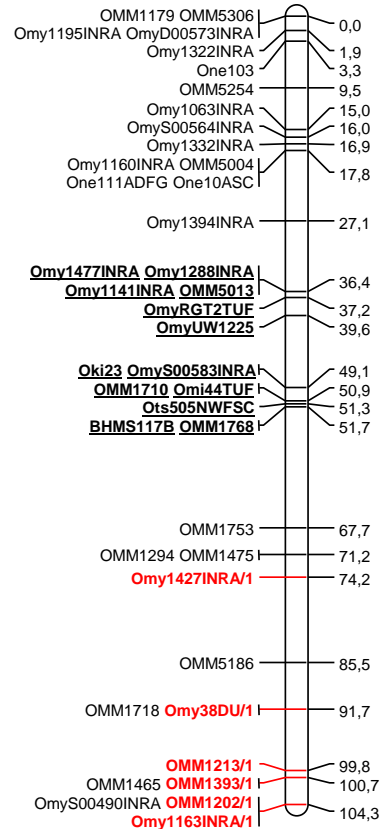

## RT11

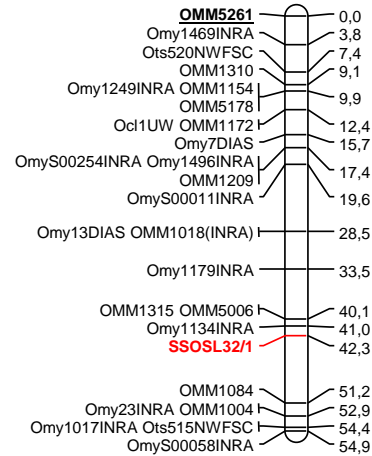

## RT12

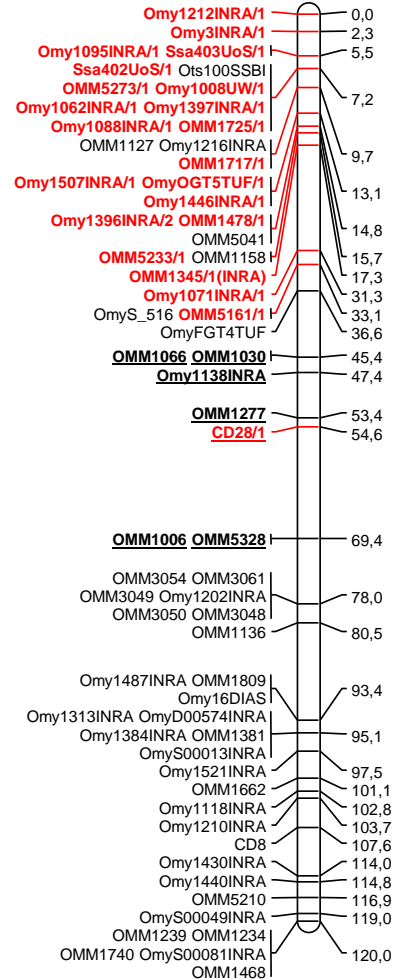

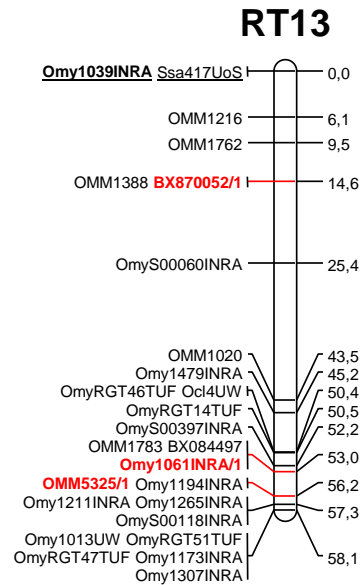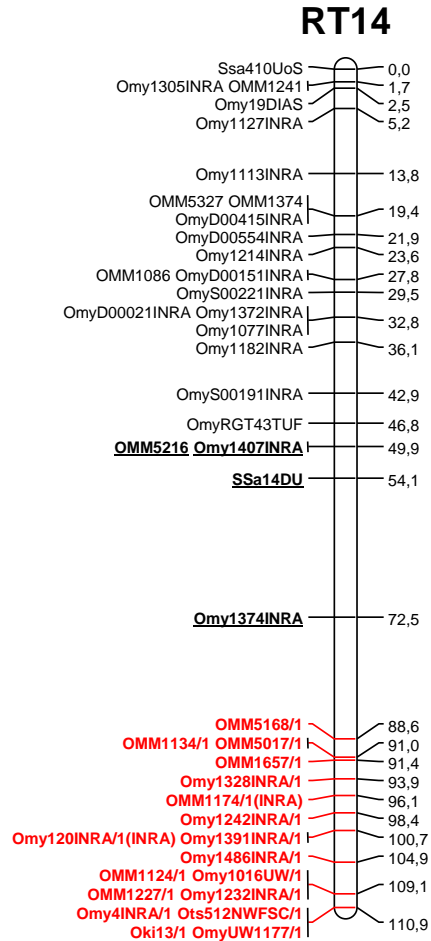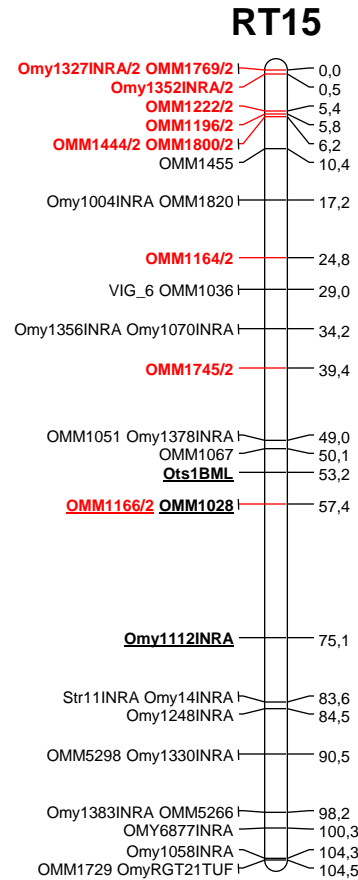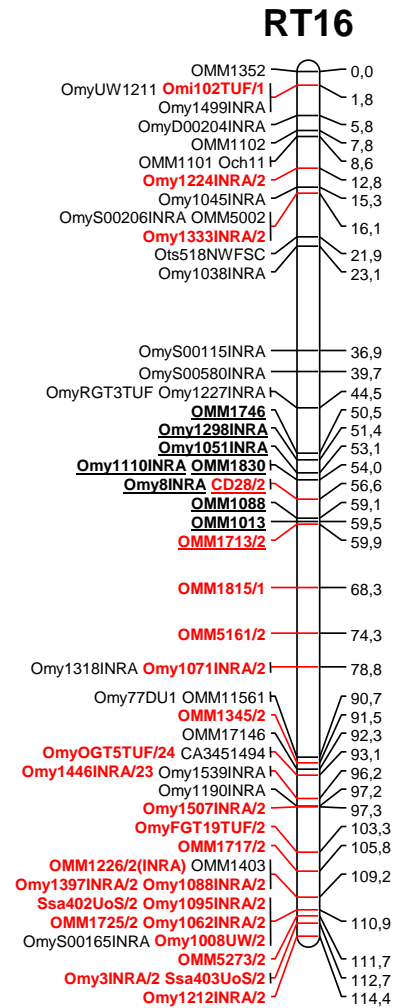

## RT17

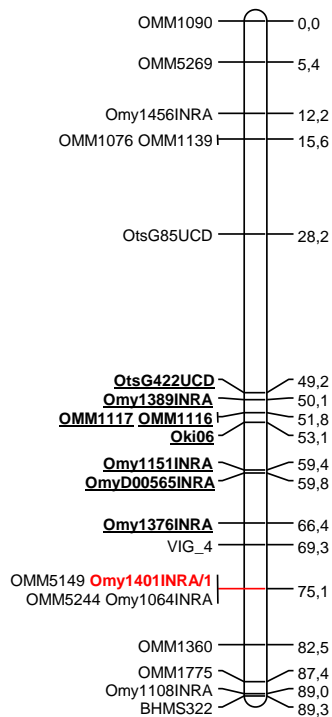

## RT18

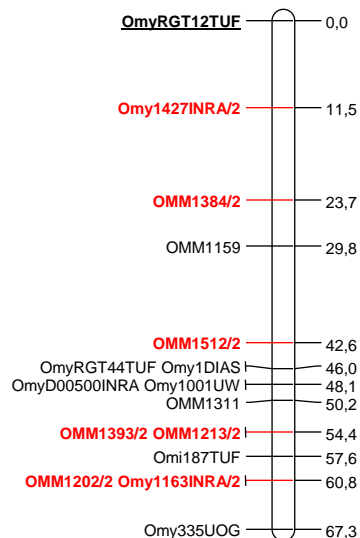

## RT19

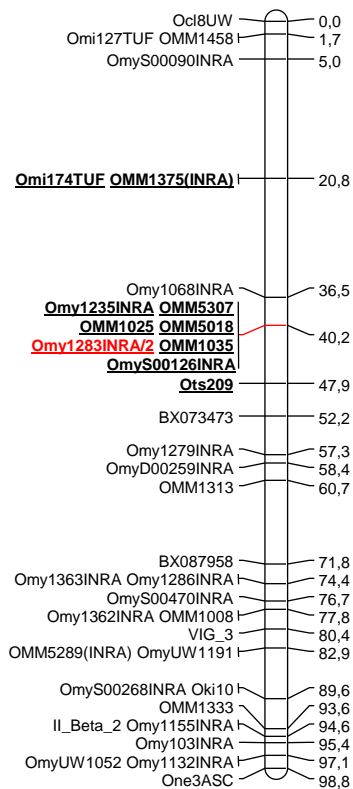

## RT20

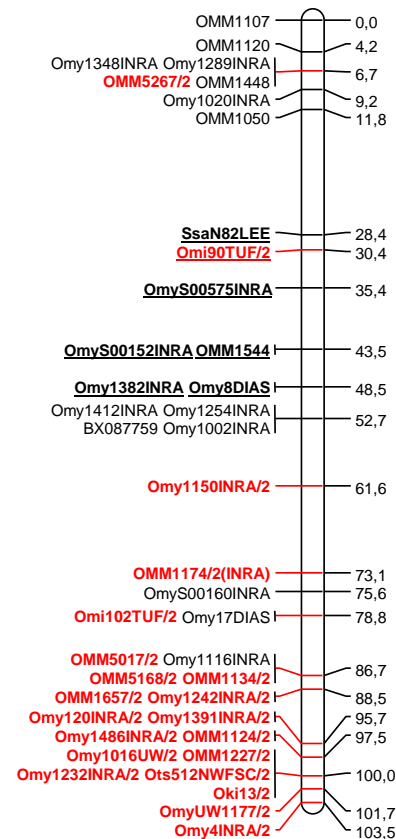

## RT21

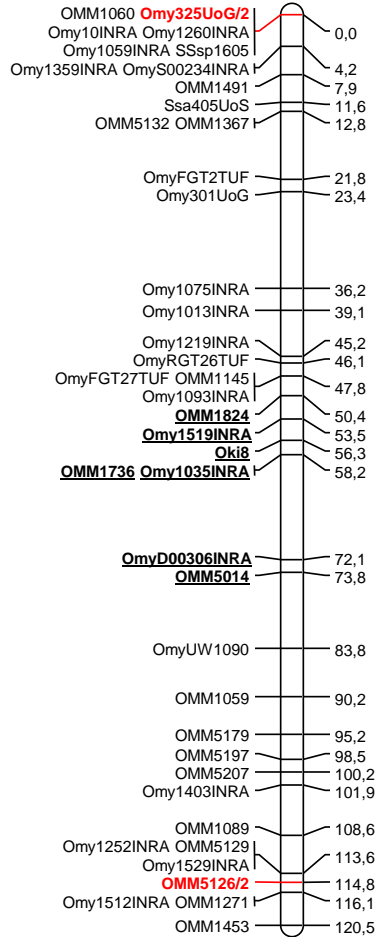

## RT22

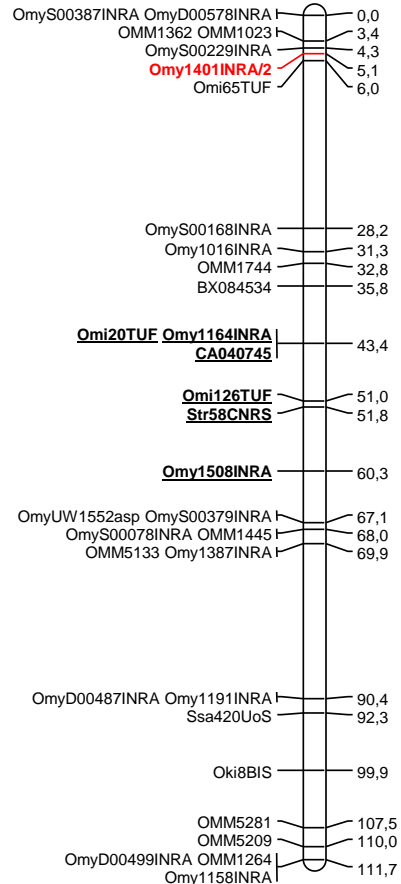

## RT23

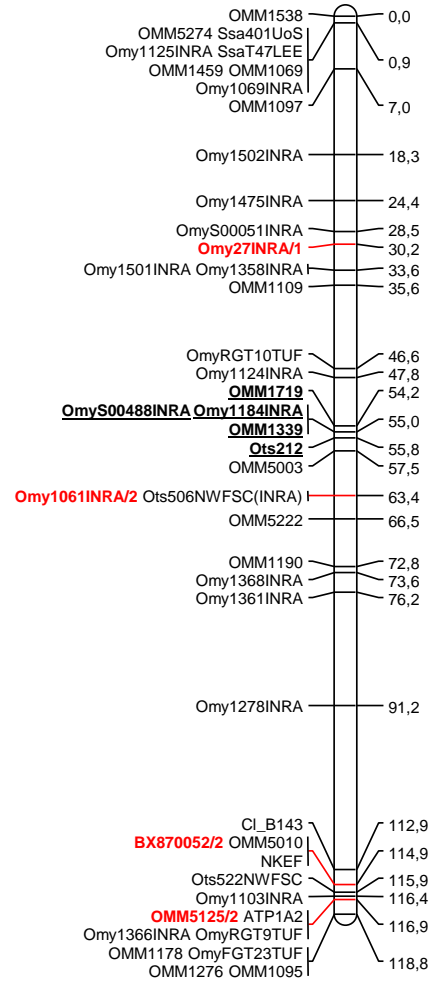

## RT24

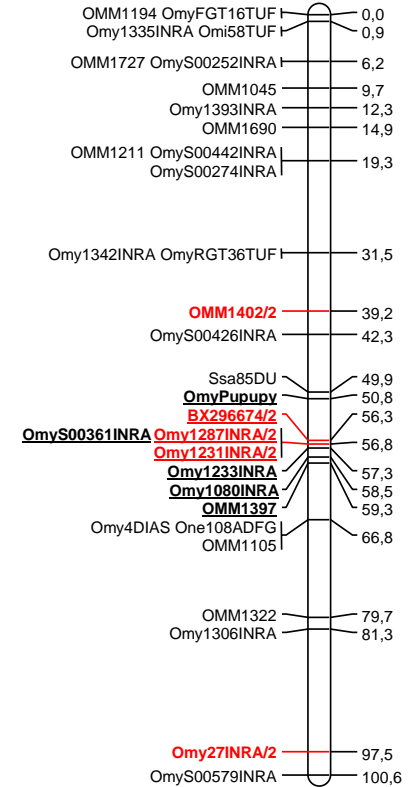

## RT26

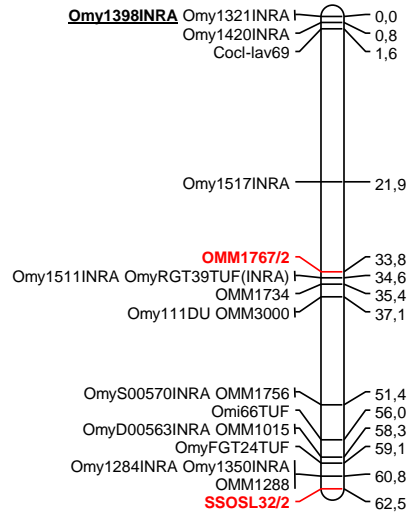

## RT30

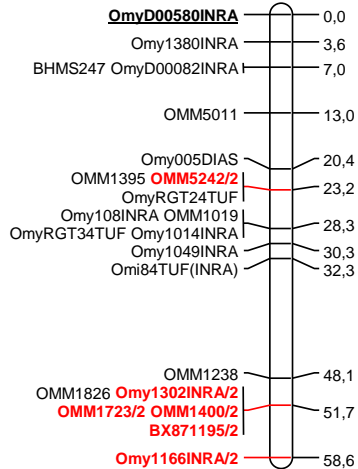

## RT27

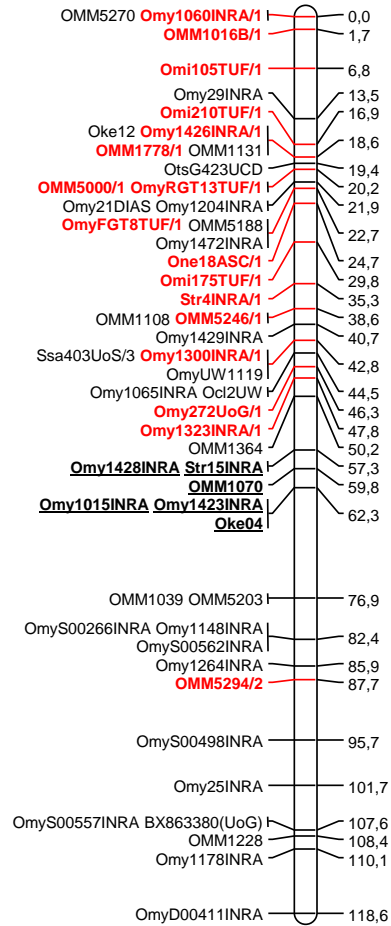

## RT29

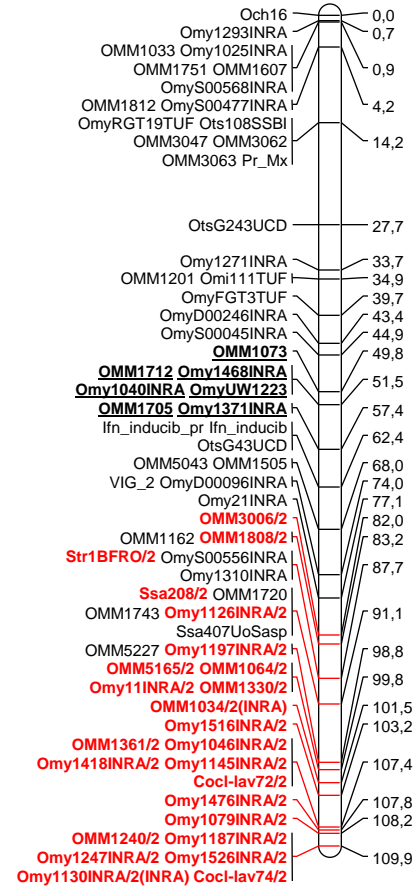

## RT31

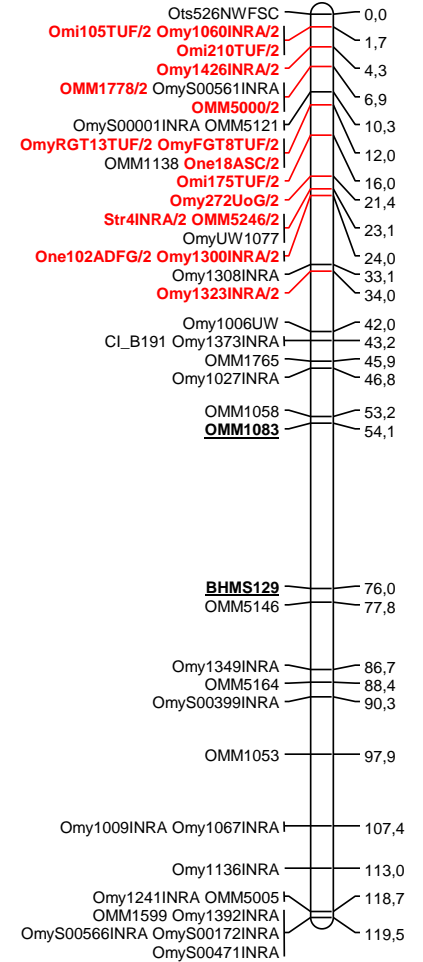

Supplement: Additional file 3 — Graphic representation of the updated INRA map; duplicated loci are in red bold type; underlined bold type marker names localize centromeric regions. [file 1471-2156-13-15-S3.PDF]

# RT03

## RT01

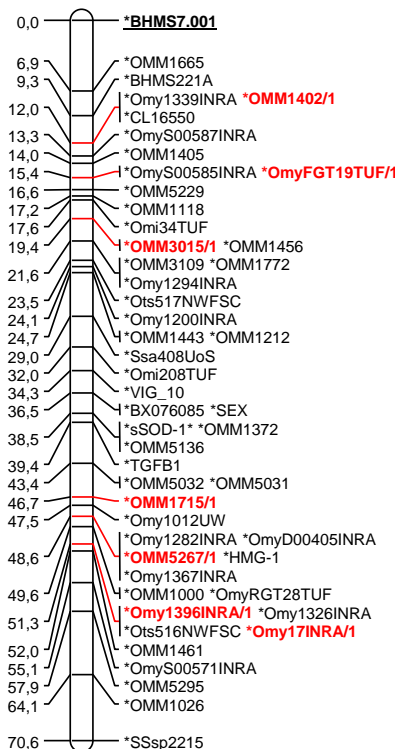

## RT02

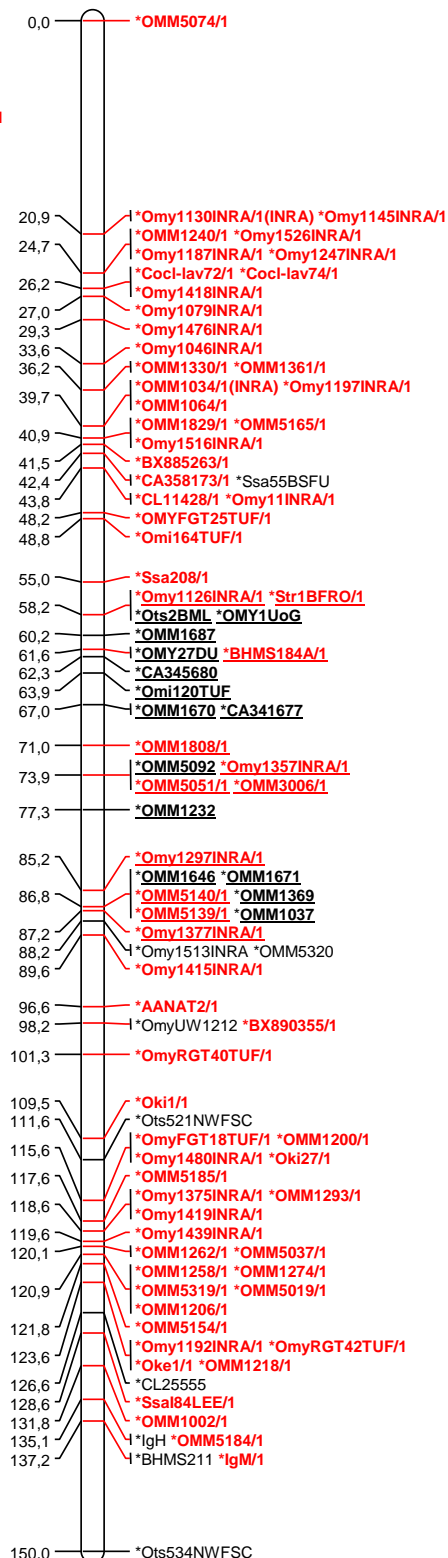

## RT05

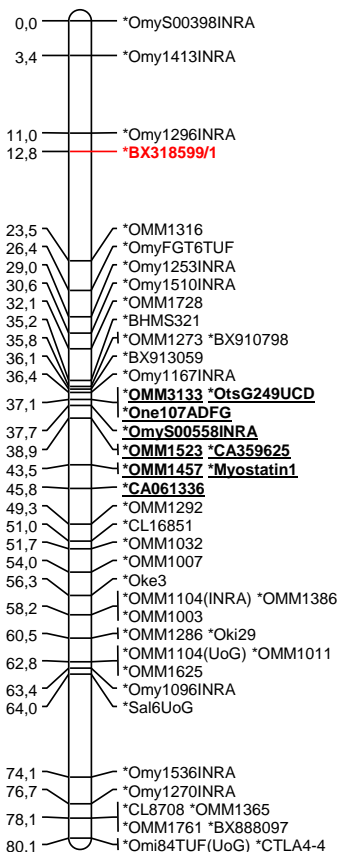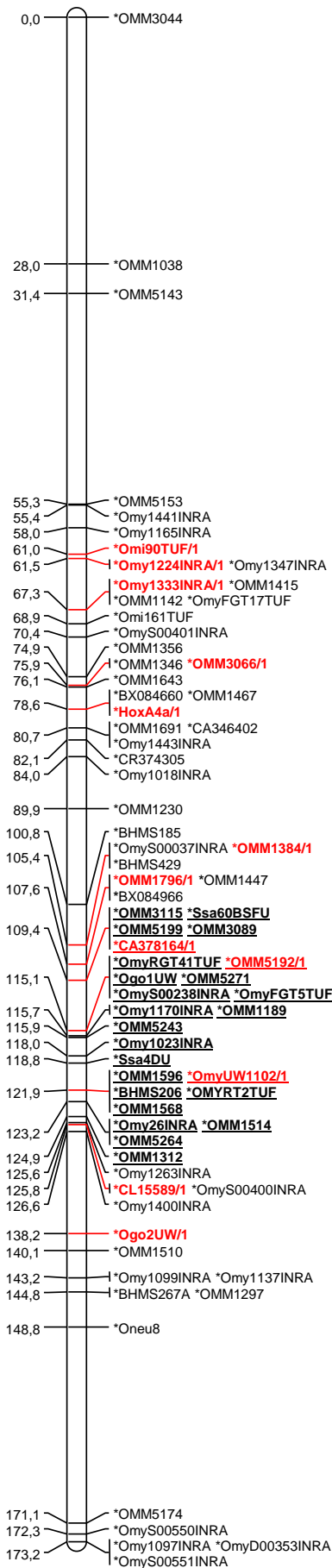

## RT04\_25

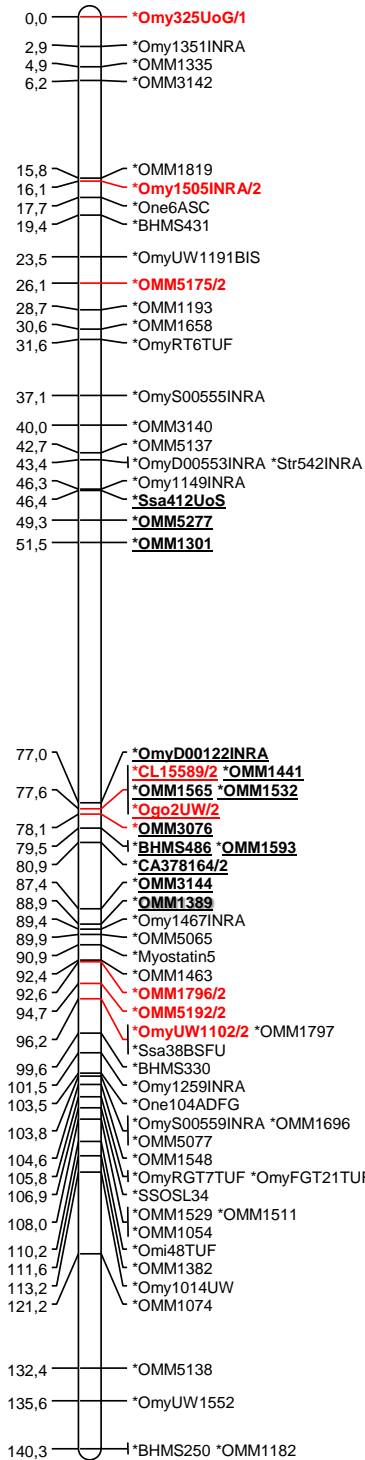

## RT06

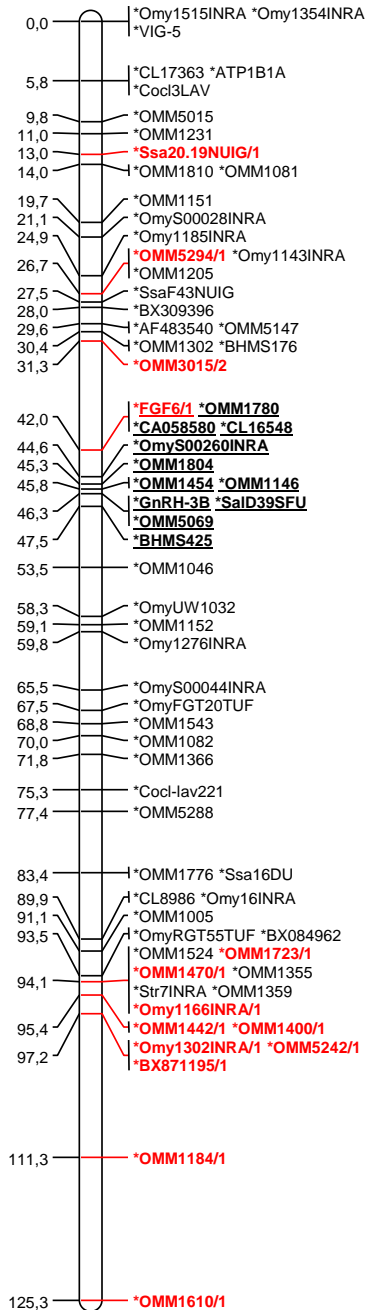

## RT07

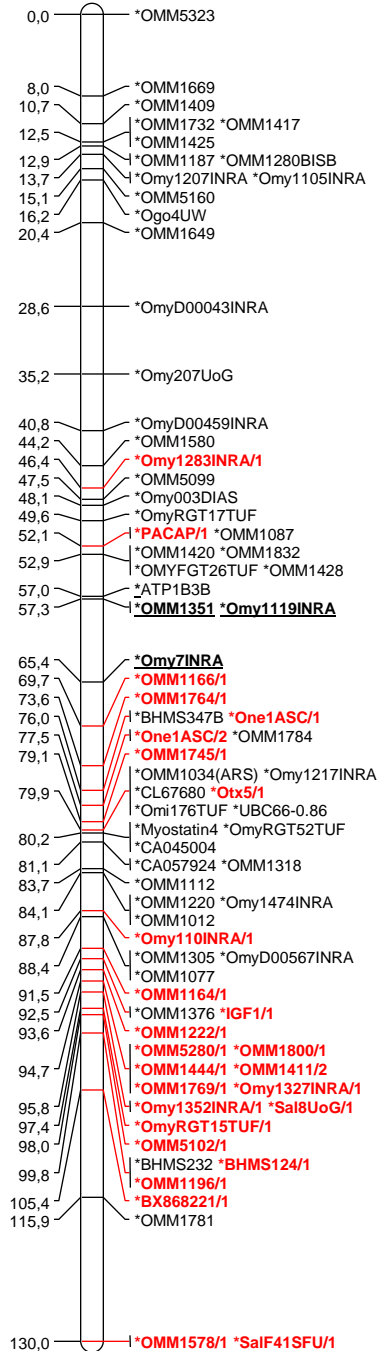

## RT08

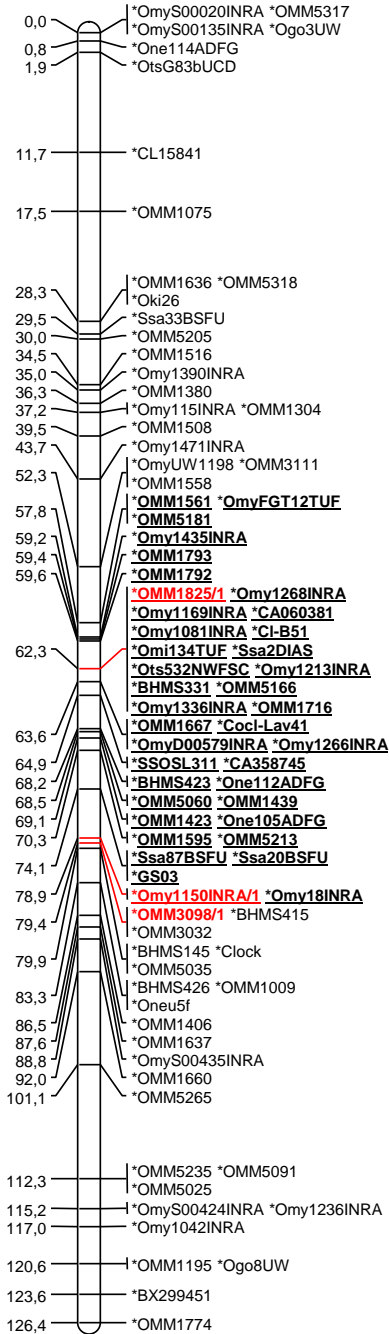

## RT09

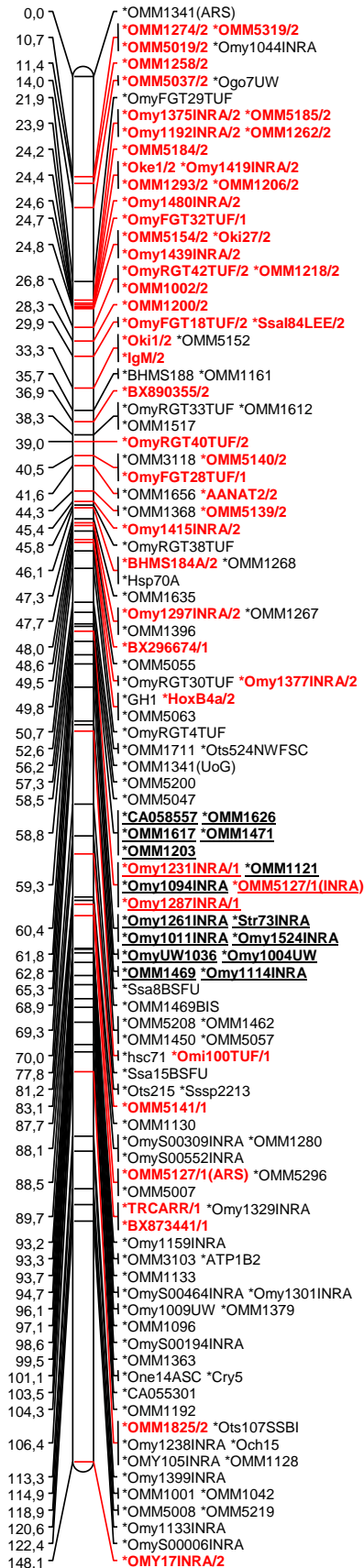

## RT10

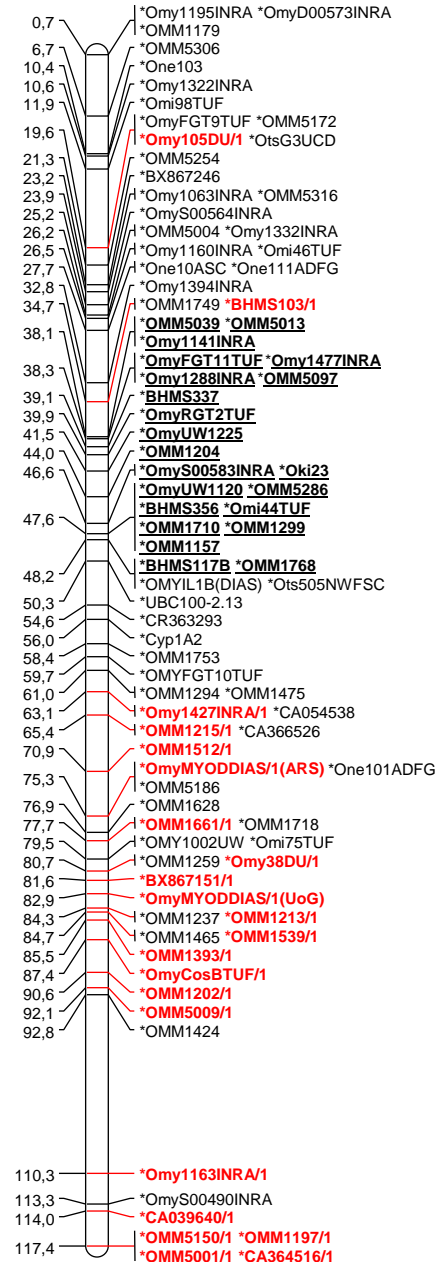

## RT11

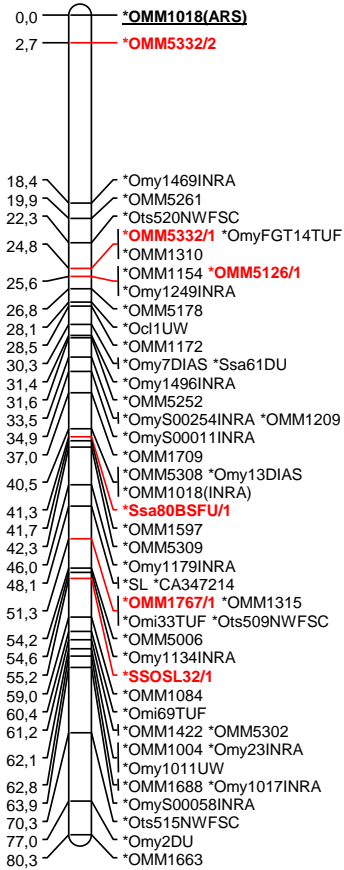

## RT13

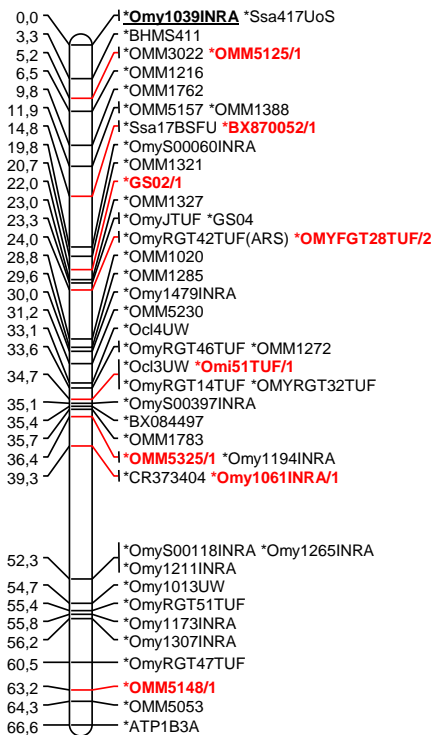

## RT12

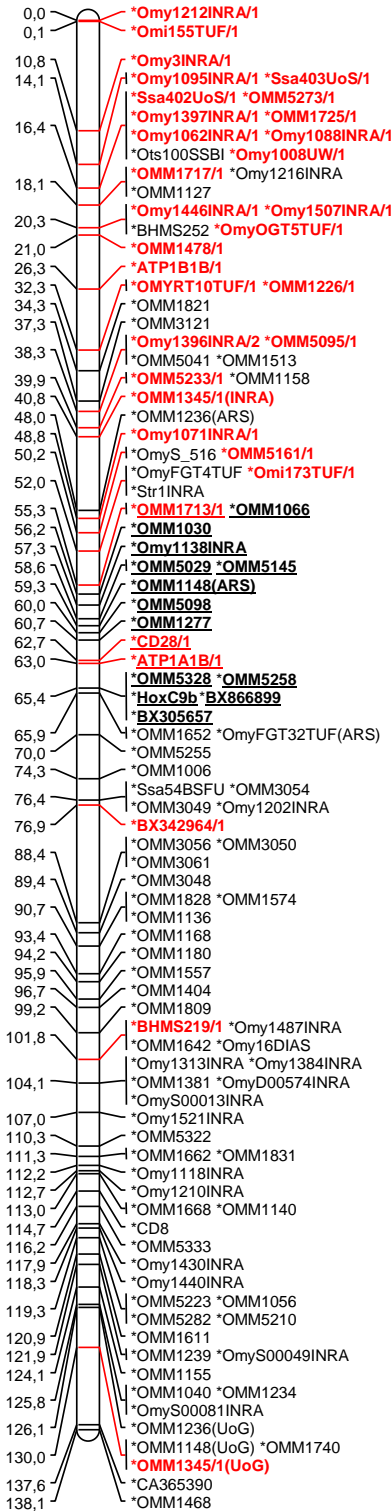

## RT14

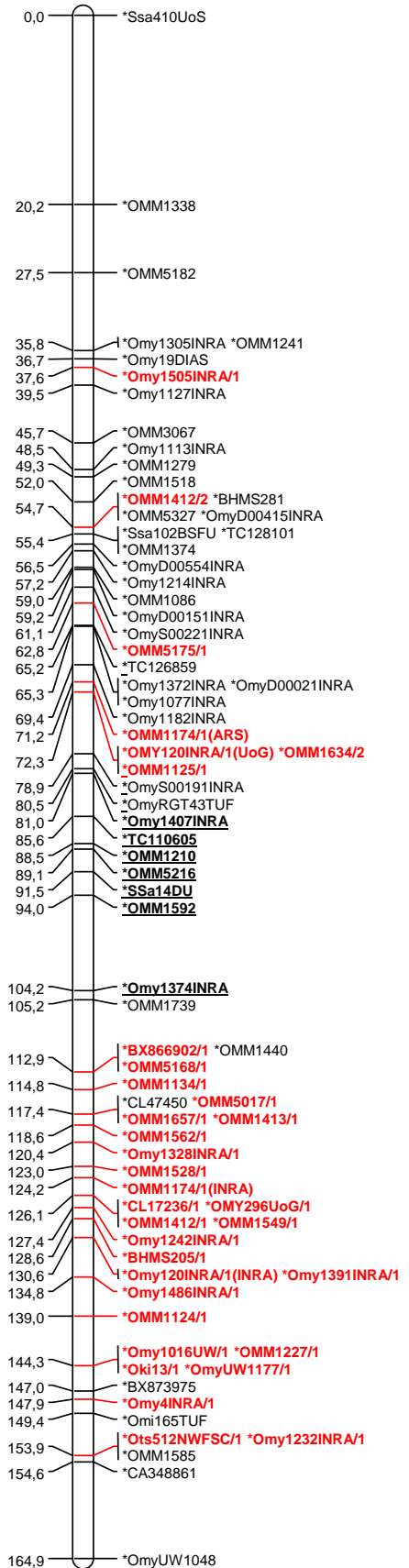

# RT15

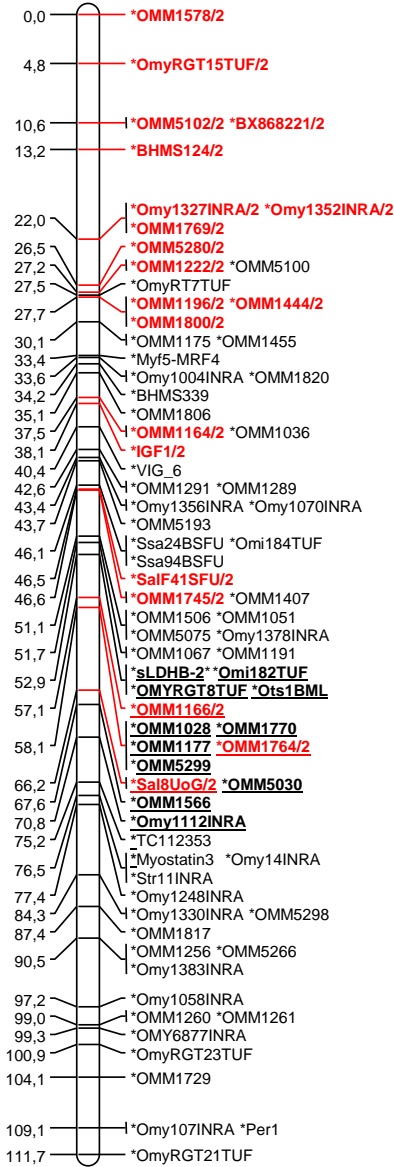

# RT16

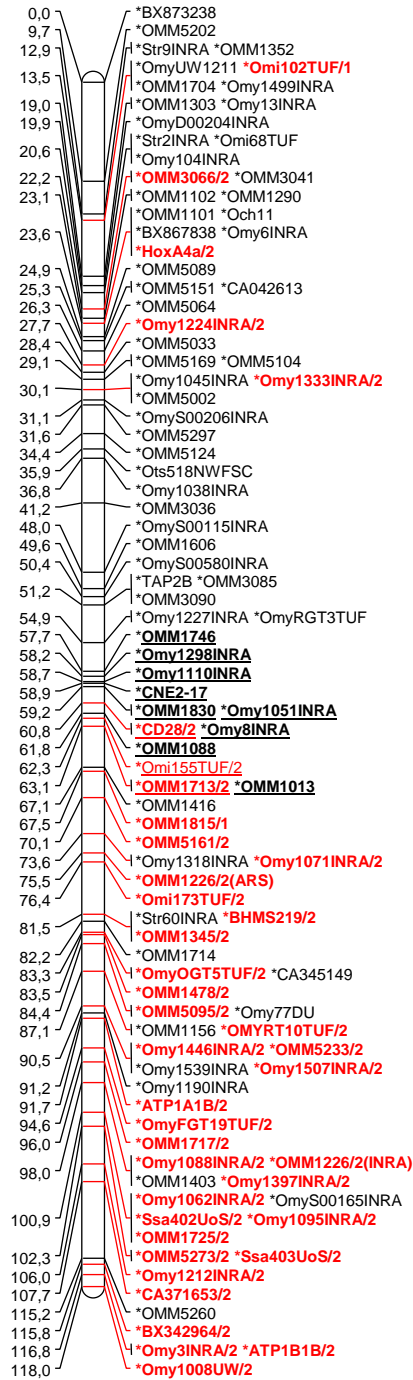

# RT17

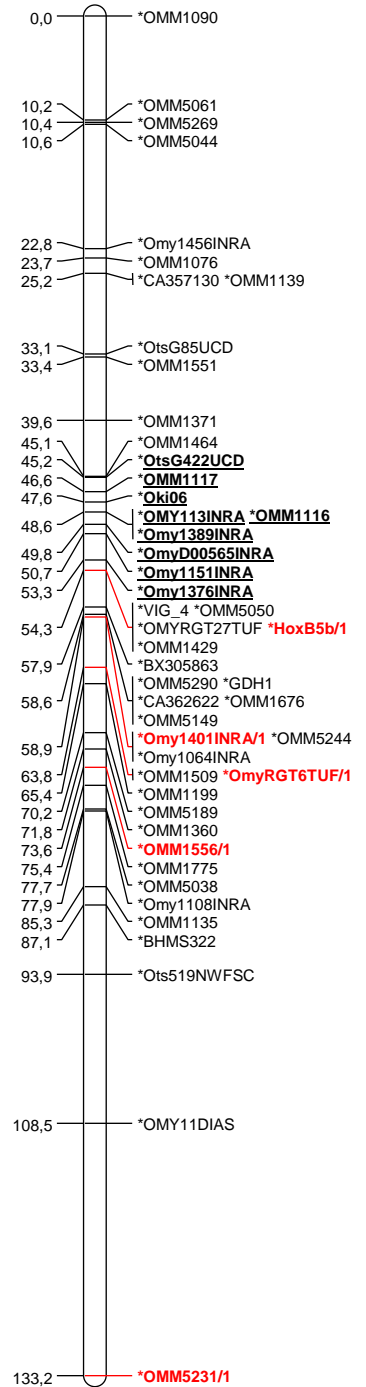

# RT19

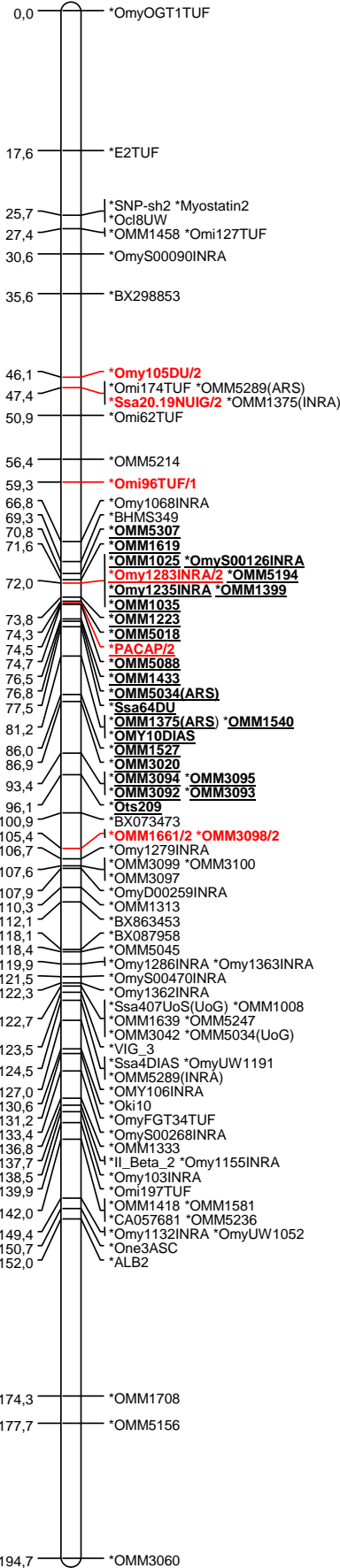

# RT18

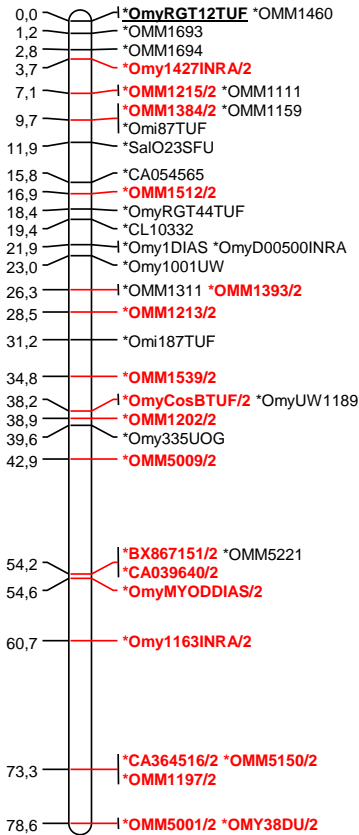

# RT20

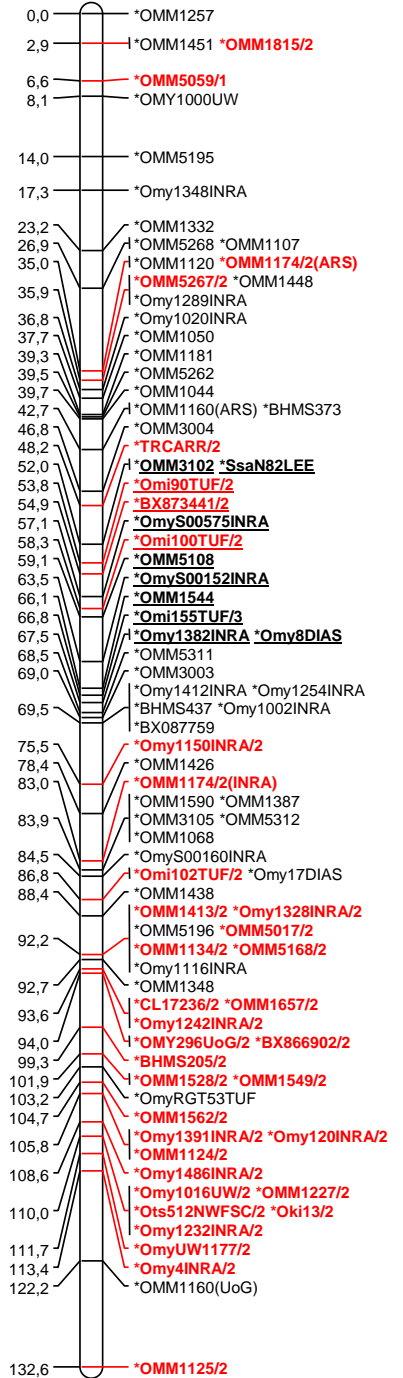

## RT21

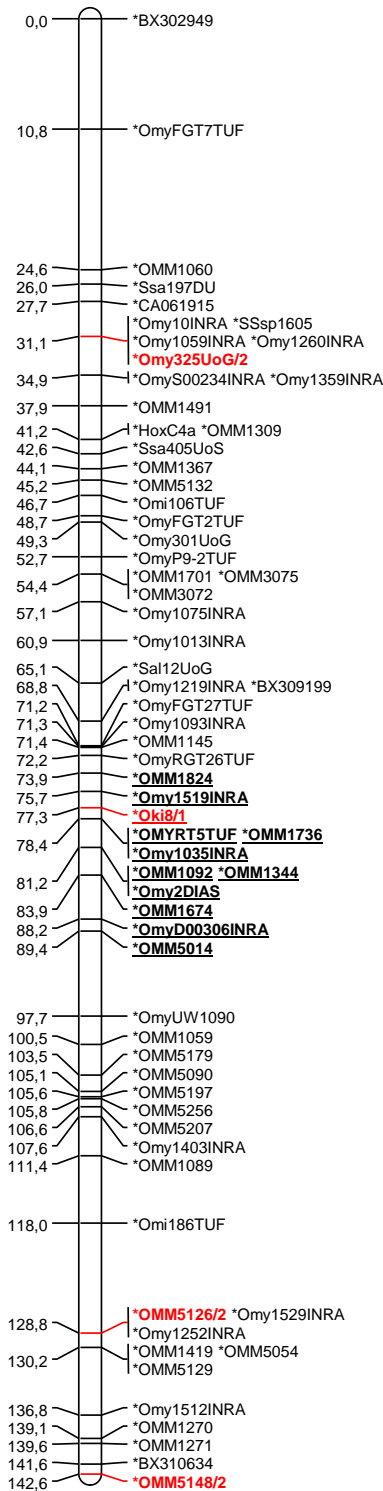

## RT22

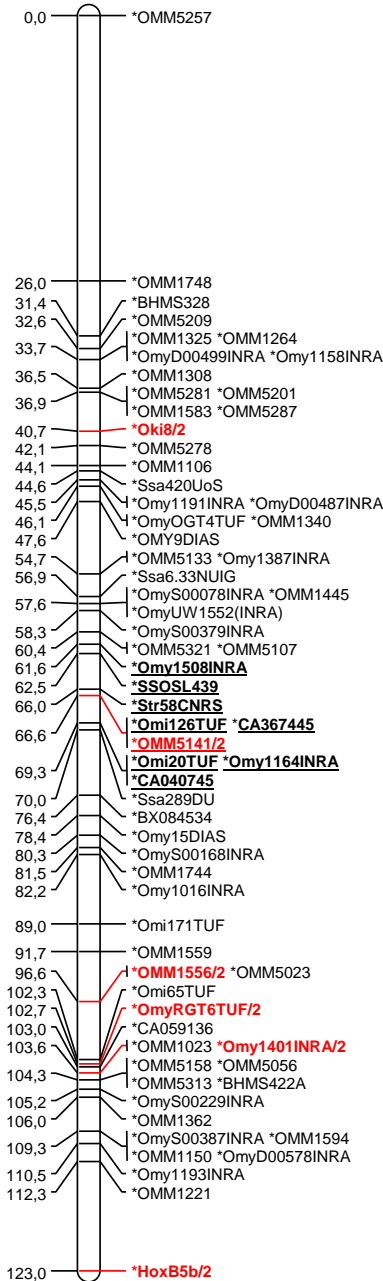

## RT23

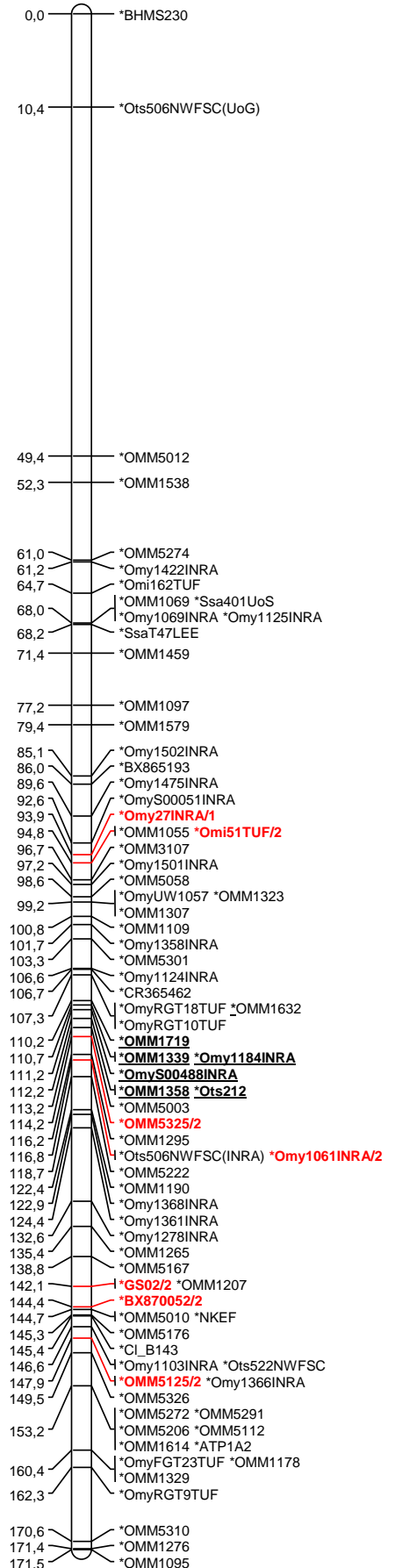

## RT24

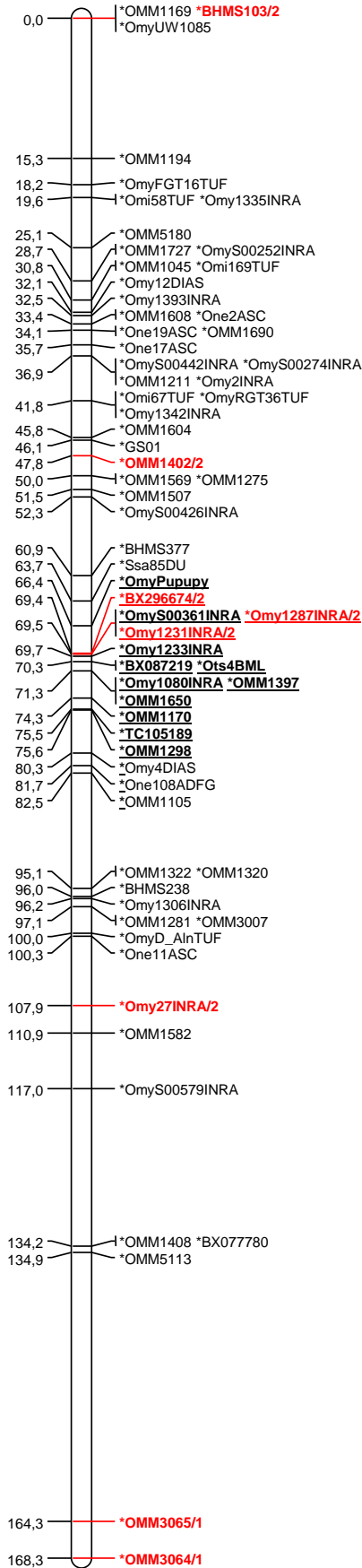

## RT26

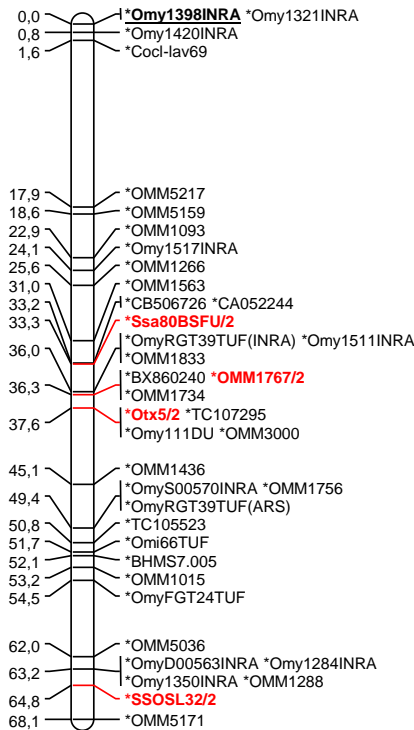

## RT27

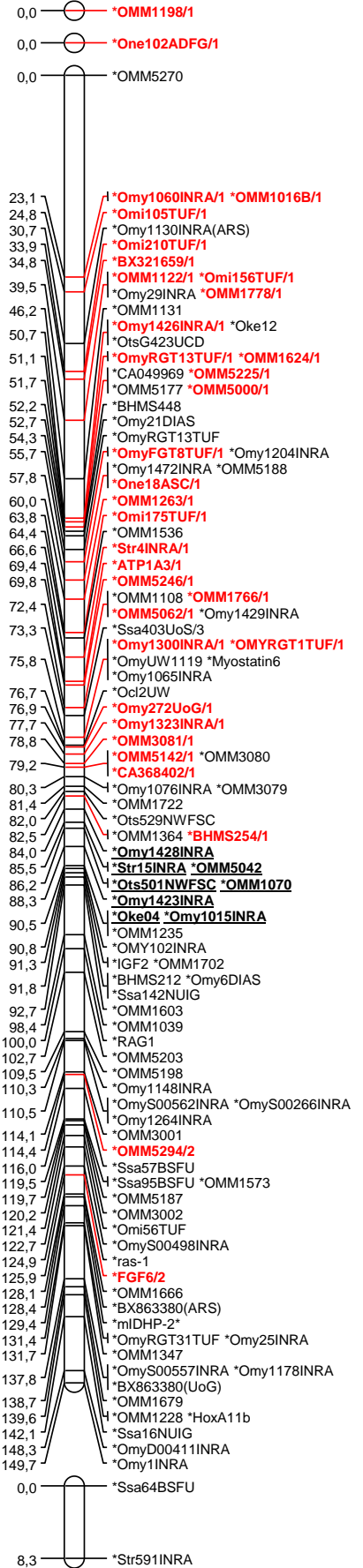

## RT29

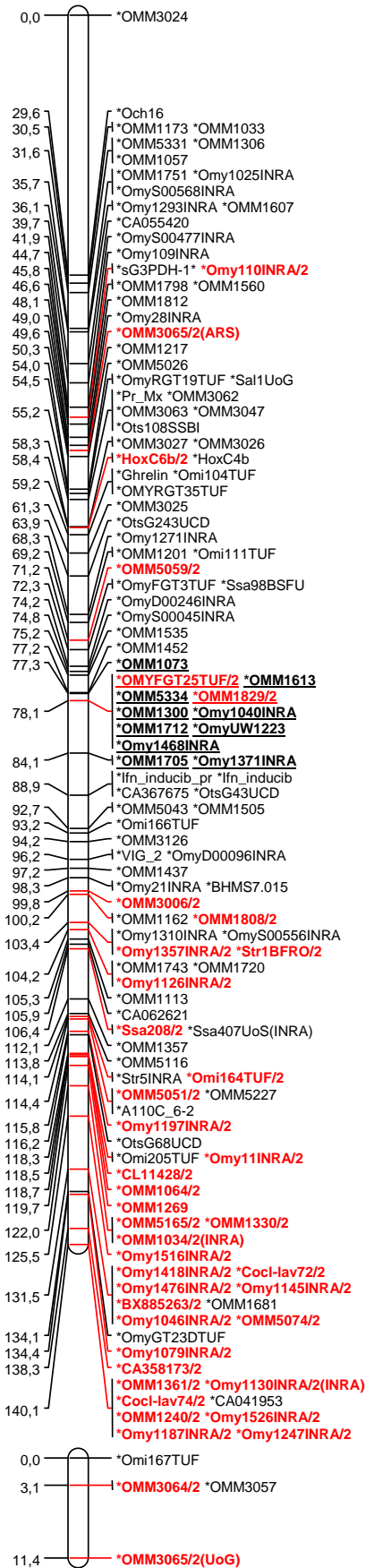

## RT30

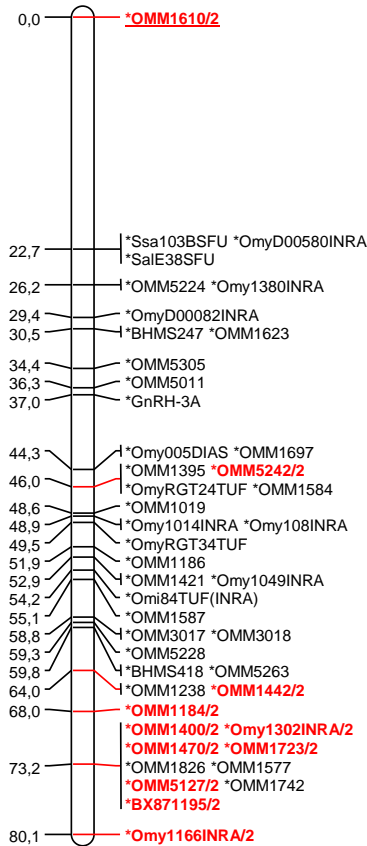

## RT31

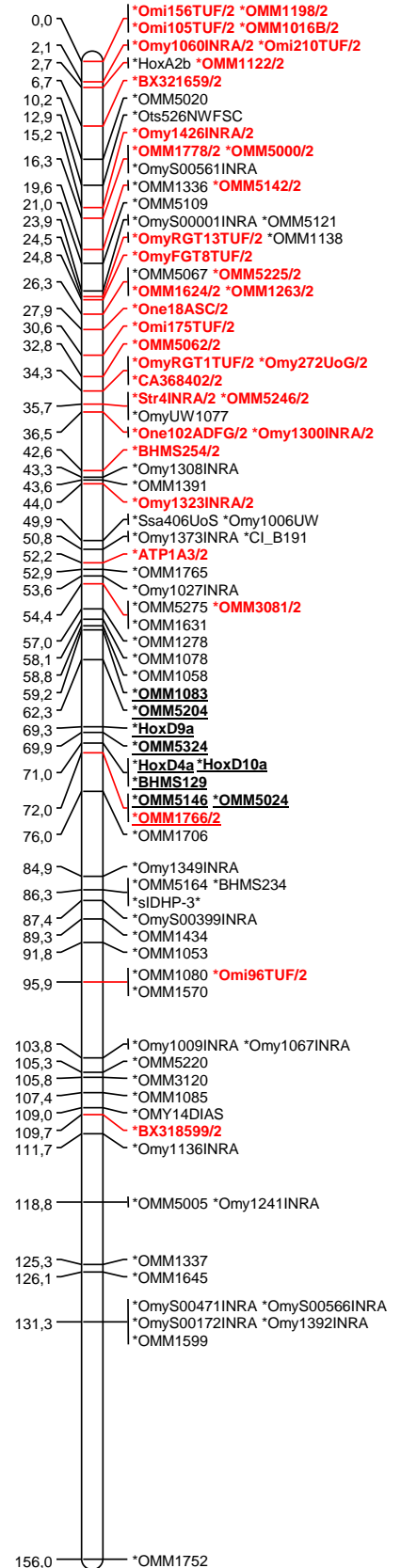

Supplement: Additional file 4 — Graphic representation of the synthetic map (same captions as in Additional file 3). [file 1471-2156-13-15-S4.PDF]

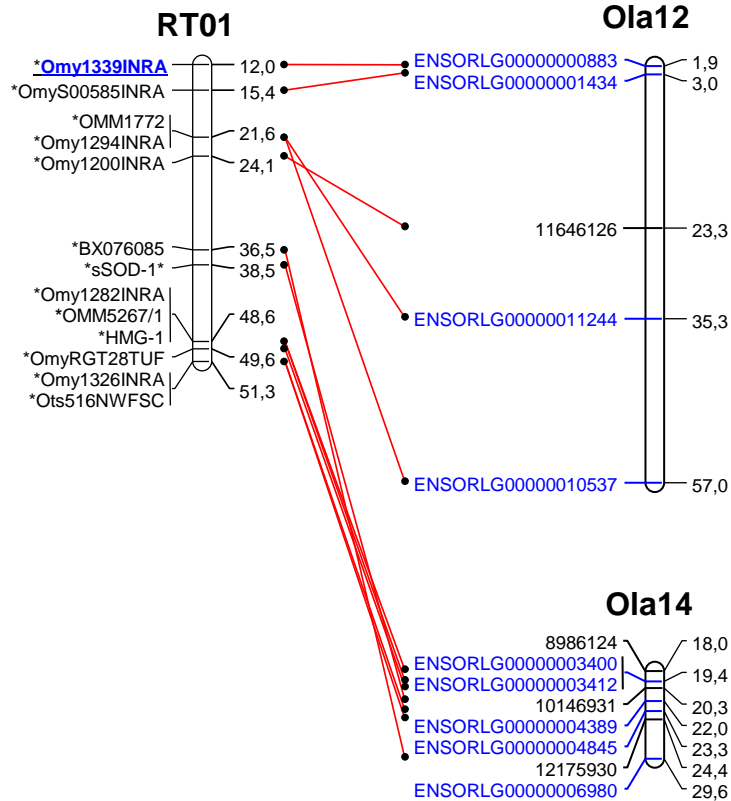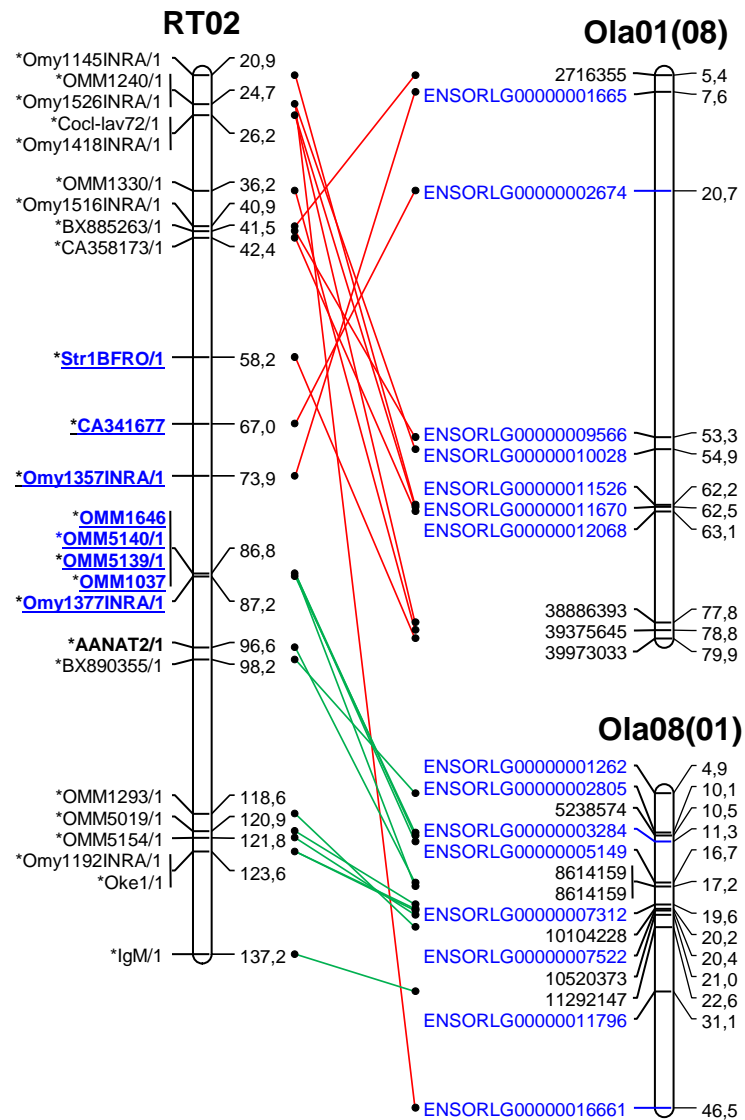

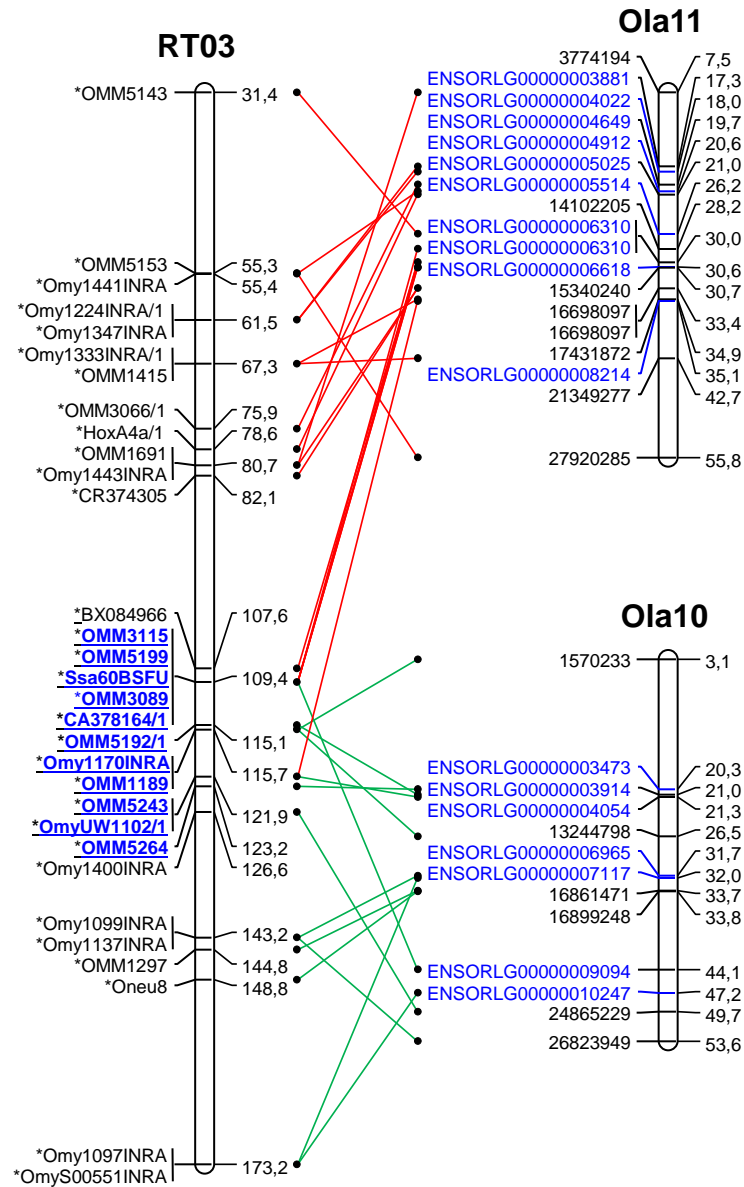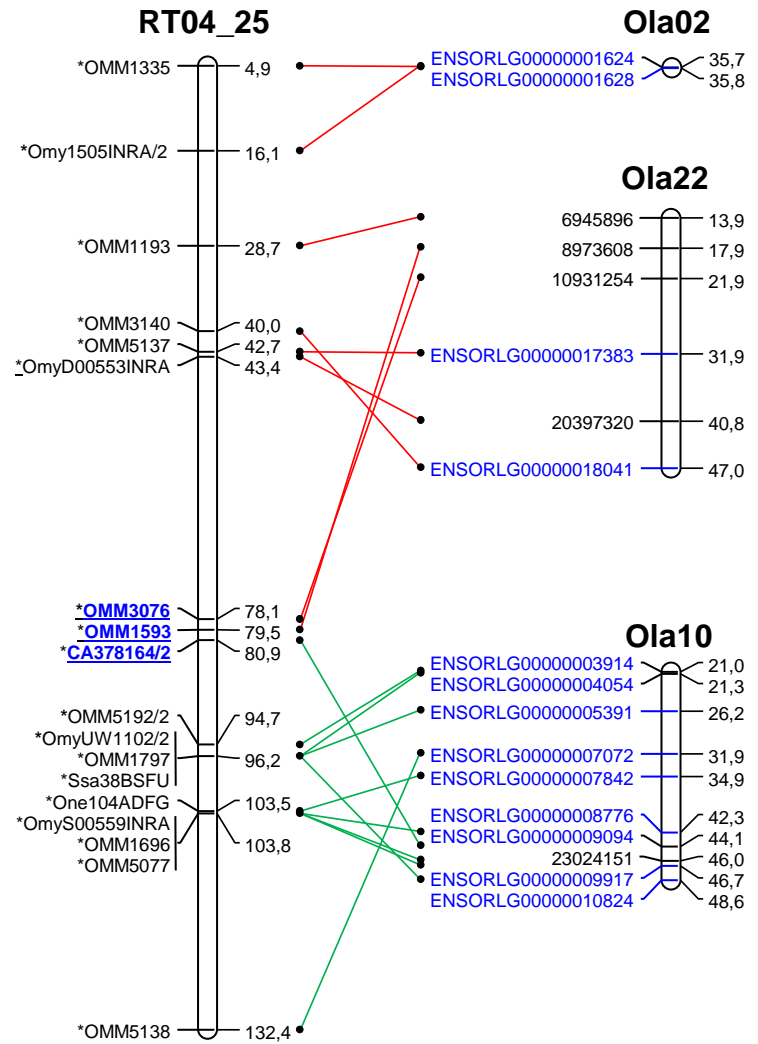

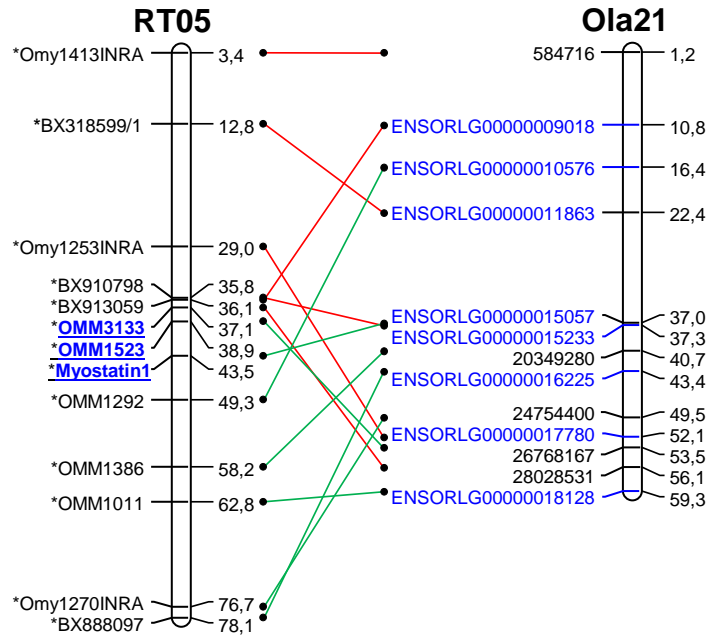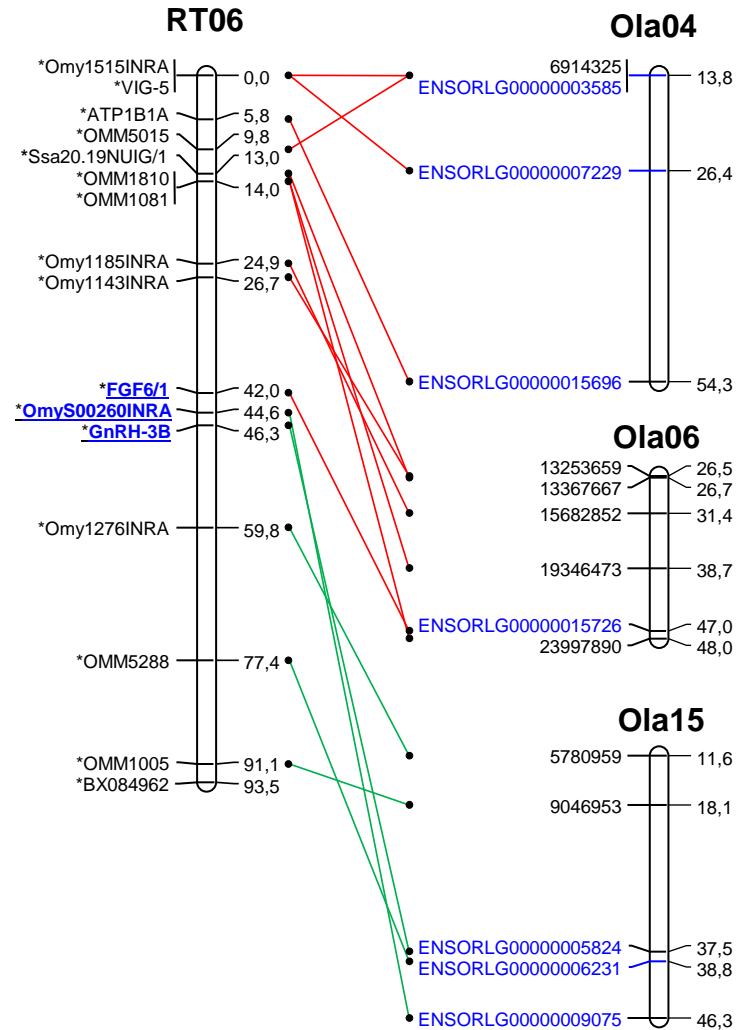

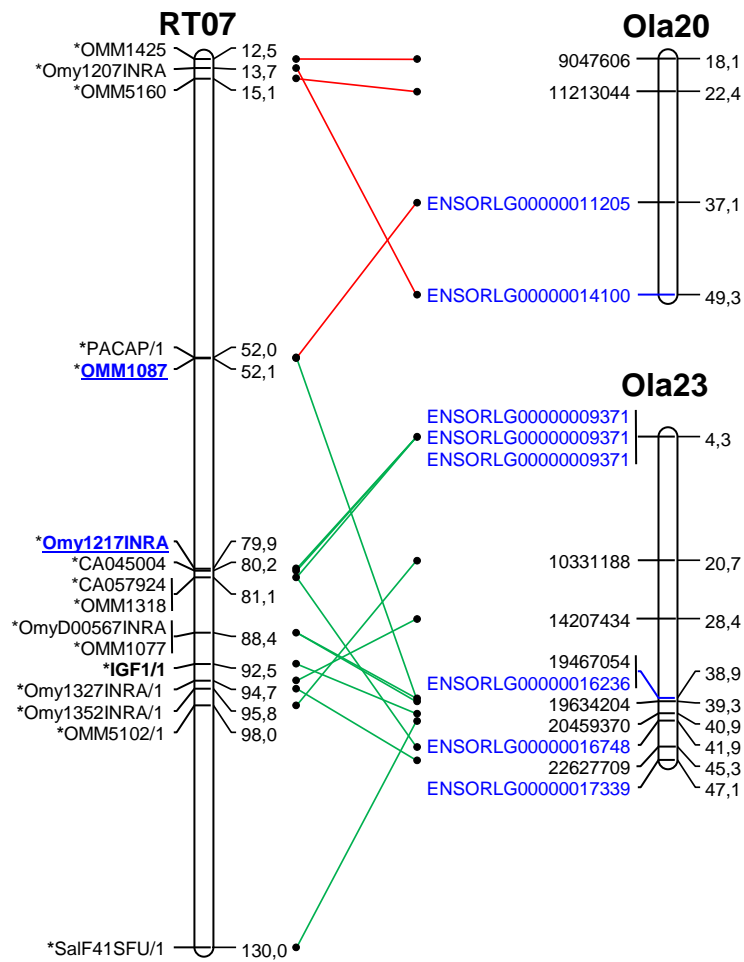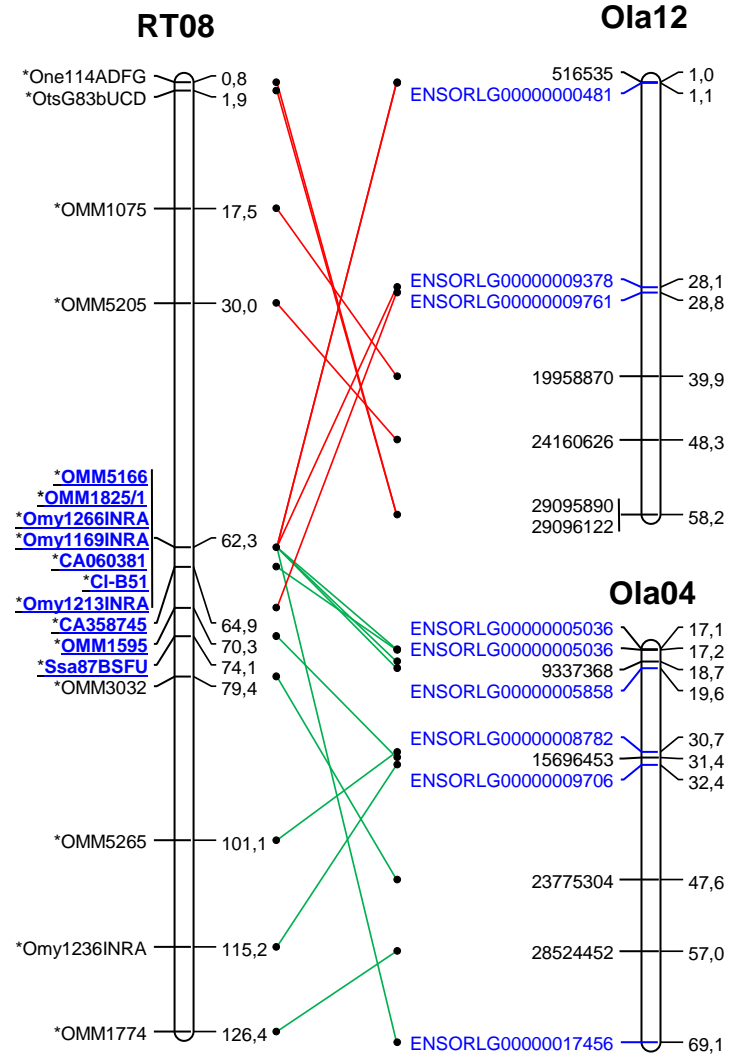

## Ola08

## RT09

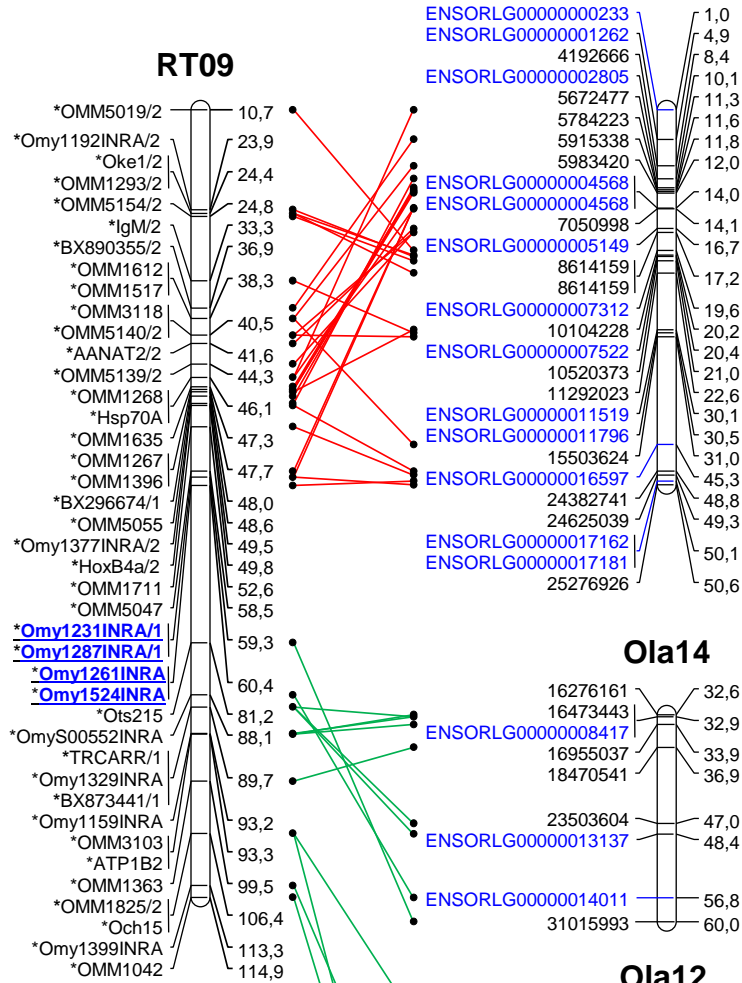

## Ola14

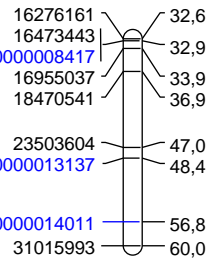

## Ola12

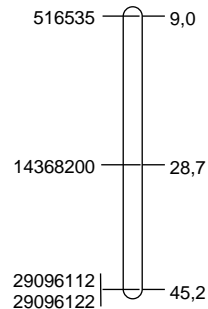

## RT10

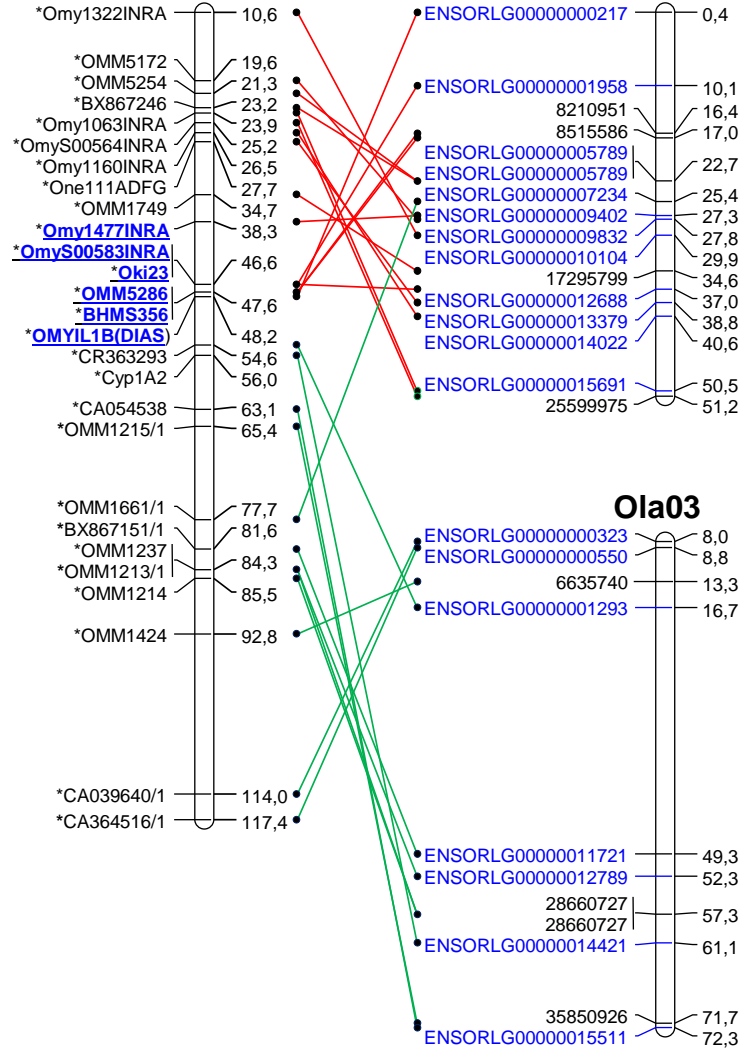

## Ola03

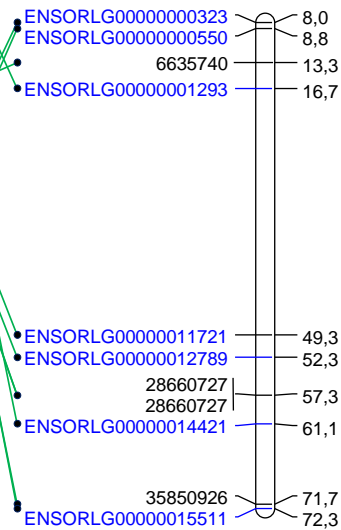

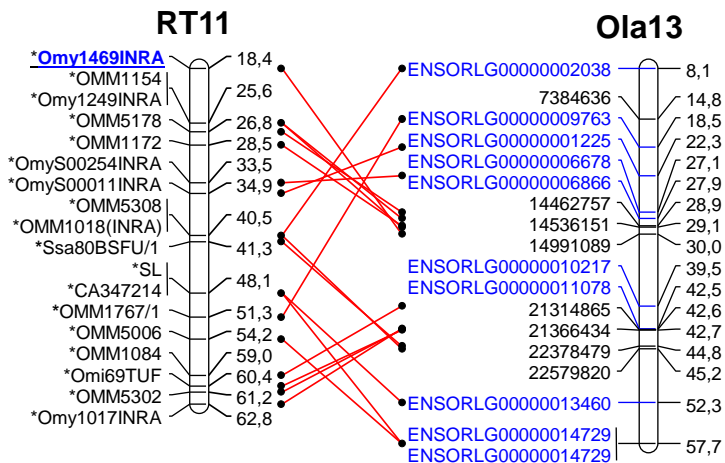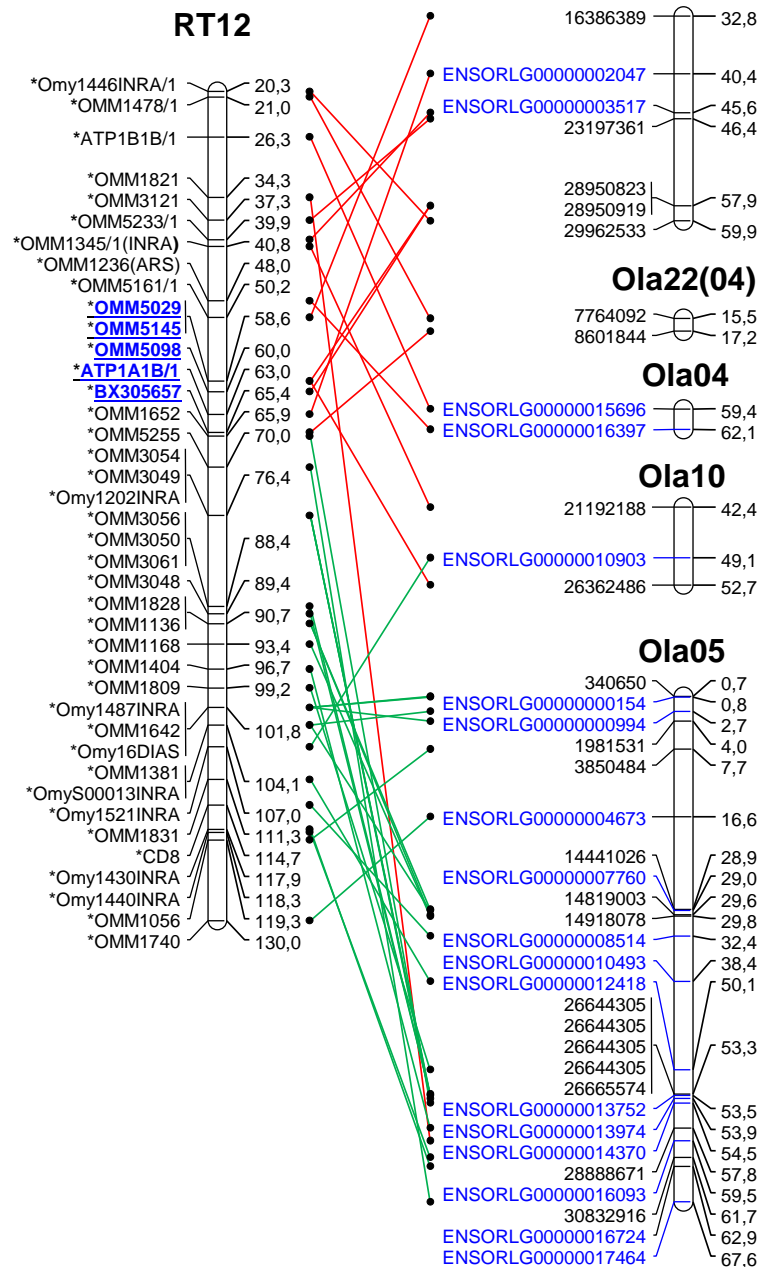

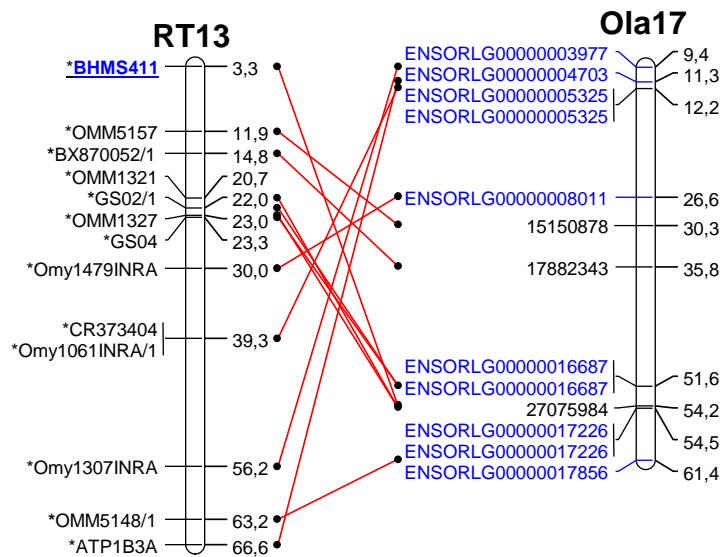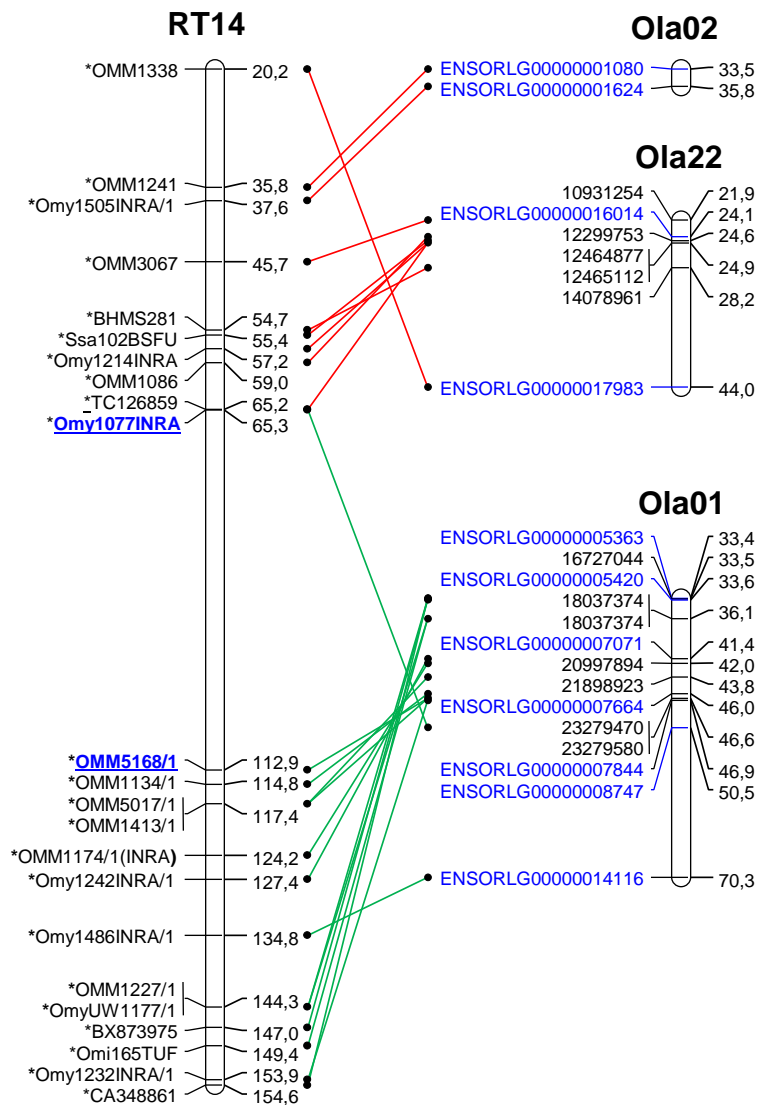

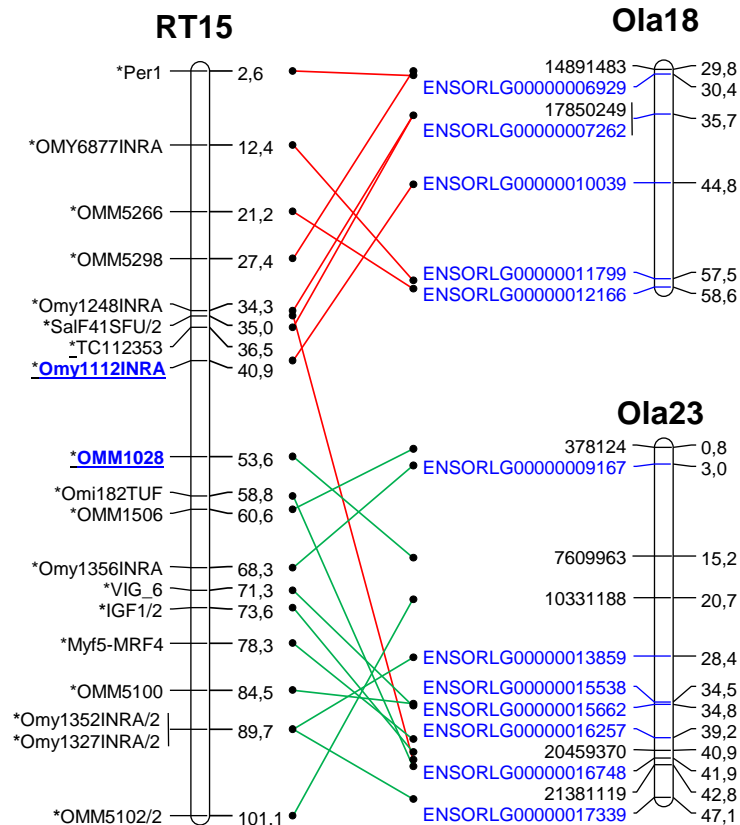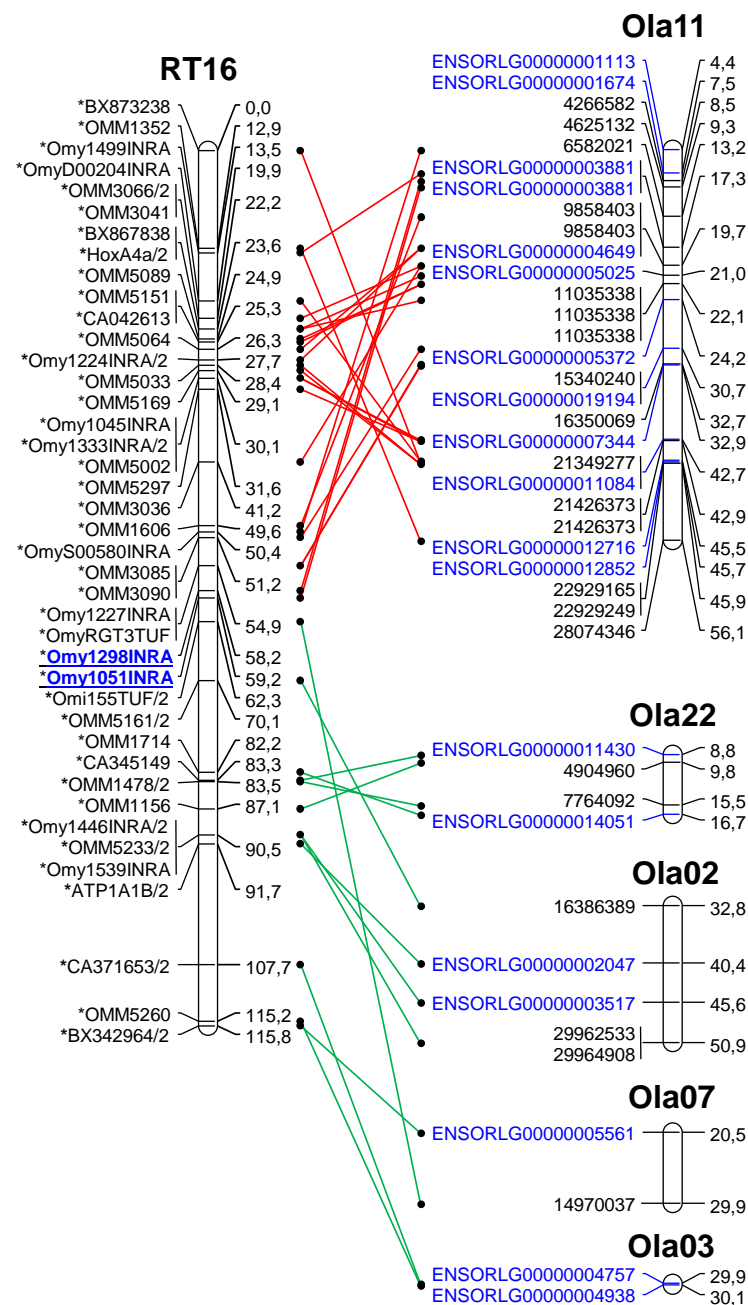

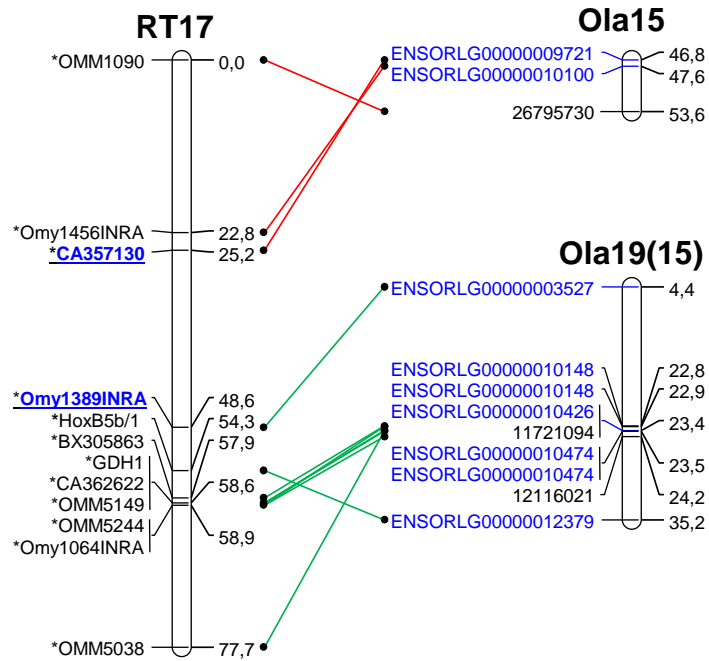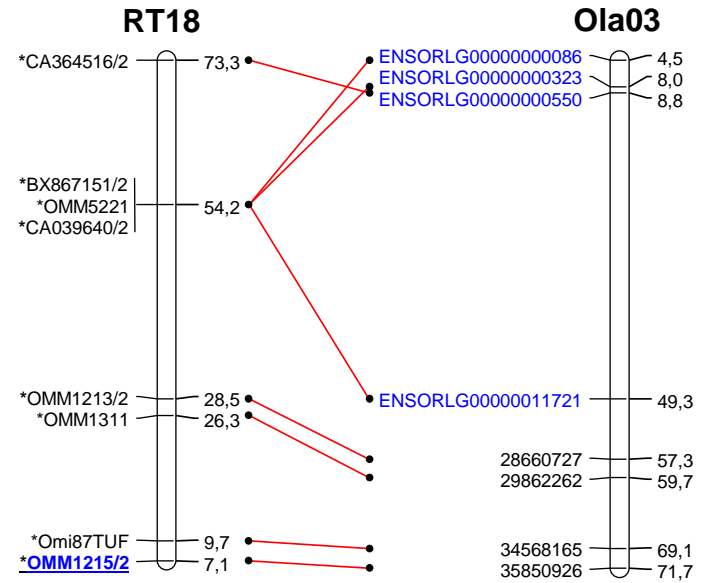

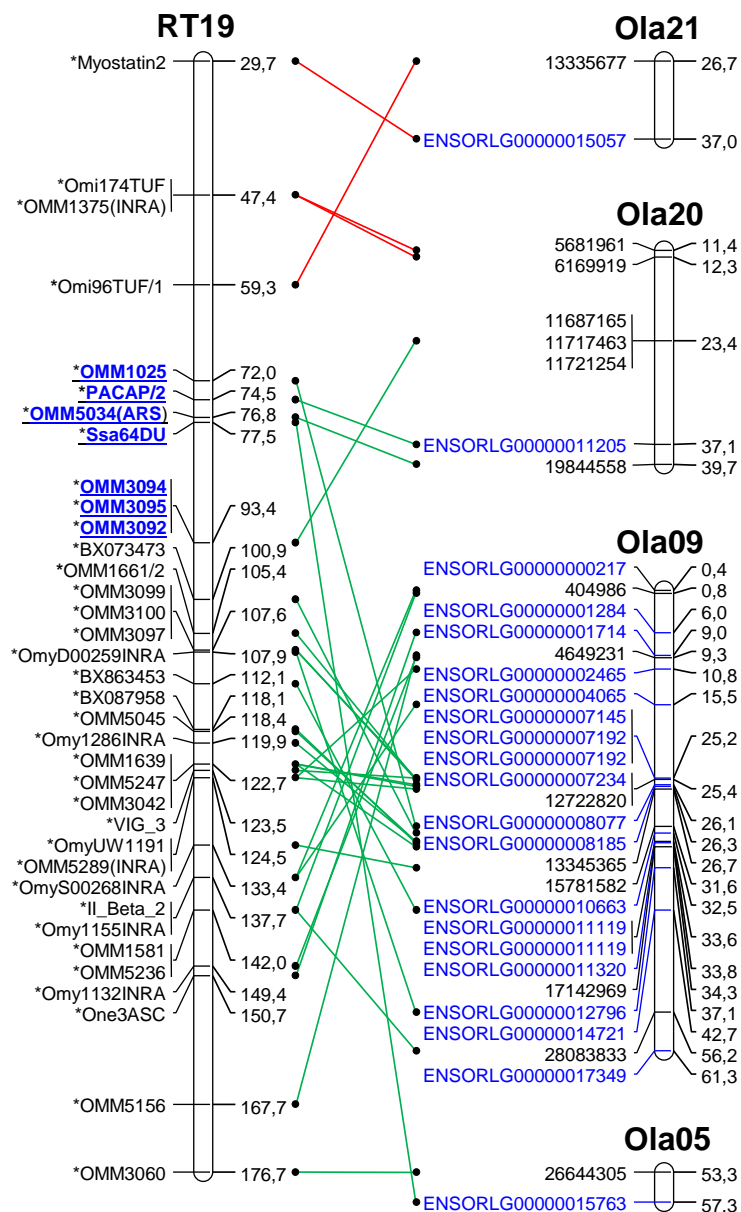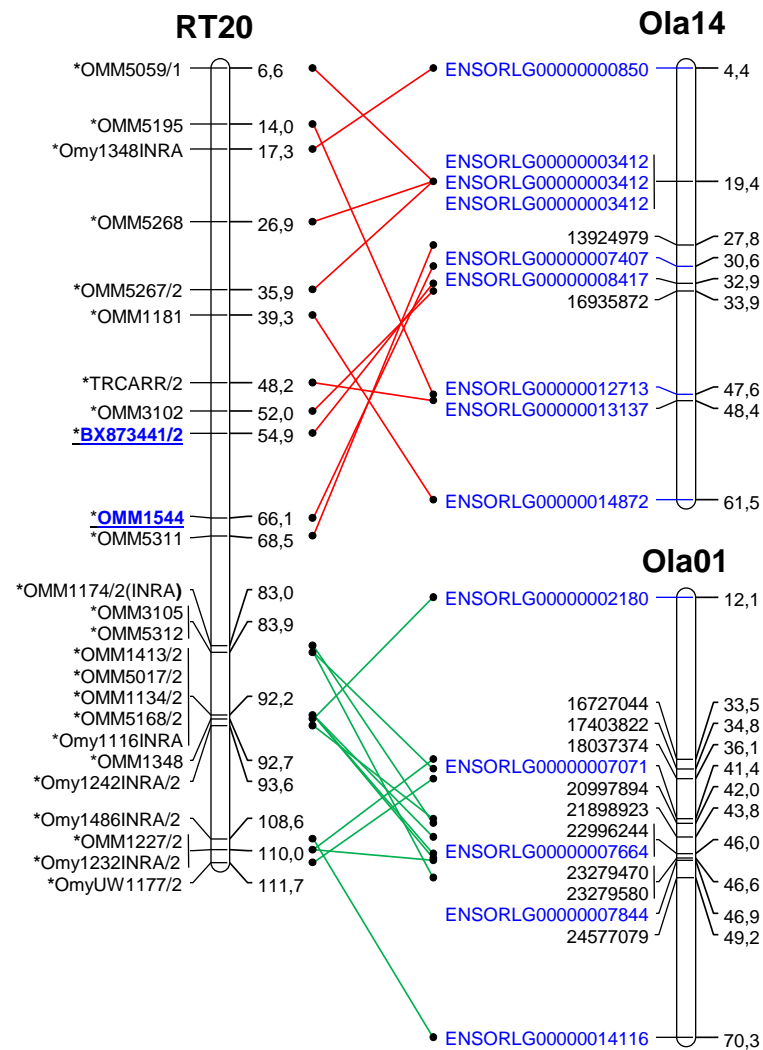

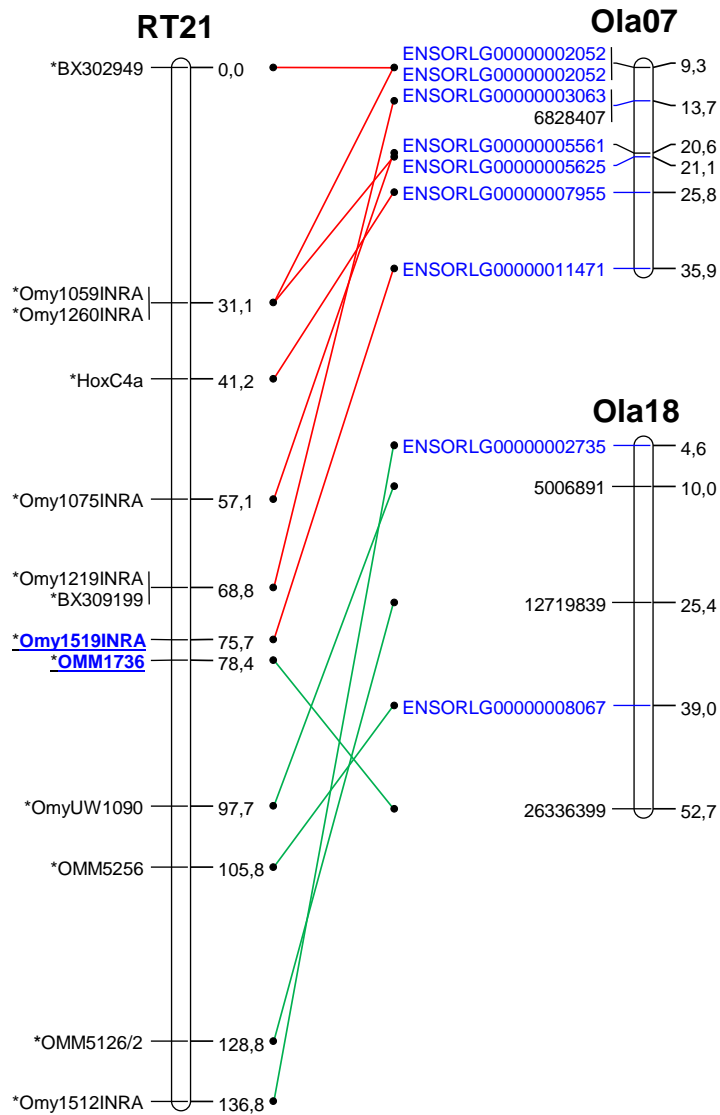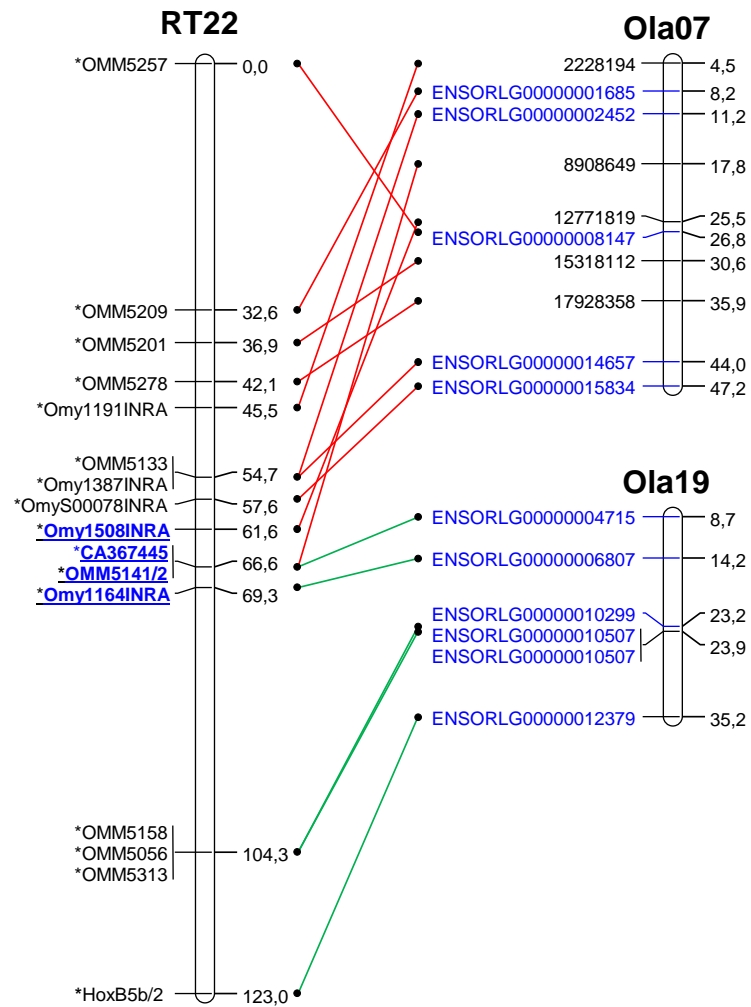

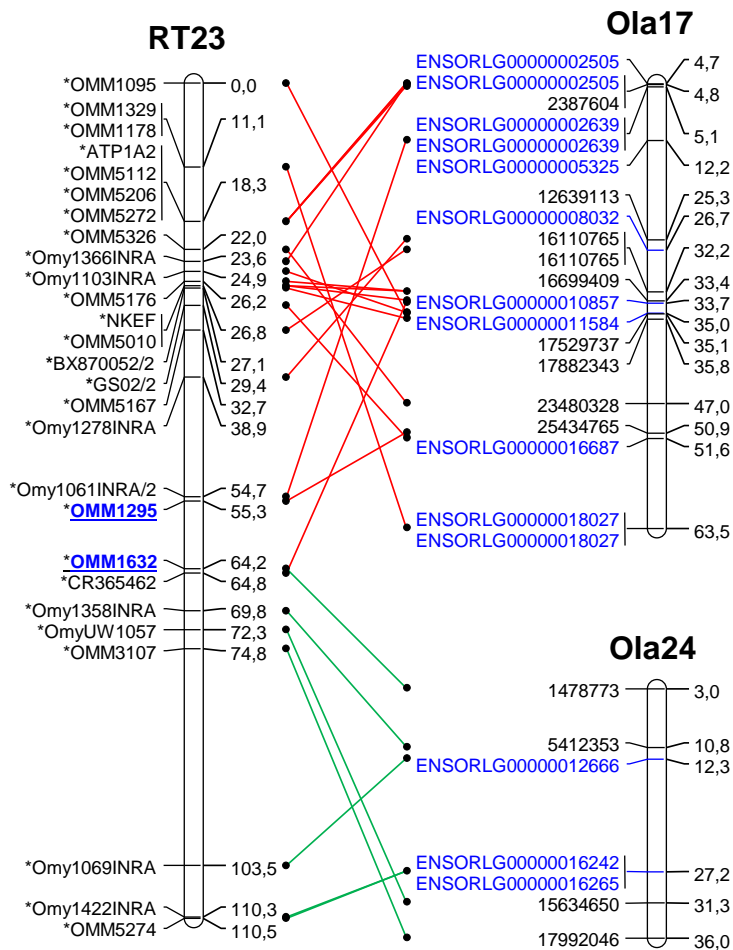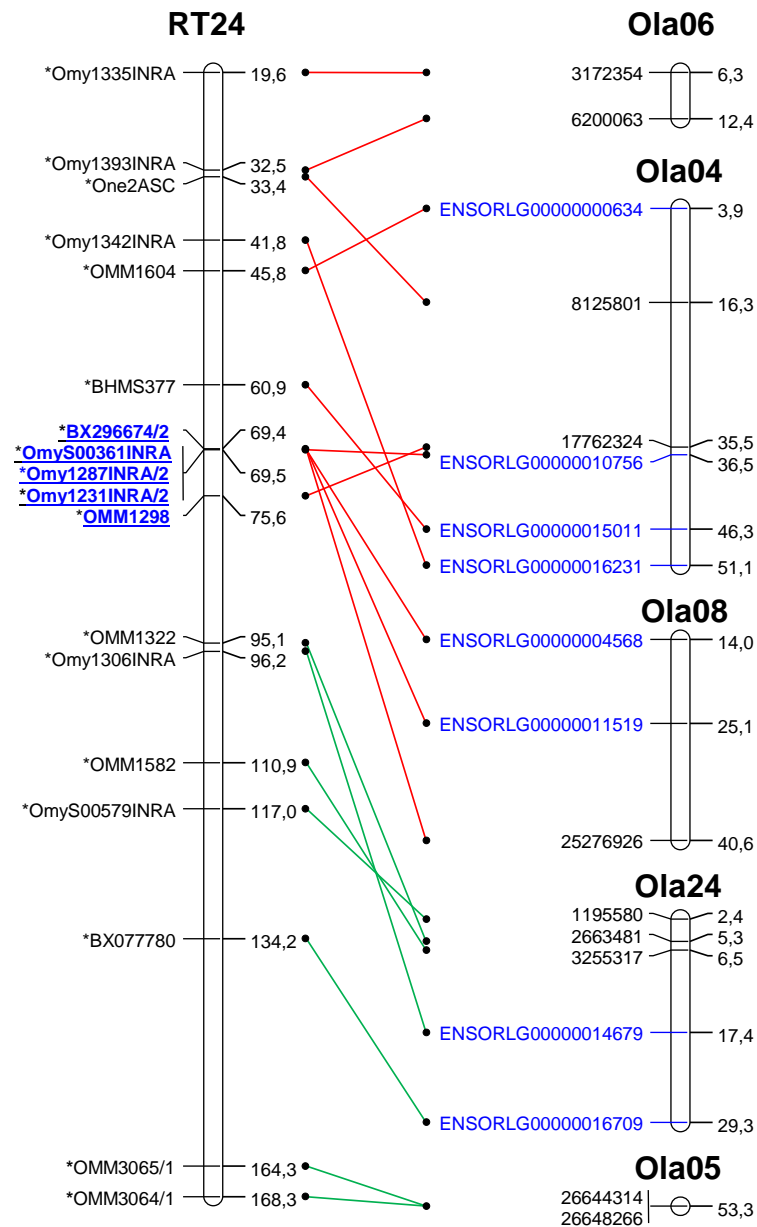

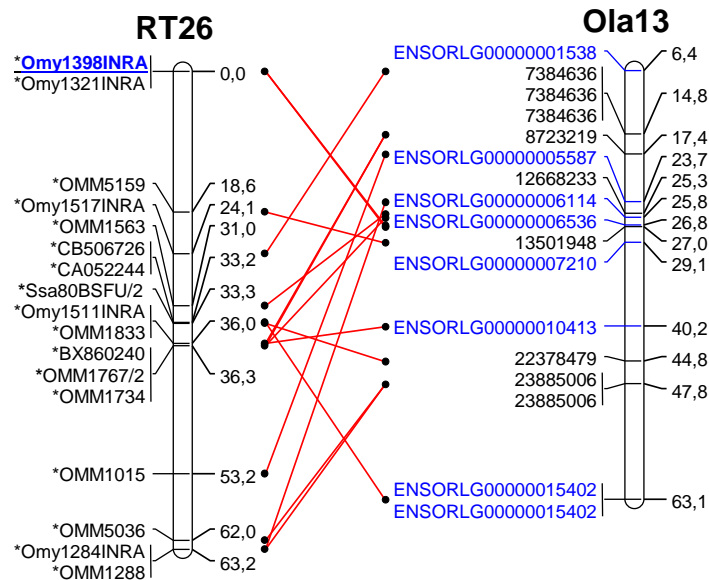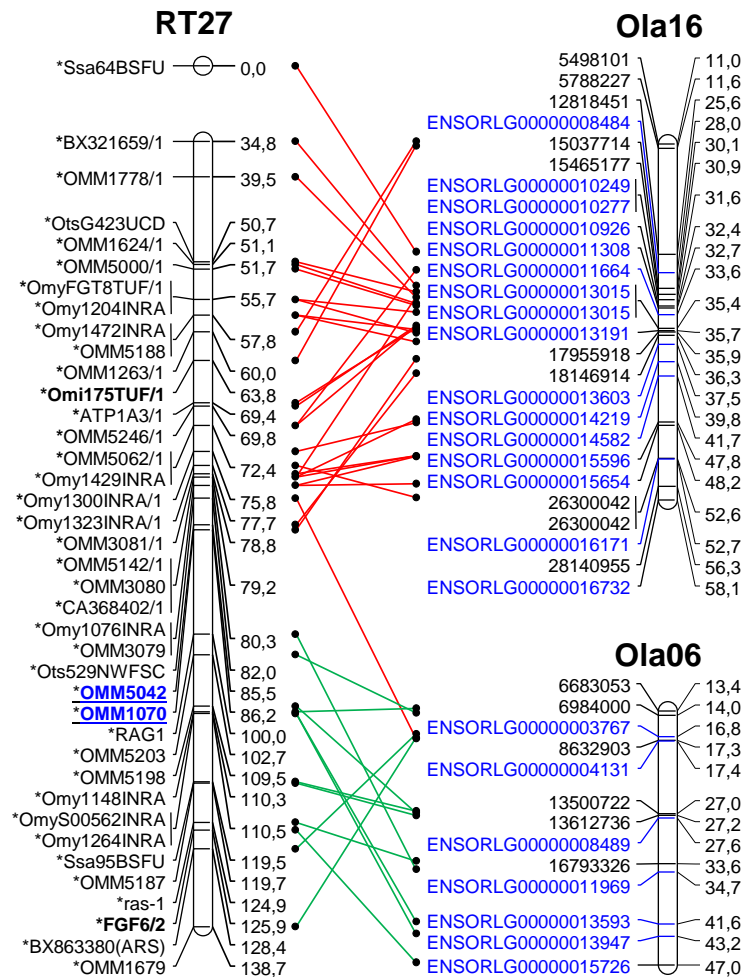

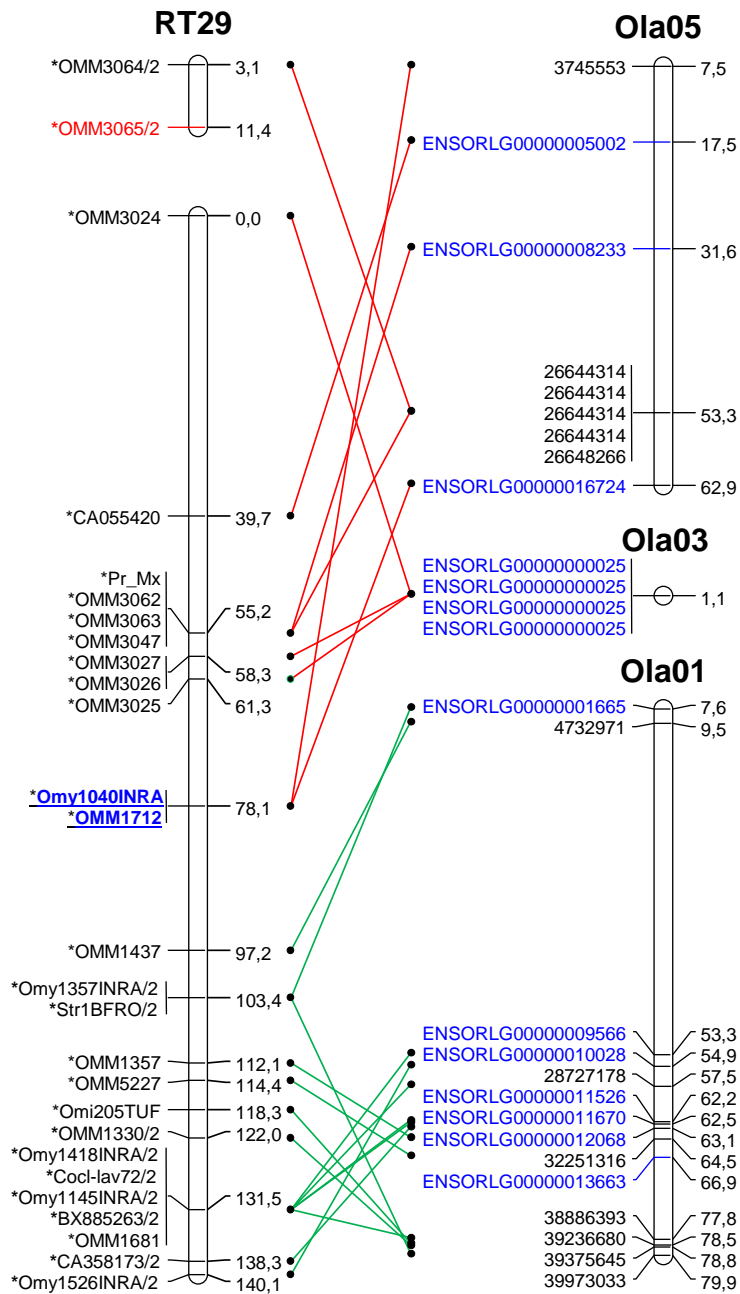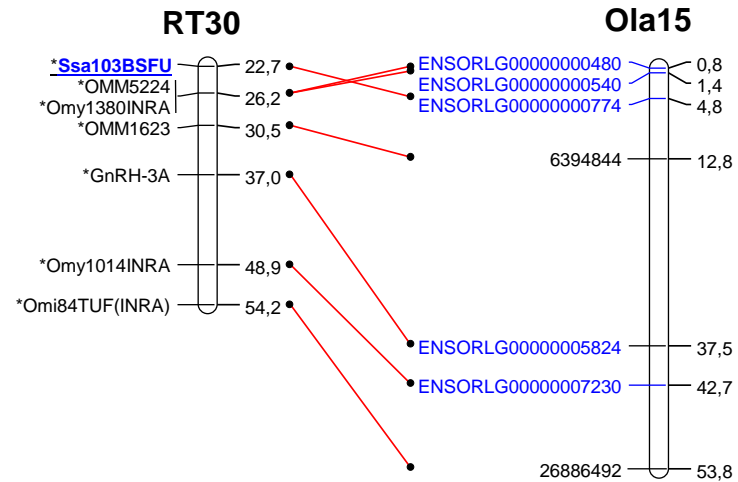

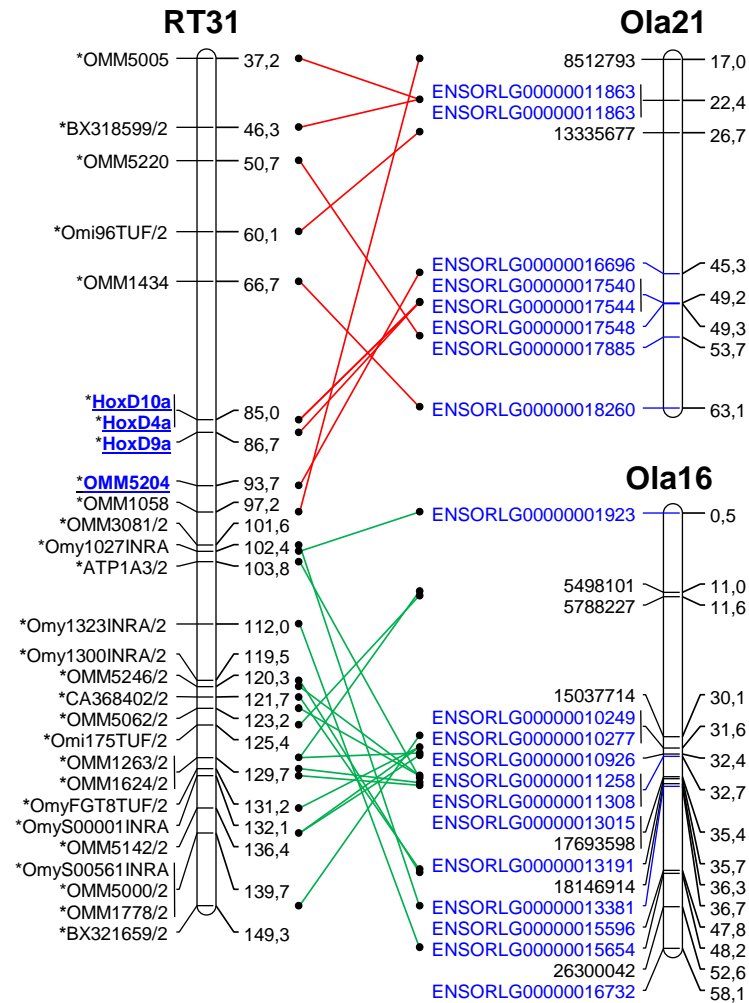

Supplement: Additional file 7 — Map alignments between chromosomes of rainbow trout and zebrafish; underlined blue bold type marker names approximately localize centromeric regions; green and red lines distinguish between the two arms in acrocentric rainbow trout chromosomes; homologous marker positions in model species chromosomes are identified by Gene ID and sequence start position when blastx hits and blastn hits are used respectively. [file 1471-2156-13-15-S7.PDF]

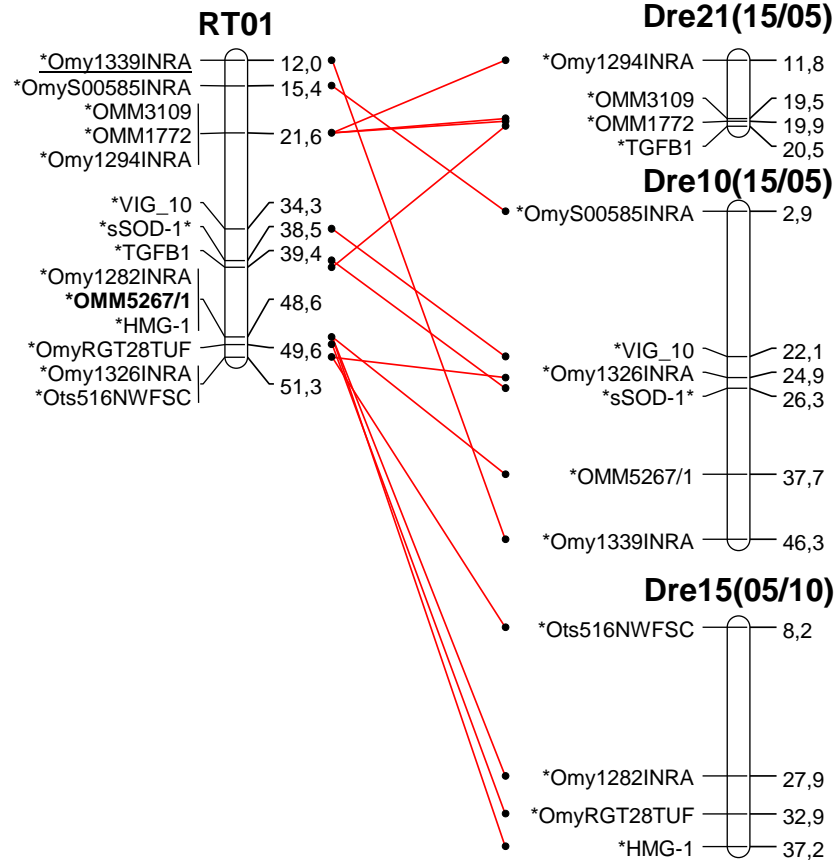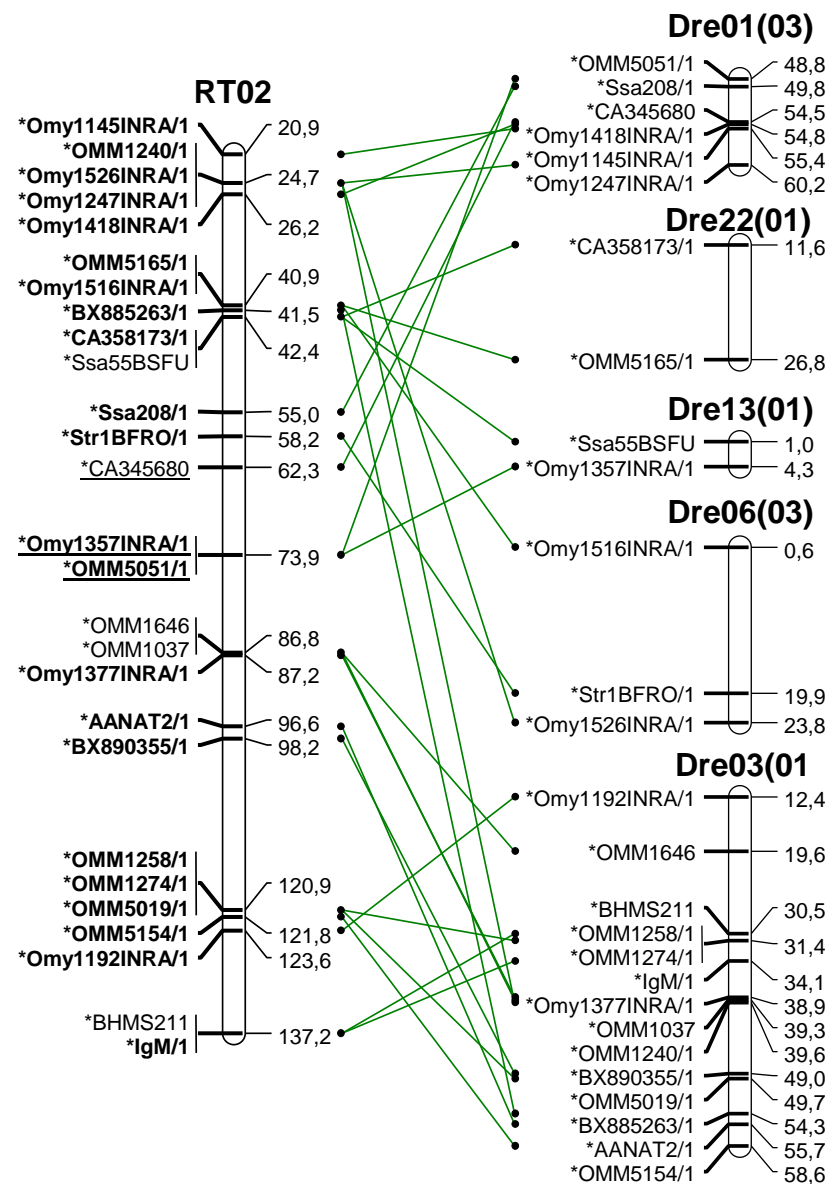

## RT03

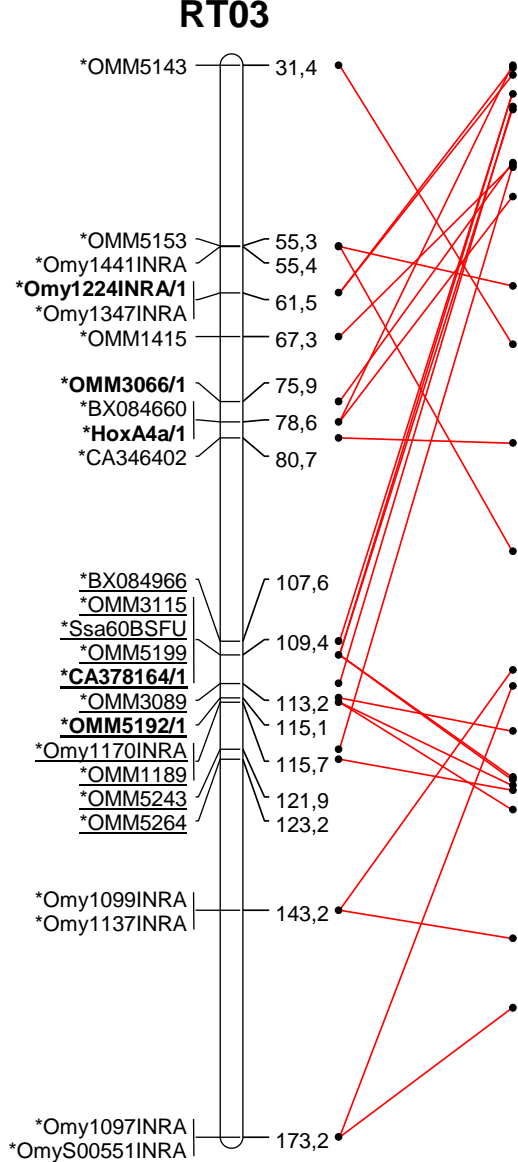

## Dre19

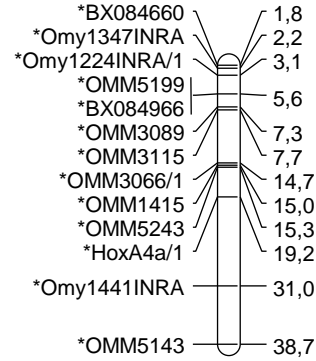

## Dre16(19)

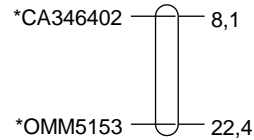

## Dre14

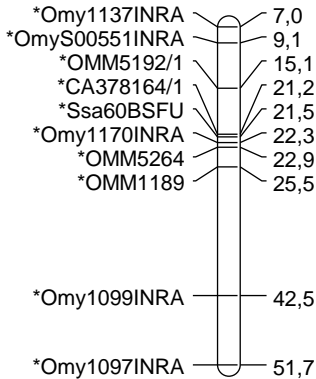

## RT04\_25

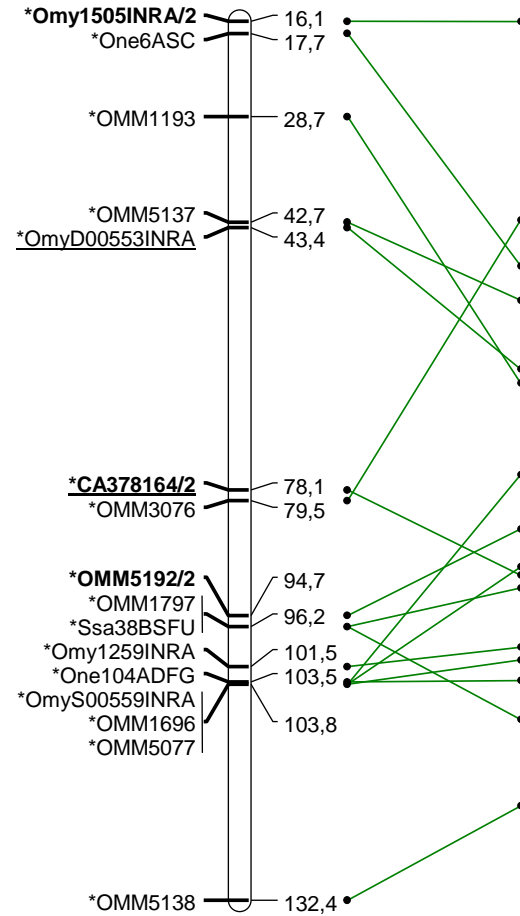

## Dre17

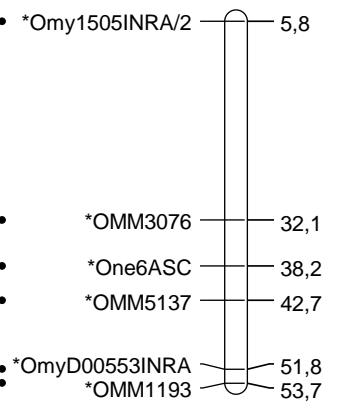

## Dre14

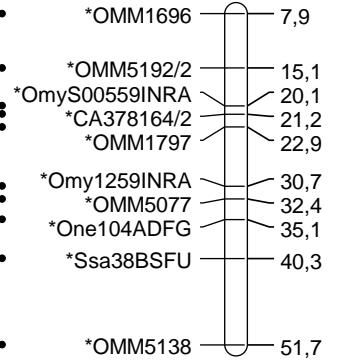

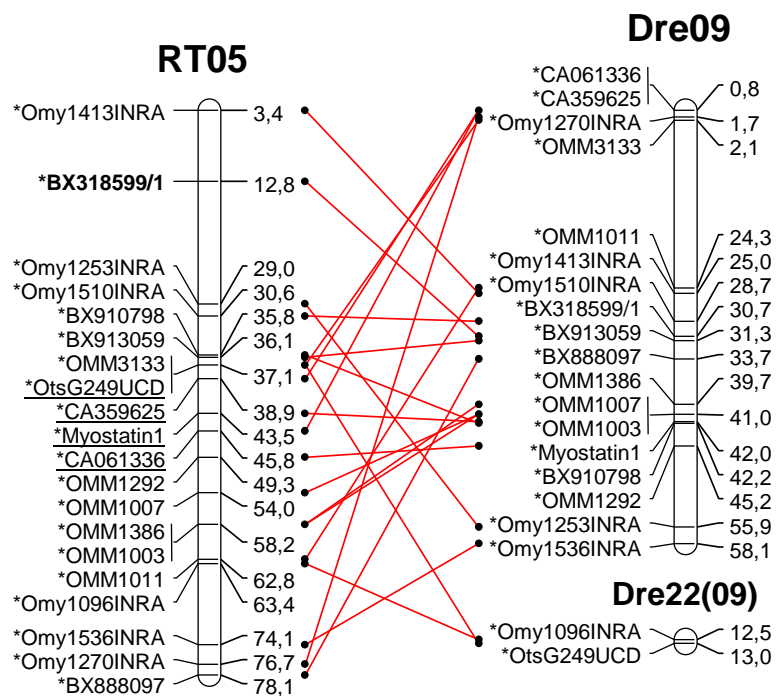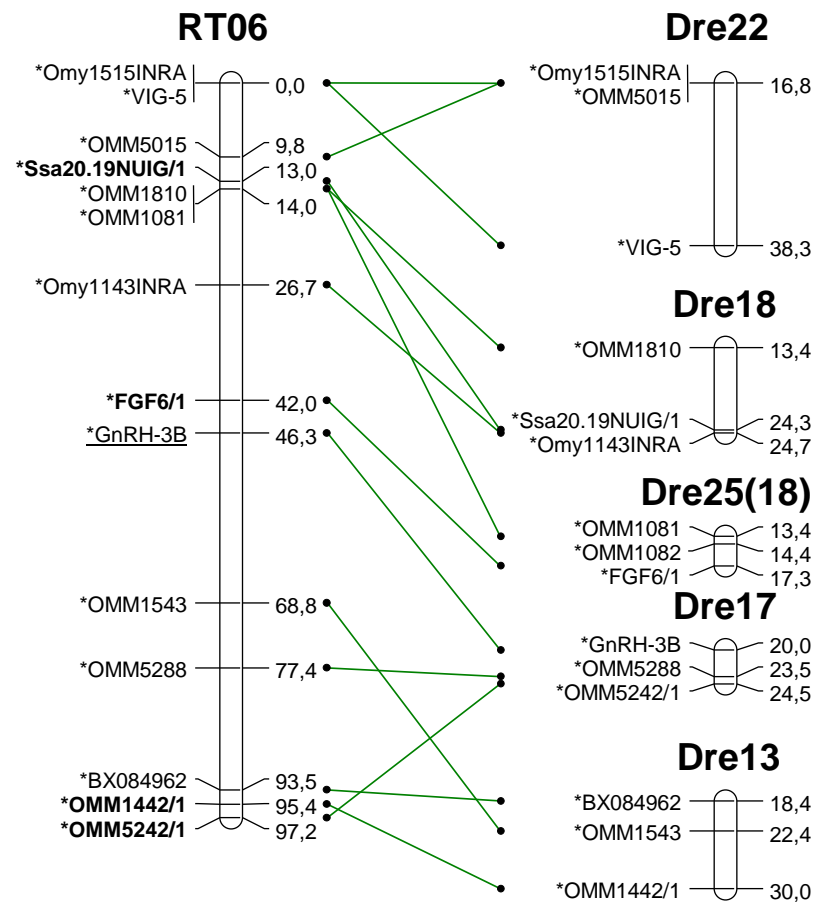

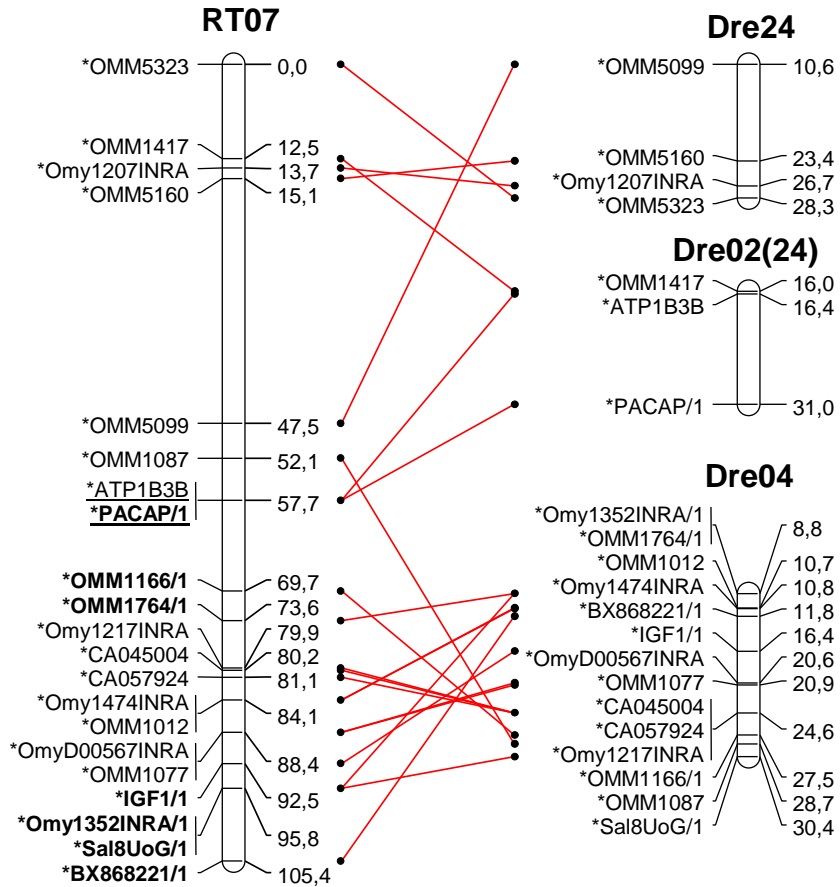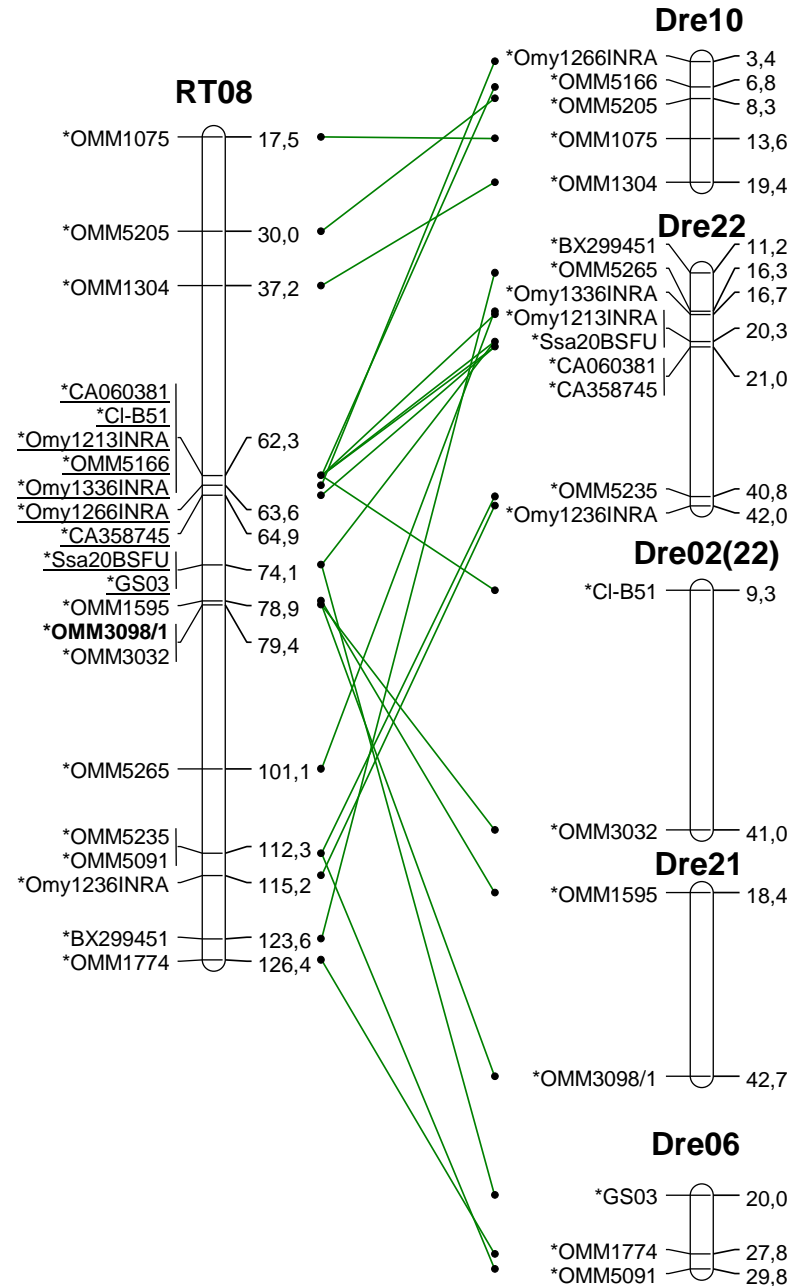

## RT09

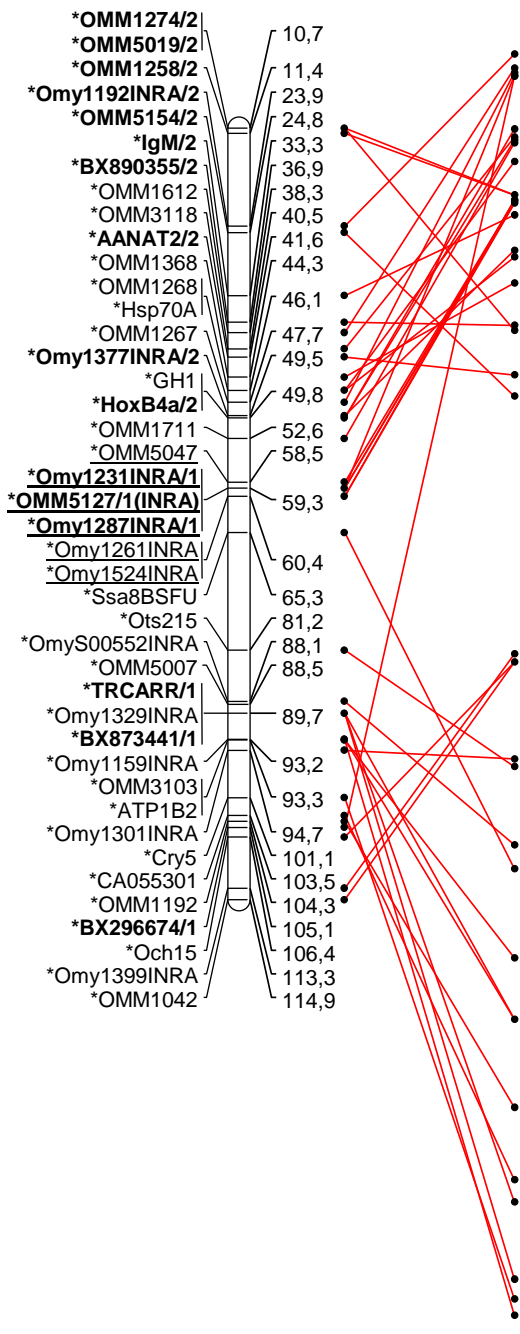

## Dre03

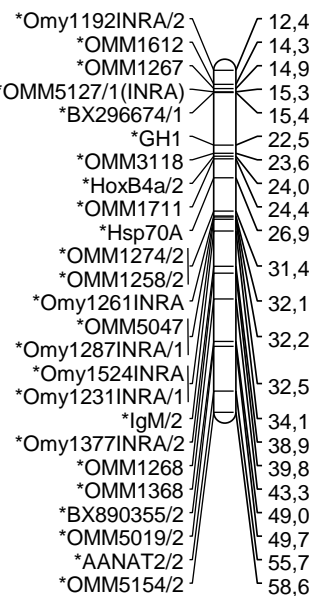

## Dre21(05)

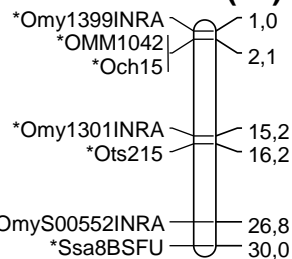

## Dre05(10/21)

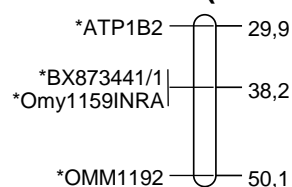

## Dre10(05)

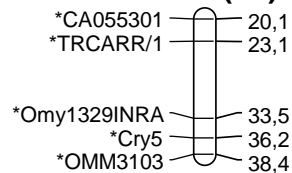

## RT10

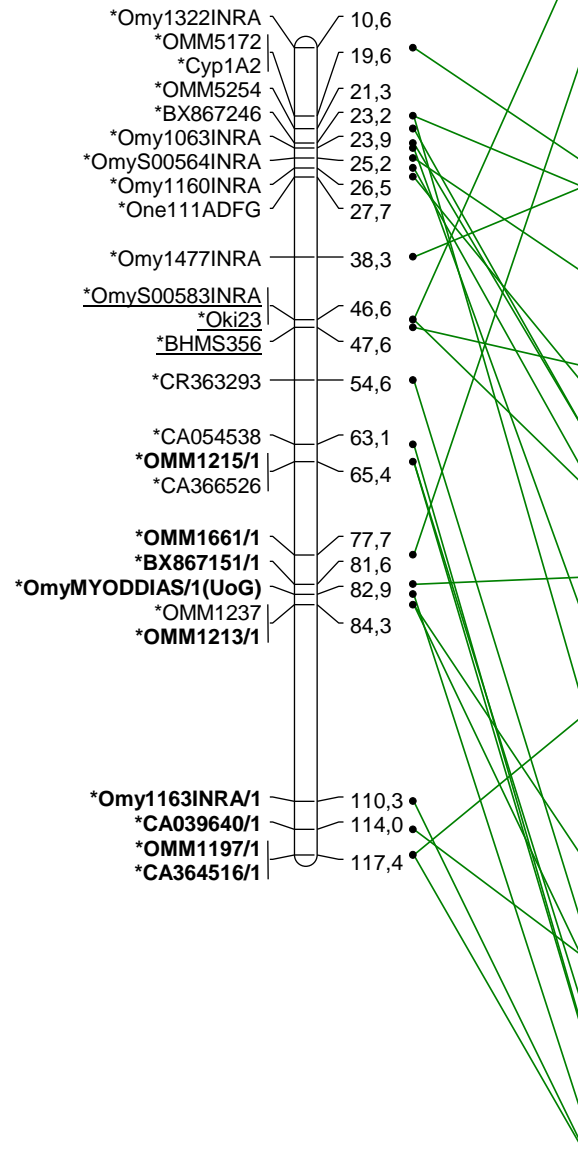

## Dr05(10)

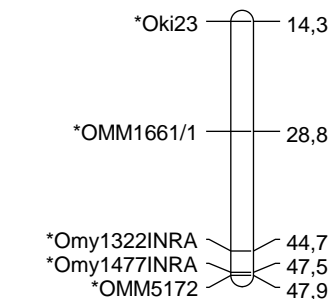

## Dre08(10)

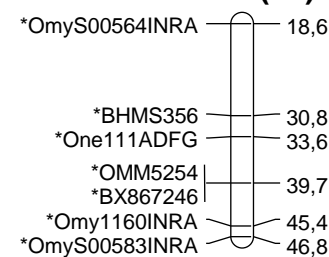

## Dre10(08)

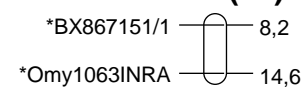

## Dre18(07)

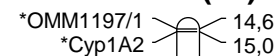

## Dre25(07)

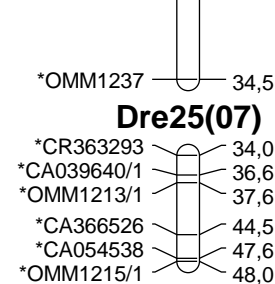

## Dre07

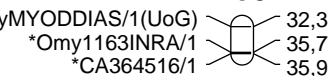

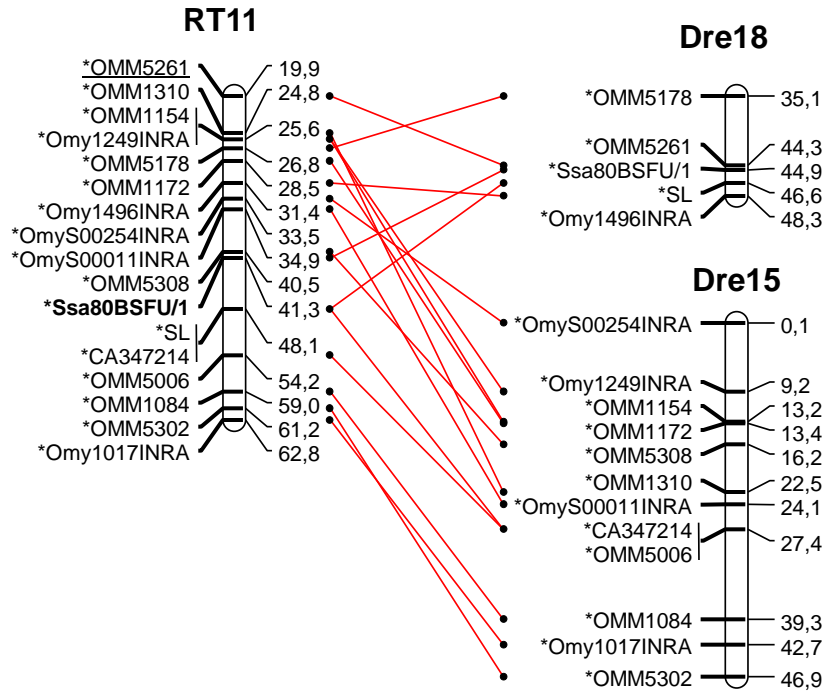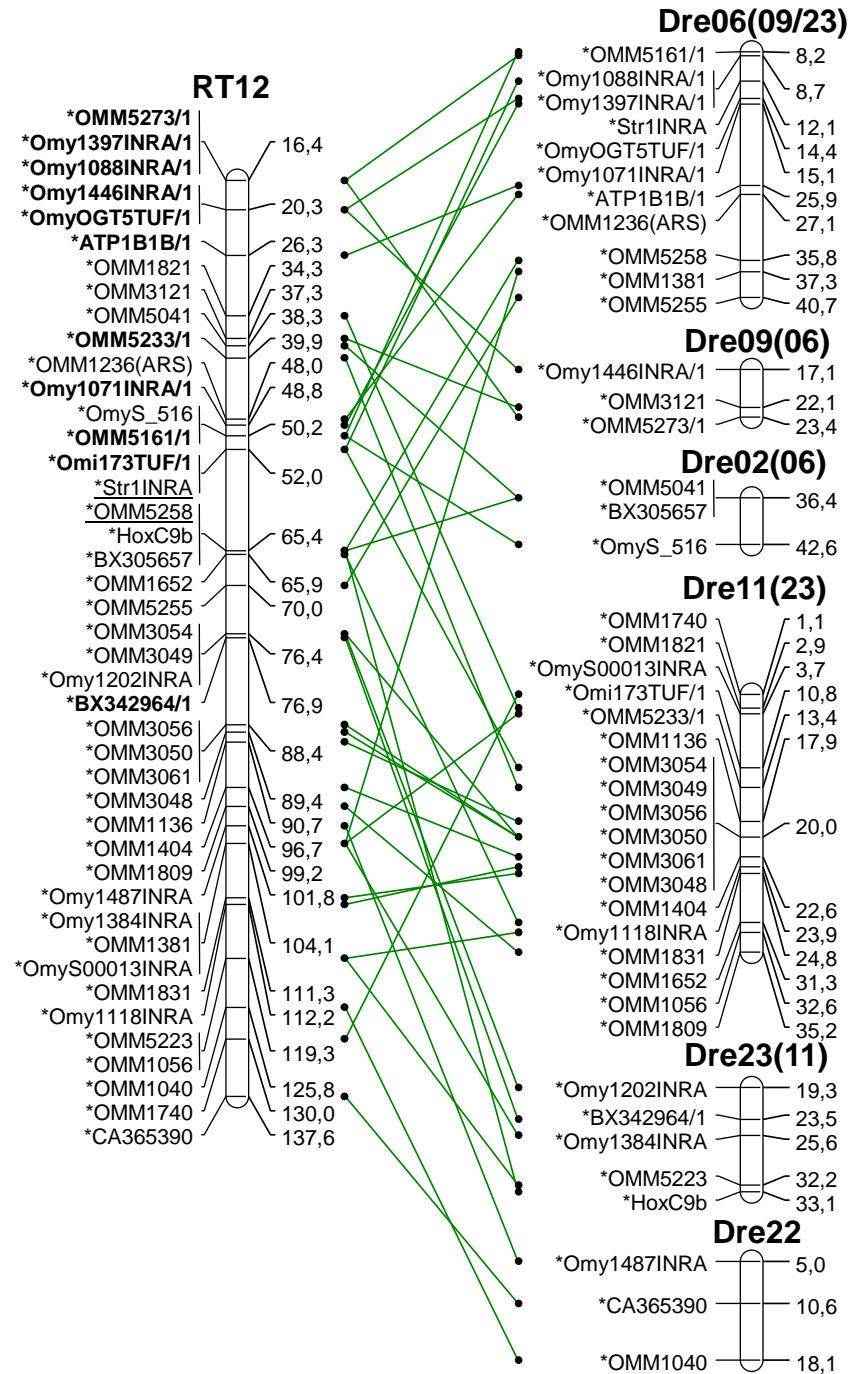

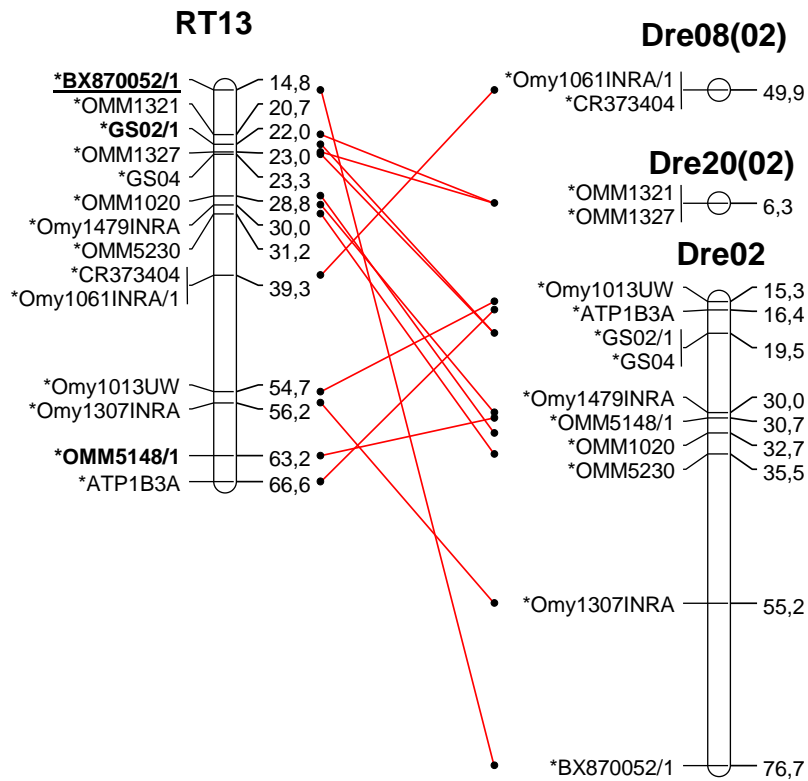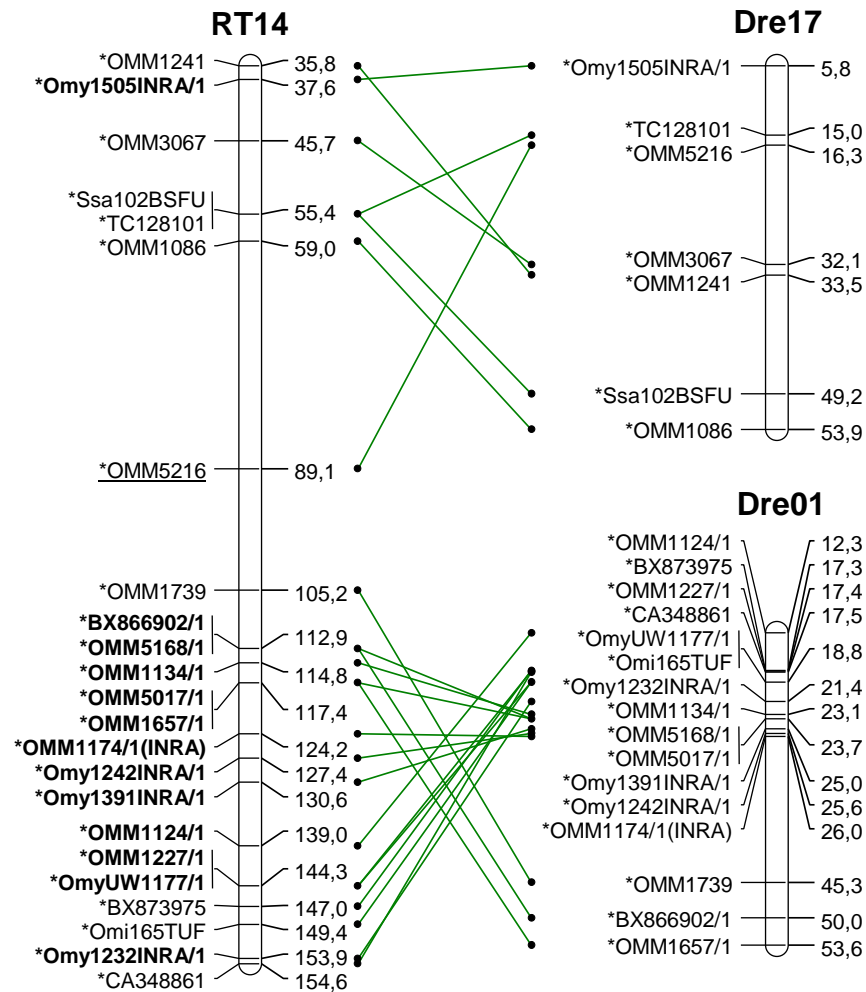

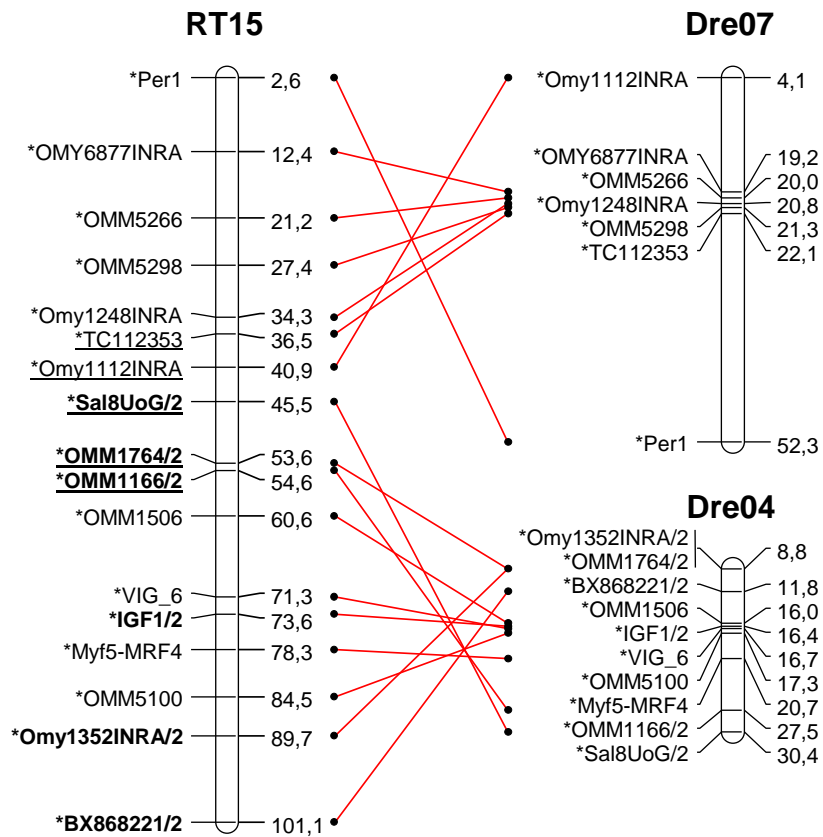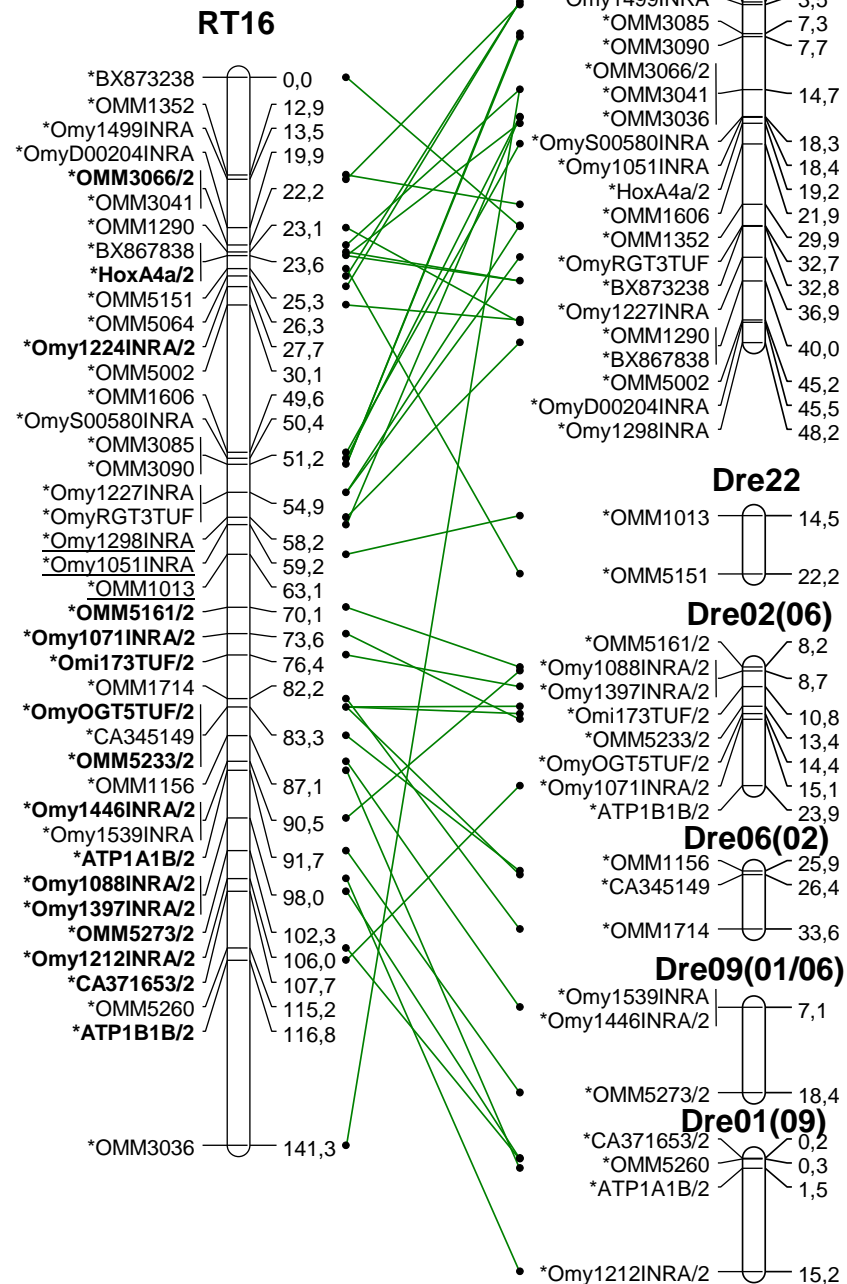

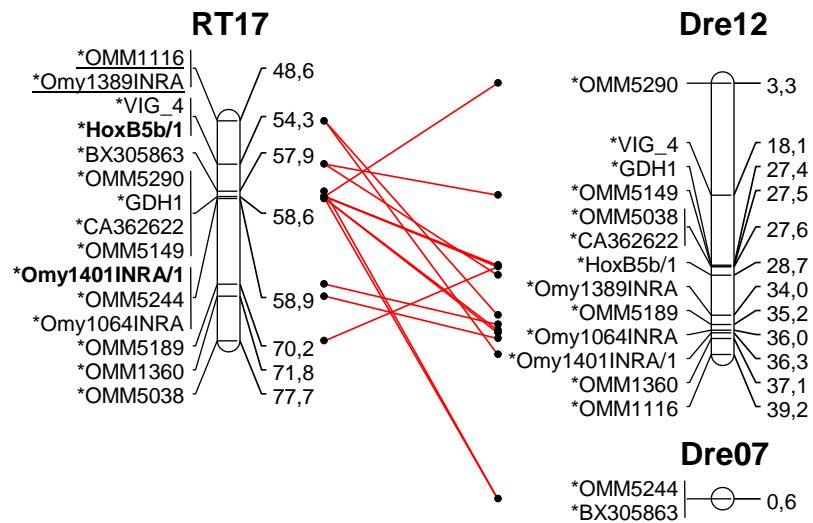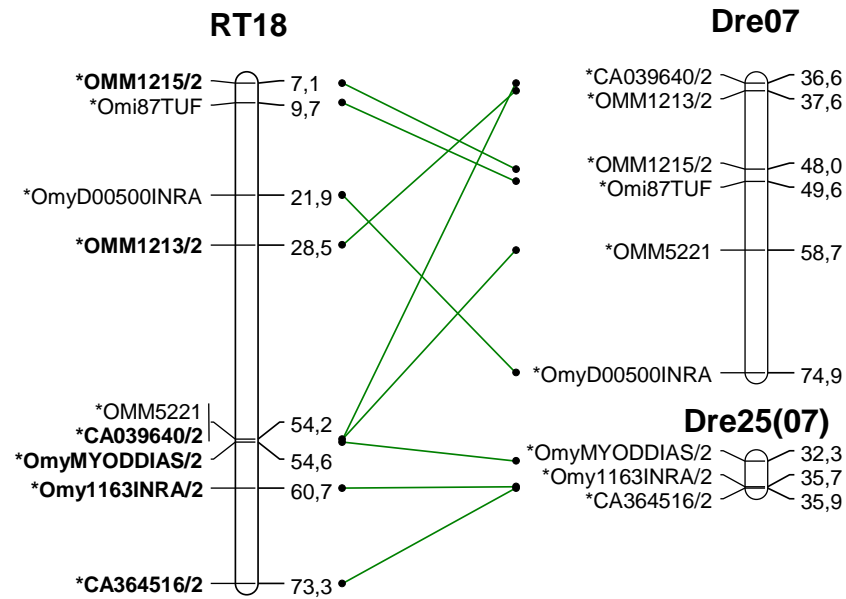

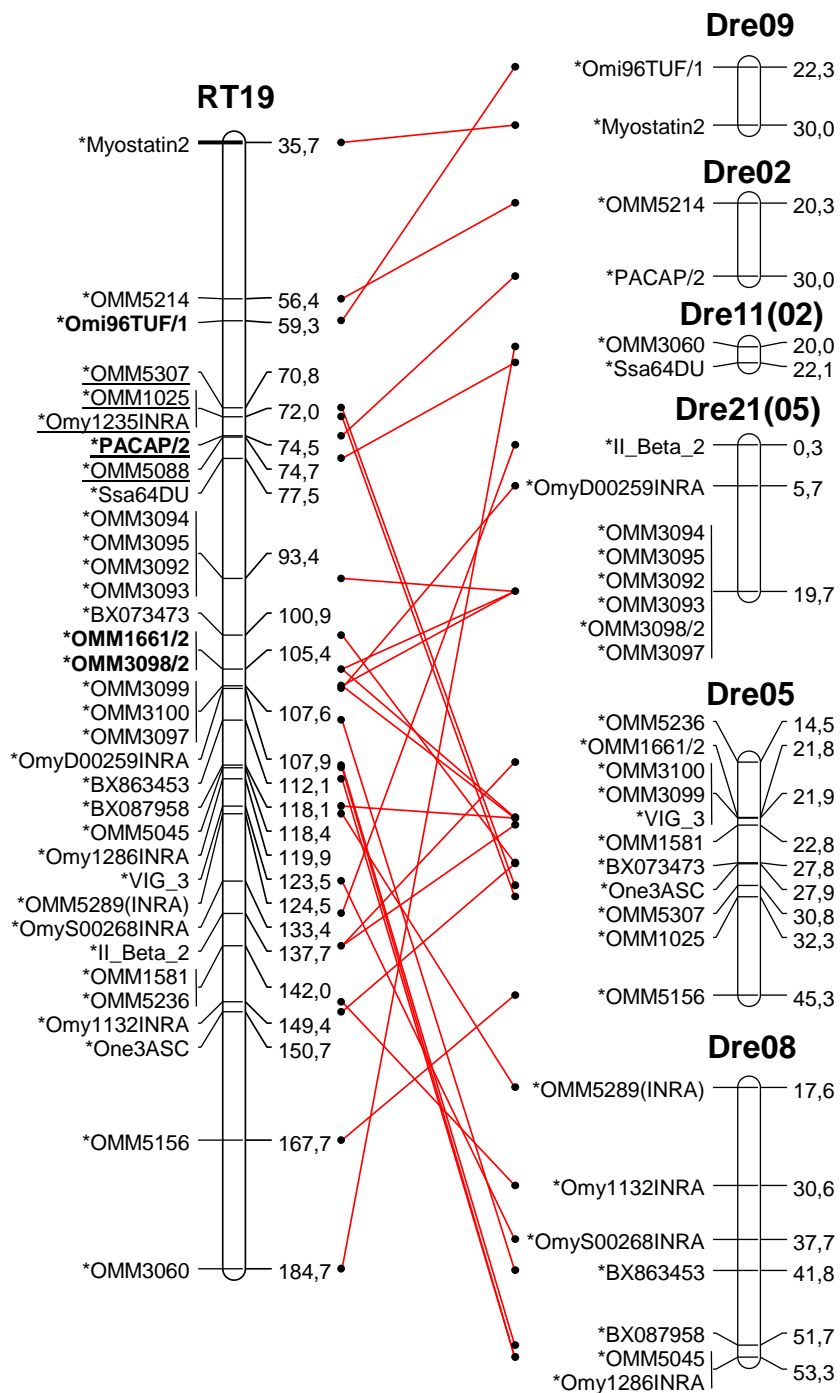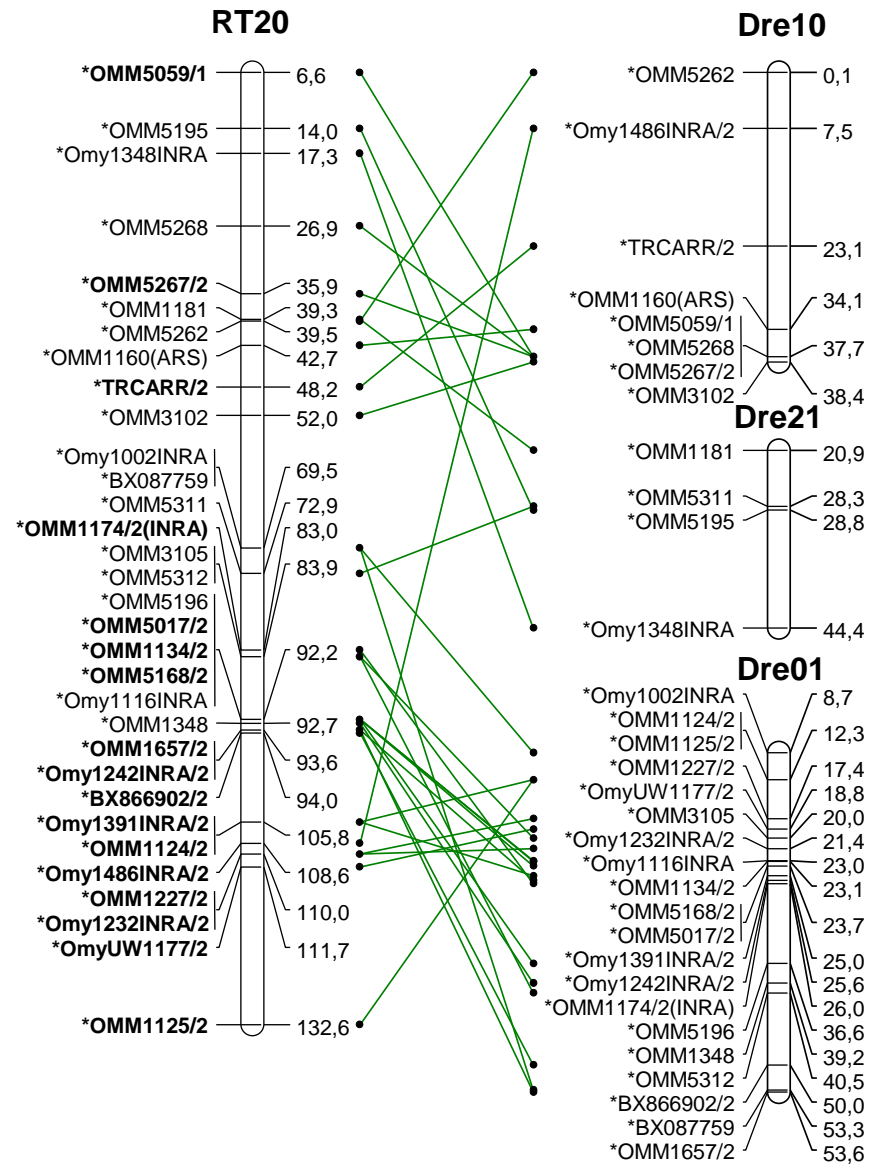

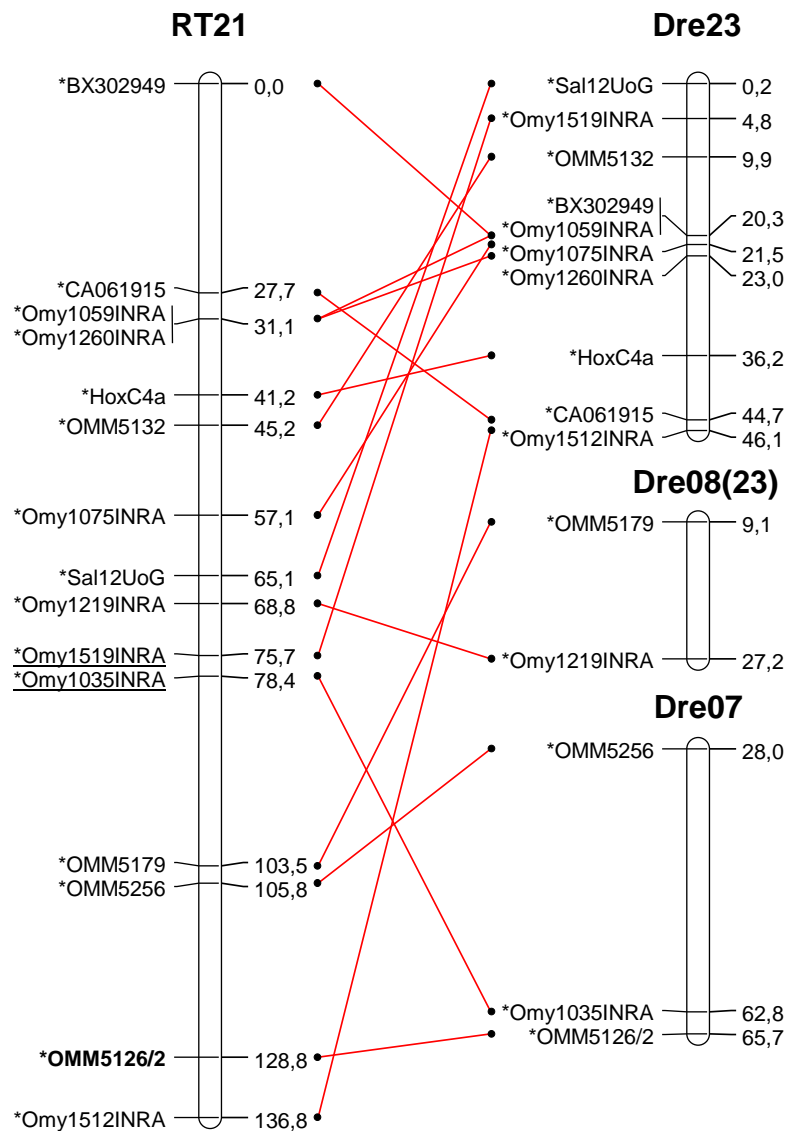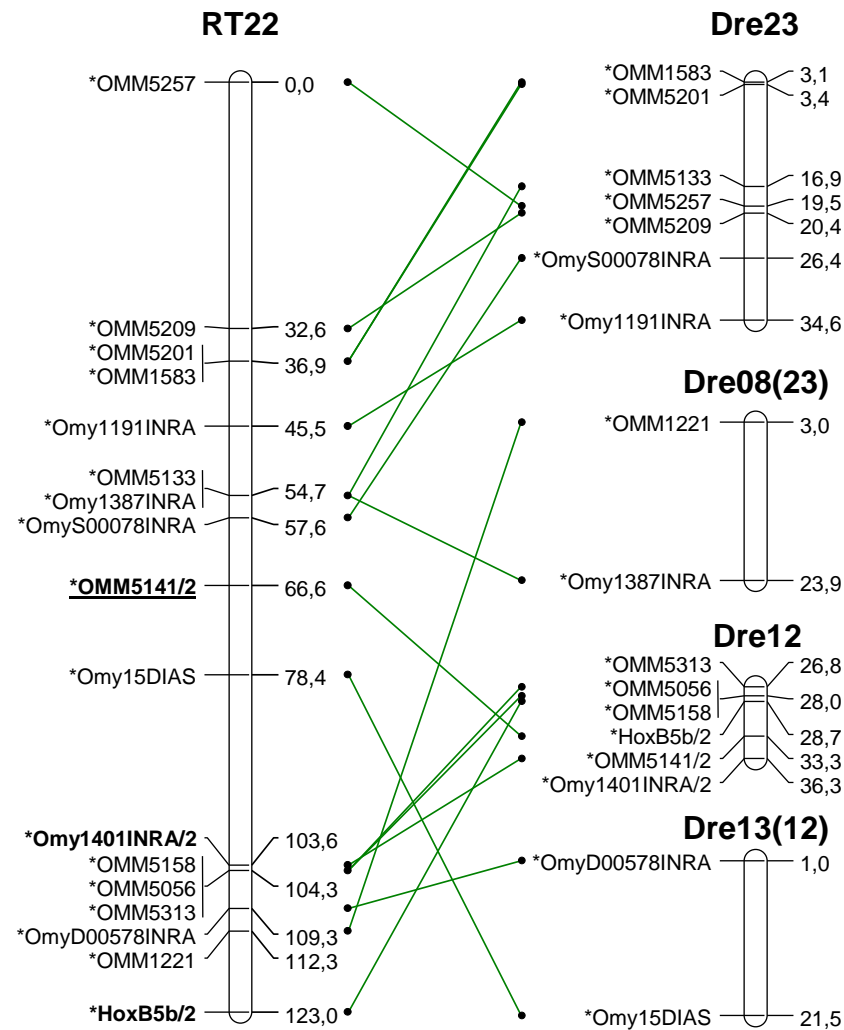

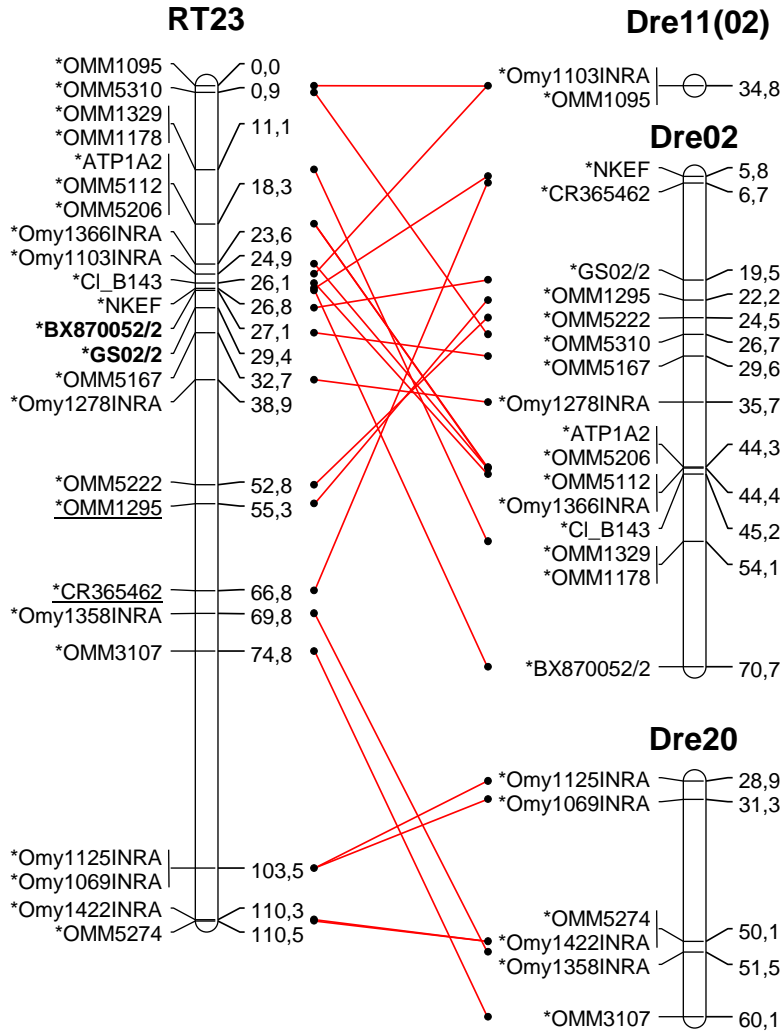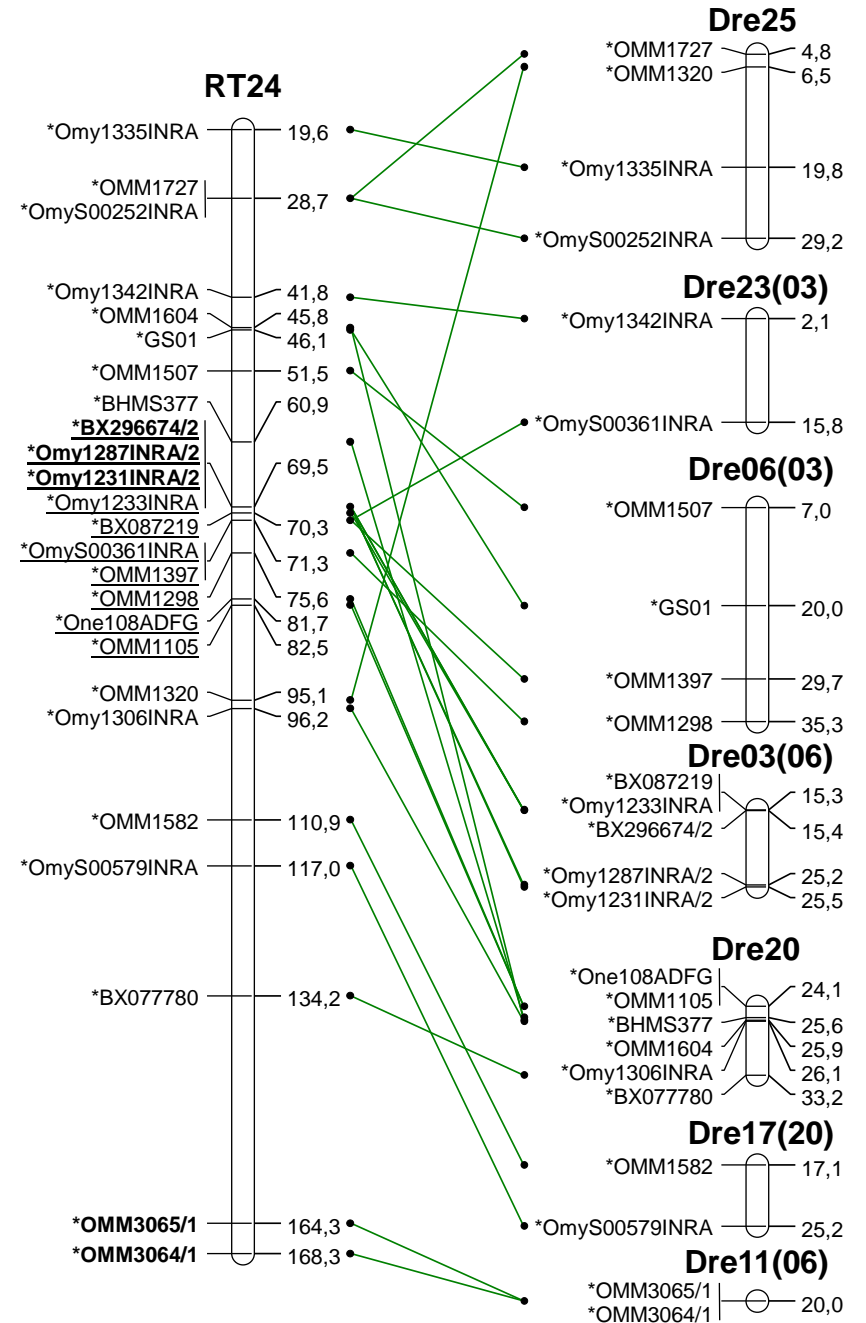

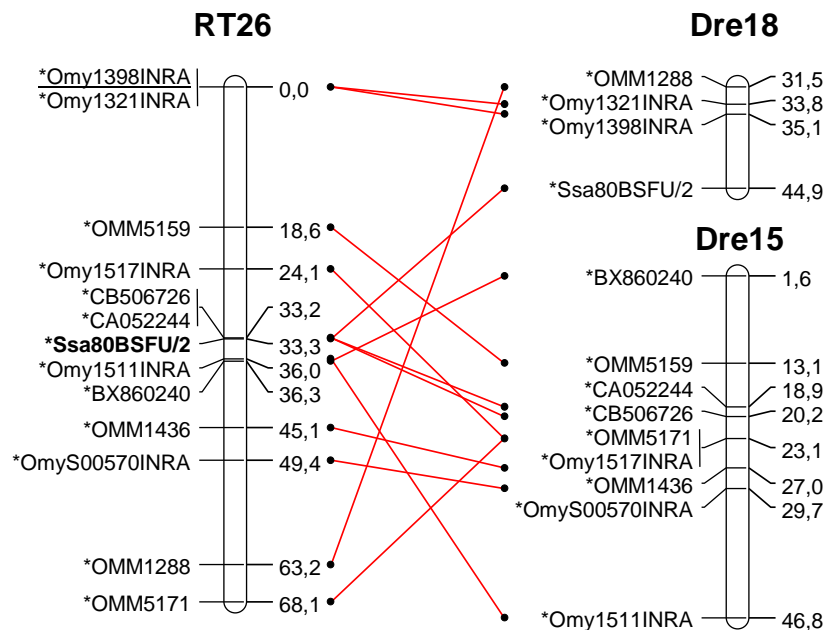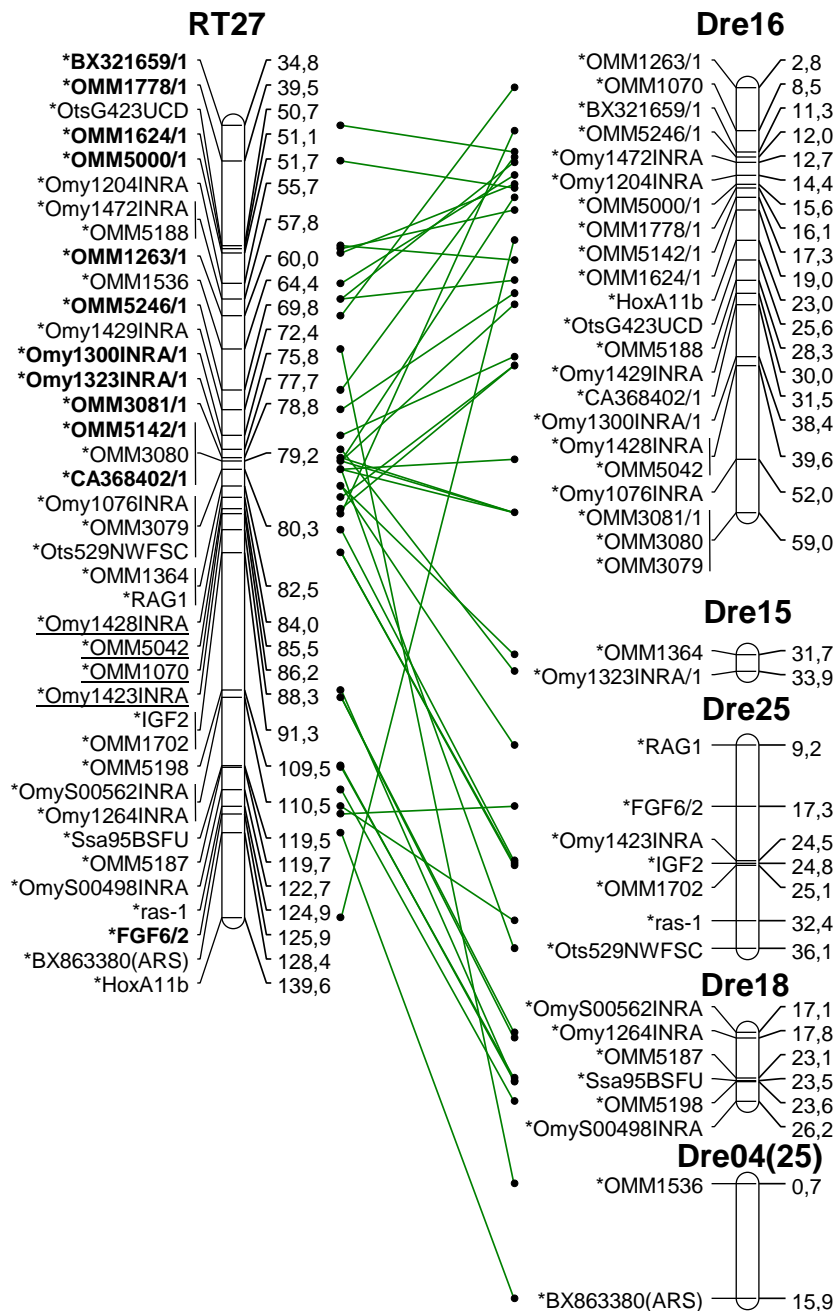

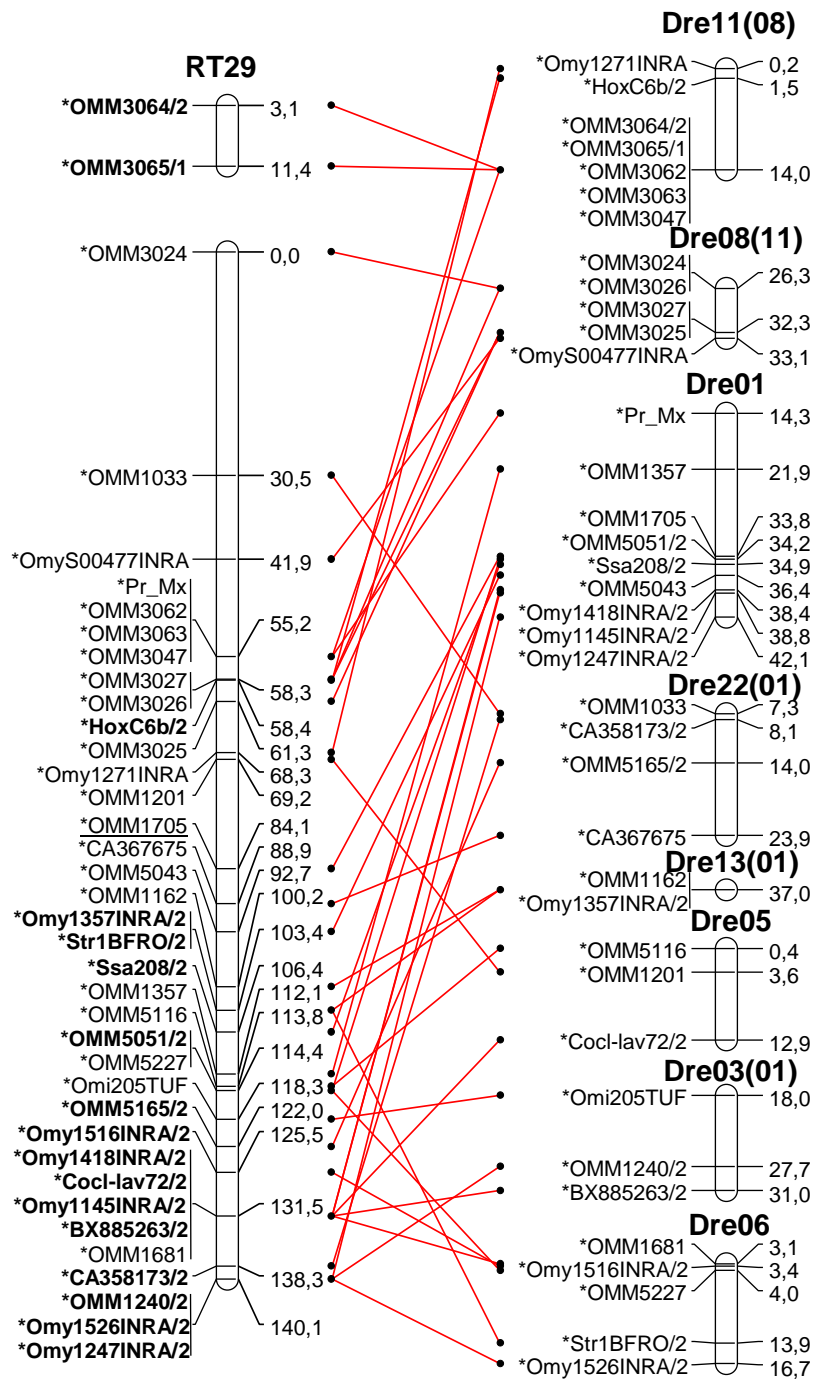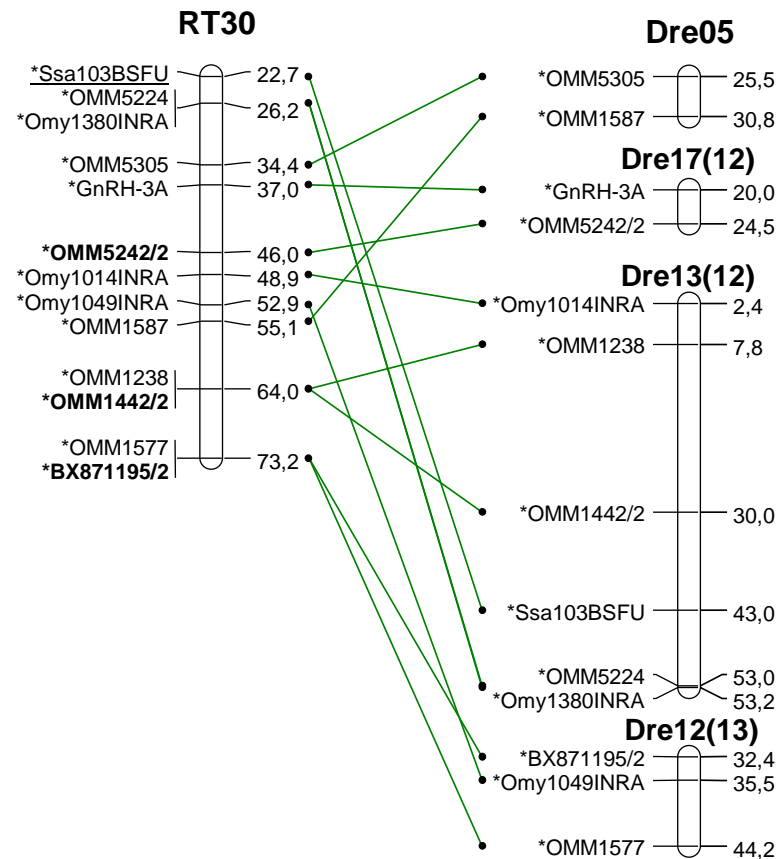

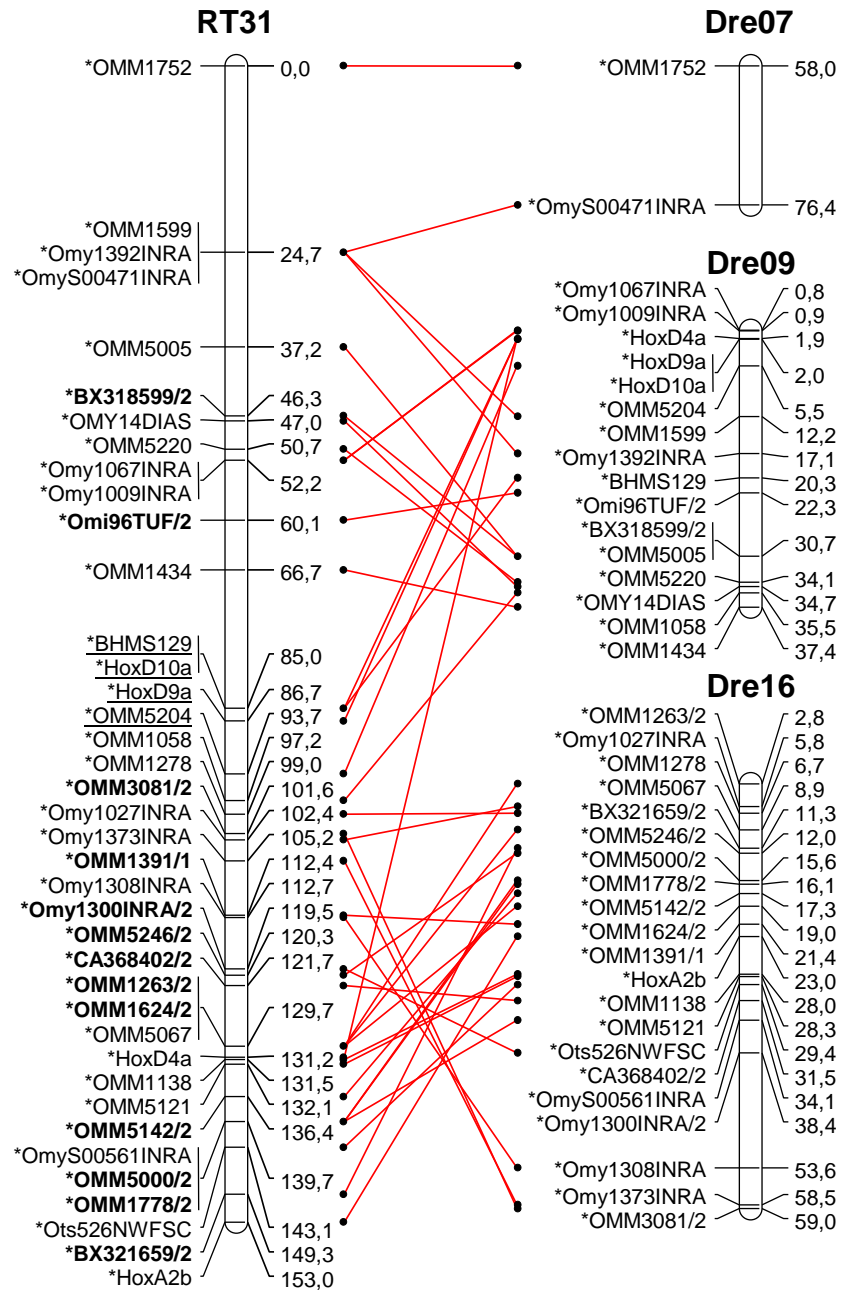

Supplement: Additional file 9 — Map alignments between chromosomes of rainbow trout and medaka, stickleback and zebrafish; underlined blue bold type marker names approximately localize centromeric regions; green and red lines distinguish between the two arms in acrocentric rainbow trout chromosomes; homologous marker positions in model species chromosomes are identified by Gene ID and sequence start position when blastx hits and blastn hits are used respectively. [file 1471-2156-13-15-S9.PDF]
