# Supplementary material for: A synthetic rainbow trout linkage map provides new insights into the salmonid whole genome duplication and the conservation of synteny among teleosts
Source: BMC Genet. 2012 Mar 16;13:15. doi: 10.1186/1471-2156-13-15 (PMC3368724; doi:10.1186/1471-2156-13-15)
Supplement: Additional file 8 — Map alignments between chromosomes of rainbow trout and medaka, stickleback and zebrafish; underlined blue bold type marker names approximately localize centromeric regions; green and red lines distinguish between the two arms in acrocentric rainbow trout chromosomes; homologous marker positions in model species chromosomes are identified by Gene ID and sequence start position when blastx hits and blastn hits are used respectively. [file 1471-2156-13-15-S8.PDF]

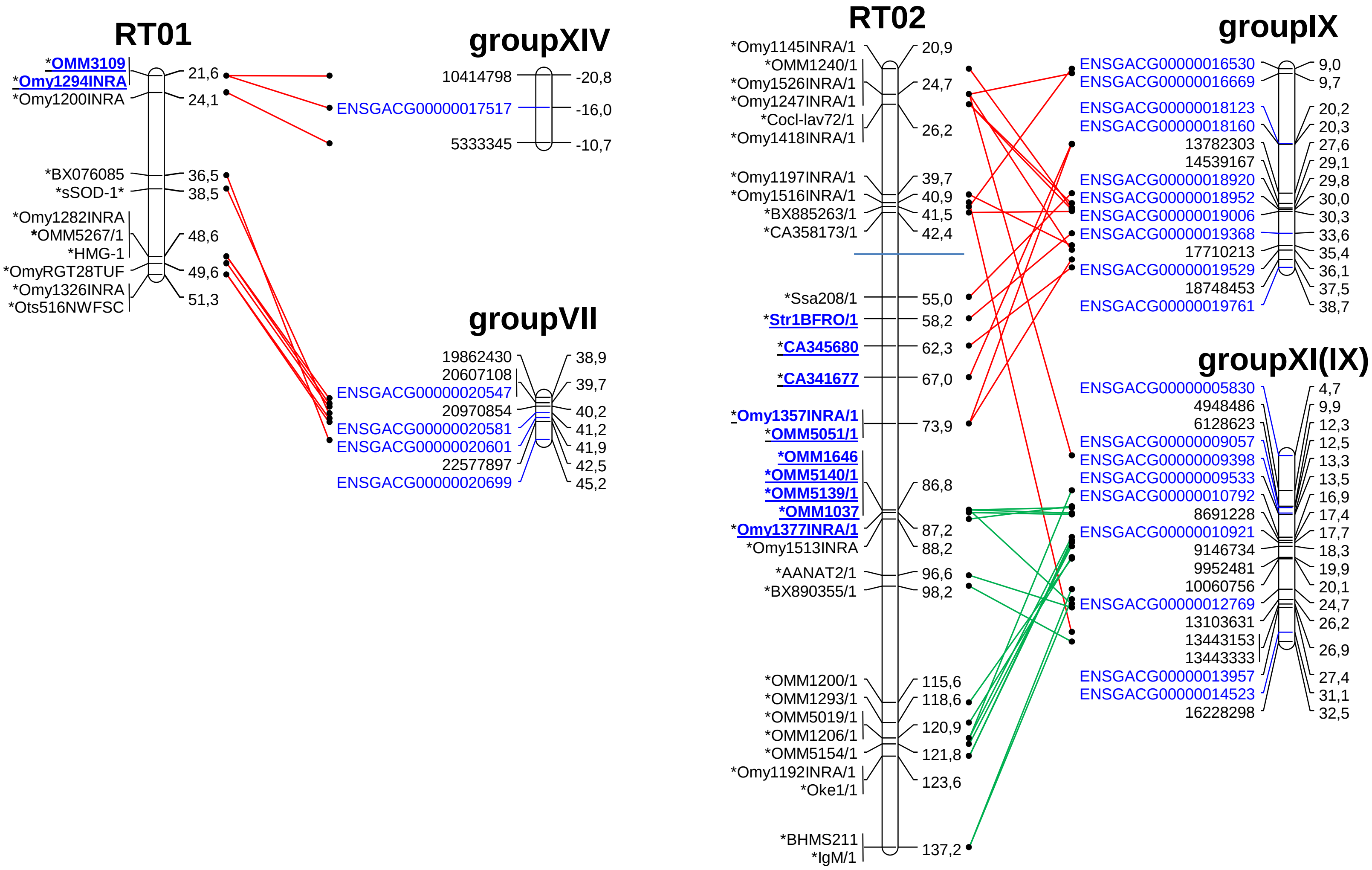
RT01
*OMM3109
*Omy1294INRA
*Omy1200INRA
*BX076085
sSOD-1
*Omy1282INRA
*OMM5267/1
*HMG-1
*OmyRGT28TUF
*Omy1326INRA
*Ots516NWFSC
21,6
24,1
36,5
38,5
48,6
49,6
51,3
groupXIV
10414798
ENSGACG00000017517
5333345
-20,8
-16,0
-10,7
groupVII
19862430
20607108
ENSGACG00000020547
20970854
ENSGACG00000020581
ENSGACG00000020601
22577897
ENSGACG00000020699
38,9
39,7
40,2
41,2
41,9
42,5
45,2
RT02
*Omy1145INRA/1
*OMM1240/1
*Omy1526INRA/1
*Omy1247INRA/1
*Cocl-lav72/1
*Omy1418INRA/1
*Omy1197INRA/1
*Omy1516INRA/1
*BX885263/1
*CA358173/1
*Ssa208/1
*Str1BFRO/1
*CA345680
*CA341677
*Omy1357INRA/1
*OMM5051/1
*OMM1646
*OMM5140/1
*OMM5139/1
*OMM1037
*Omy1377INRA/1
*Omy1513INRA
*AANAT2/1
*BX890355/1
*OMM1200/1
*OMM1293/1
*OMM5019/1
*OMM1206/1
*OMM5154/1
*Omy1192INRA/1
*Oke1/1
*BHMS211
*IgM/1
20,9
24,7
26,2
39,7
40,9
41,5
42,4
55,0
58,2
62,3
67,0
73,9
86,8
87,2
88,2
96,6
98,2
115,6
118,6
120,9
121,8
123,6
137,2
groupIX
ENSGACG00000016530
ENSGACG00000016669
ENSGACG00000018123
ENSGACG00000018160
13782303
14539167
ENSGACG00000018920
ENSGACG00000018952
ENSGACG00000019006
ENSGACG00000019368
17710213
ENSGACG00000019529
18748453
ENSGACG00000019761
9,0
9,7
20,2
20,3
27,6
29,1
29,8
30,0
30,3
33,6
35,4
36,1
37,5
38,7
groupXI(IX)
ENSGACG00000005830
4948486
6128623
ENSGACG00000009057
ENSGACG00000009398
ENSGACG00000009533
ENSGACG00000010792
8691228
ENSGACG00000010921
9146734
9952481
10060756
ENSGACG00000012769
13103631
13443153
13443333
ENSGACG00000013957
ENSGACG00000014523
16228298
4,7
9,9
12,3
12,5
13,3
13,5
16,9
17,4
17,7
18,3
19,9
20,1
24,7
26,2
26,9
27,4
31,1
32,5

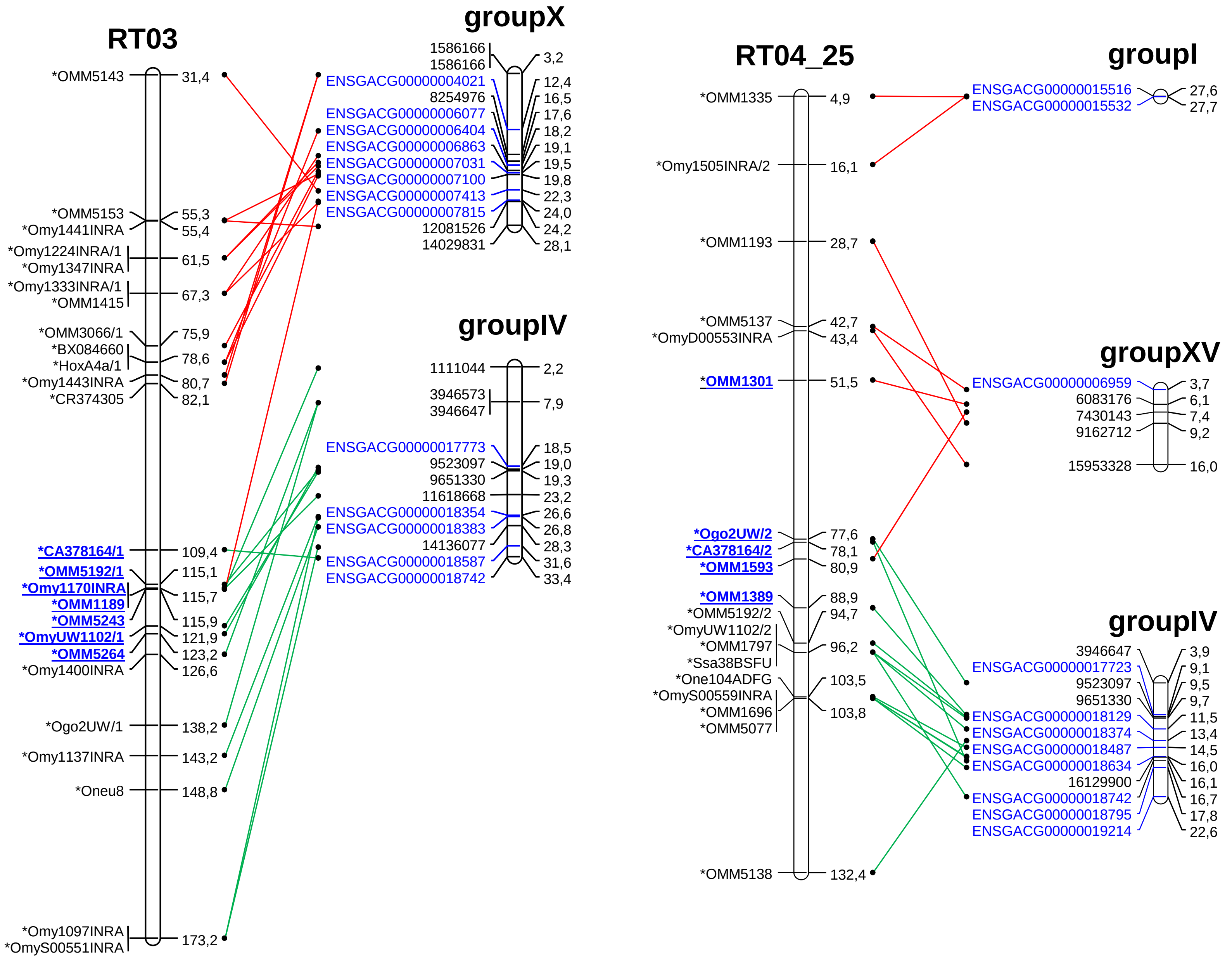
RT03
*OMM5143
31,4
*OMM5153
*Omy1441INRA
55,3
55,4
*Omy1224INRA/1
*Omy1347INRA
61,5
*Omy1333INRA/1
*OMM1415
67,3
*OMM3066/1
*BX084660
*HoxA4a/1
*Omy1443INRA
*CR374305
75,9
78,6
80,7
82,1
*CA378164/1
109,4
*OMM5192/1
*Omy1170INRA
*OMM1189
*OMM5243
*OmyUW1102/1
*OMM5264
*Omy1400INRA
115,1
115,7
115,9
121,9
123,2
126,6
*Ogo2UW/1
138,2
*Omy1137INRA
143,2
*Oneu8
148,8
*Omy1097INRA
*OmyS00551INRA
173,2
groupX
1586166
1586166
ENSGACG00000004021
8254976
ENSGACG00000006077
ENSGACG00000006404
ENSGACG00000006863
ENSGACG00000007031
ENSGACG00000007100
ENSGACG00000007413
ENSGACG00000007815
12081526
14029831
3,2
12,4
16,5
17,6
18,2
19,1
19,5
19,8
22,3
24,0
24,2
28,1
groupIV
1111044
3946573
3946647
ENSGACG00000017773
9523097
9651330
11618668
ENSGACG00000018354
ENSGACG00000018383
14136077
ENSGACG00000018587
ENSGACG00000018742
2,2
7,9
18,5
19,0
19,3
23,2
26,6
26,8
28,3
31,6
33,4
RT04_25
*OMM1335
4,9
*Omy1505INRA/2
16,1
*OMM1193
28,7
*OMM5137
*OmyD00553INRA
42,7
43,4
*OMM1301
51,5
*Ogo2UW/2
*CA378164/2
*OMM1593
77,6
78,1
80,9
*OMM1389
*OMM5192/2
*OmyUW1102/2
*OMM1797
*Ssa38BSFU
88,9
94,7
96,2
*One104ADFG
*OmyS00559INRA
*OMM1696
*OMM5077
103,5
103,8
*OMM5138
132,4
groupI
ENSGACG00000015516
ENSGACG00000015532
27,6
27,7
groupXV
ENSGACG00000006959
6083176
7430143
9162712
15953328
3,7
6,1
7,4
9,2
16,0
groupIV
3946647
ENSGACG00000017723
9523097
9651330
ENSGACG00000018129
ENSGACG00000018374
ENSGACG00000018487
ENSGACG00000018634
16129900
ENSGACG00000018742
ENSGACG00000018795
ENSGACG00000019214
3,9
9,1
9,5
9,7
11,5
13,4
14,5
16,0
16,1
16,7
17,8
22,6

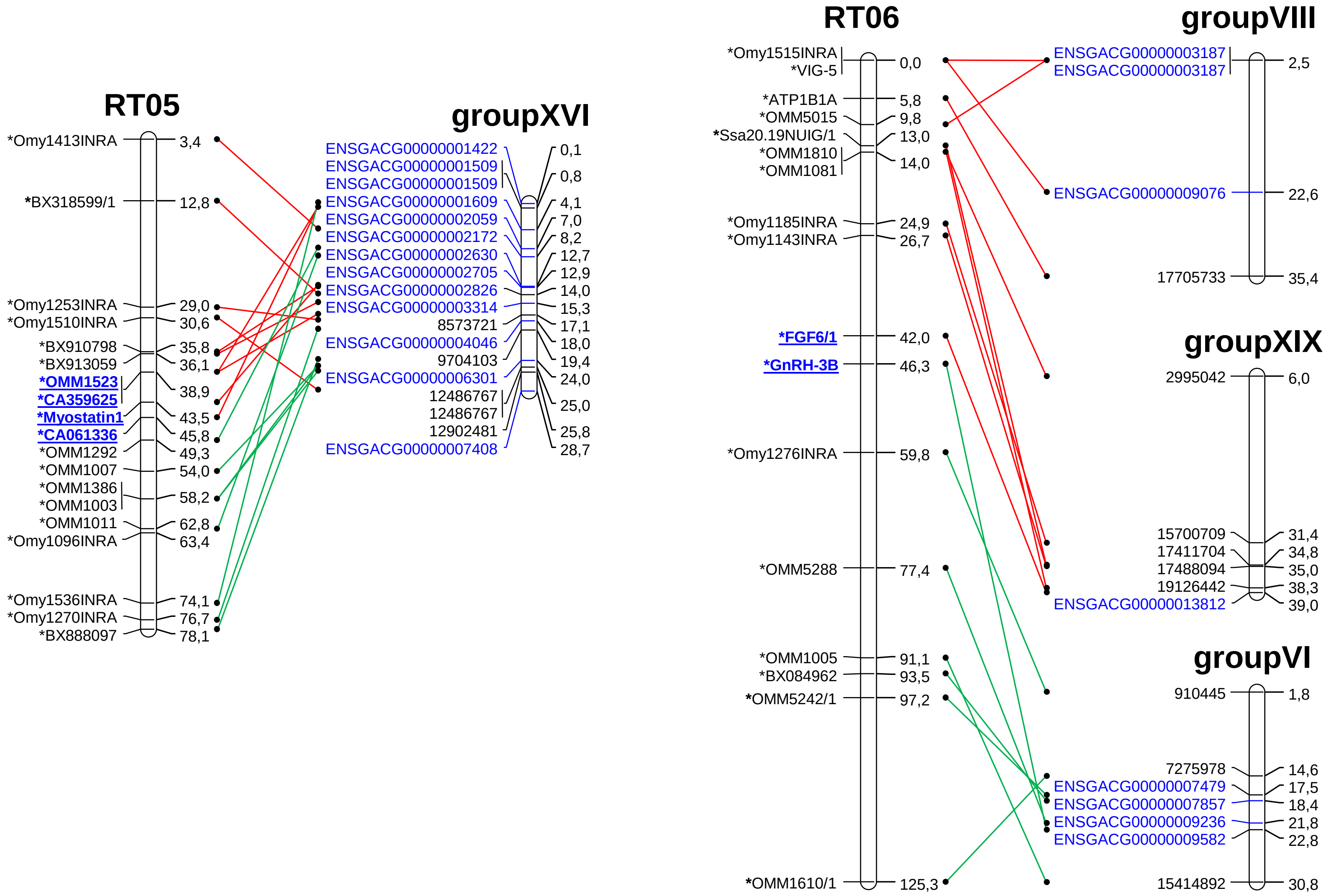

RT05
groupXVI
*Omy1413INRA
3,4
*BX318599/1
12,8
*Omy1253INRA
29,0
*Omy1510INRA
30,6
*BX910798
35,8
*BX913059
36,1
*OMM1523
38,9
*CA359625
43,5
*Myostatin1
45,8
*CA061336
49,3
*OMM1292
54,0
*OMM1007
58,2
*OMM1386
*OMM1003
62,8
*OMM1011
63,4
*Omy1096INRA
*Omy1536INRA
74,1
*Omy1270INRA
76,7
*BX888097
78,1
ENSGACG00000001422
0,1
ENSGACG00000001509
0,8
ENSGACG00000001509
4,1
ENSGACG00000001609
7,0
ENSGACG00000002059
8,2
ENSGACG00000002172
12,7
ENSGACG00000002630
12,9
ENSGACG00000002705
14,0
ENSGACG00000002826
15,3
ENSGACG00000003314
17,1
8573721
18,0
ENSGACG00000004046
19,4
9704103
24,0
ENSGACG00000006301
25,0
12486767
25,8
12486767
28,7
12902481
ENSGACG00000007408
RT06
groupVIII
*Omy1515INRA
*VIG-5
0,0
*ATP1B1A
5,8
*OMM5015
9,8
*Ssa20.19NUIG/1
13,0
*OMM1810
14,0
*OMM1081
*Omy1185INRA
24,9
*Omy1143INRA
26,7
*FGF6/1
42,0
*GnRH-3B
46,3
*Omy1276INRA
59,8
*OMM5288
77,4
*OMM1005
91,1
*BX084962
93,5
*OMM5242/1
97,2
*OMM1610/1
125,3
ENSGACG00000003187
2,5
ENSGACG00000003187
ENSGACG00000009076
22,6
17705733
35,4
groupXIX
2995042
6,0
15700709
31,4
17411704
34,8
17488094
35,0
19126442
38,3
ENSGACG00000013812
39,0
groupVI
910445
1,8
7275978
14,6
ENSGACG00000007479
17,5
ENSGACG00000007857
18,4
ENSGACG00000009236
21,8
ENSGACG00000009582
22,8
15414892
30,8

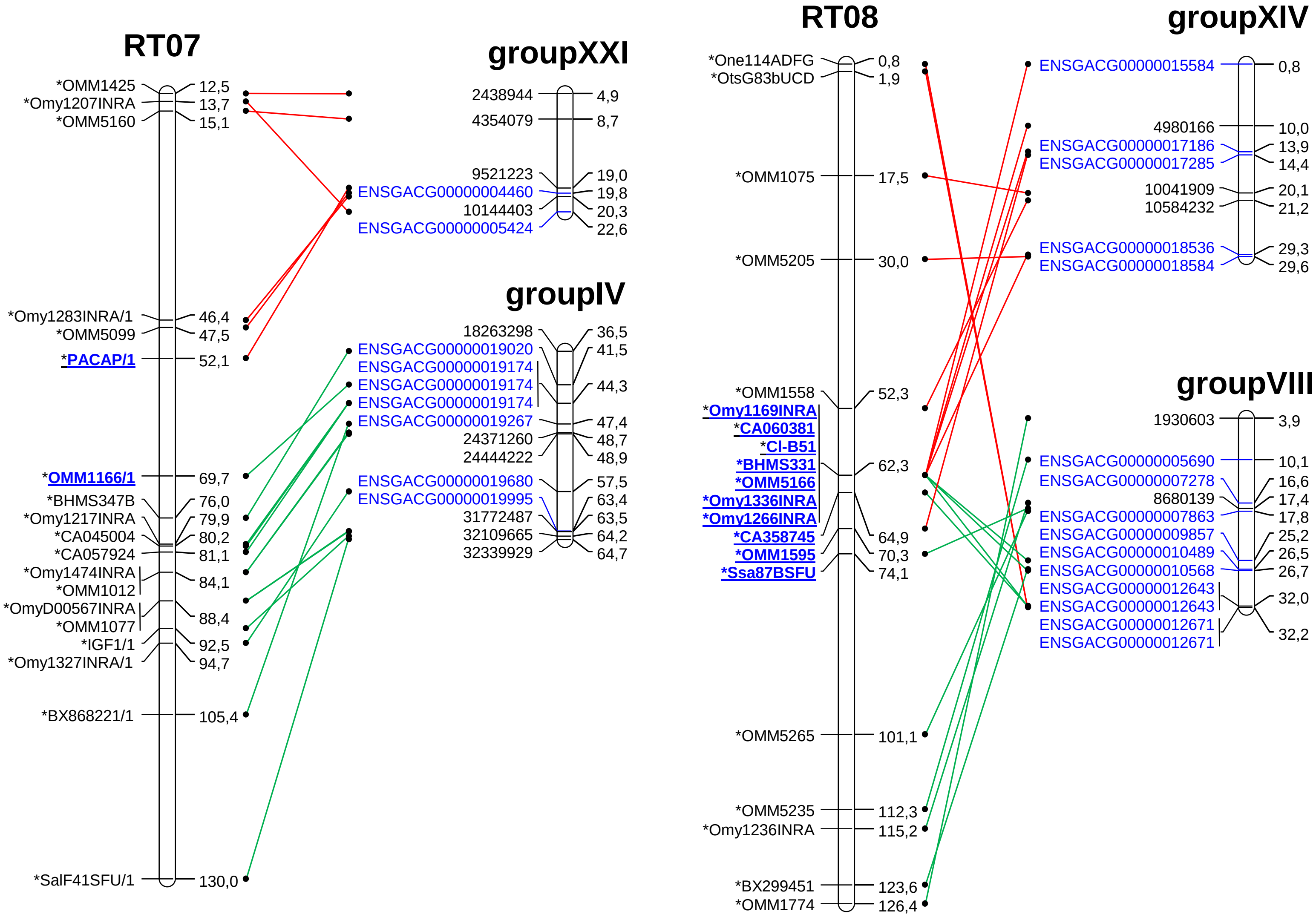

RT07
*OMM1425
*Omy1207INRA
*OMM5160
*Omy1283INRA/1
*OMM5099
*PACAP/1
*OMM1166/1
*BHMS347B
*Omy1217INRA
*CA045004
*CA057924
*Omy1474INRA
*OMM1012
*OmyD00567INRA
*OMM1077
*IGF1/1
*Omy1327INRA/1
*BX868221/1
*SalF41SFU/1
12,5
13,7
15,1
46,4
47,5
52,1
69,7
76,0
79,9
80,2
81,1
84,1
88,4
92,5
94,7
105,4
130,0
groupXXI
2438944
4354079
9521223
ENSGACG00000004460
10144403
ENSGACG00000005424
4,9
8,7
19,0
19,8
20,3
22,6
groupIV
18263298
ENSGACG00000019020
ENSGACG00000019174
ENSGACG00000019174
ENSGACG00000019174
ENSGACG00000019267
24371260
24444222
ENSGACG00000019680
ENSGACG00000019995
31772487
32109665
32339929
36,5
41,5
44,3
47,4
48,7
48,9
57,5
63,4
63,5
64,2
64,7
RT08
*One114ADFG
*OtsG83bUCD
*OMM1075
*OMM5205
*OMM1558
*Omy1169INRA
*CA060381
*Cl-B51
*BHMS331
*OMM5166
*Omy1336INRA
*Omy1266INRA
*CA358745
*OMM1595
*Ssa87BSFU
*OMM5265
*OMM5235
*Omy1236INRA
*BX299451
*OMM1774
0,8
1,9
17,5
30,0
52,3
62,3
64,9
70,3
74,1
101,1
112,3
115,2
123,6
126,4
groupXIV
ENSGACG00000015584
4980166
ENSGACG00000017186
ENSGACG00000017285
10041909
10584232
ENSGACG00000018536
ENSGACG00000018584
0,8
10,0
13,9
14,4
20,1
21,2
29,3
29,6
groupVIII
1930603
ENSGACG00000005690
ENSGACG00000007278
8680139
ENSGACG00000007863
ENSGACG00000009857
ENSGACG00000010489
ENSGACG00000010568
ENSGACG00000012643
ENSGACG00000012643
ENSGACG00000012671
ENSGACG00000012671
3,9
10,1
16,6
17,4
17,8
25,2
26,5
26,7
32,0
32,2

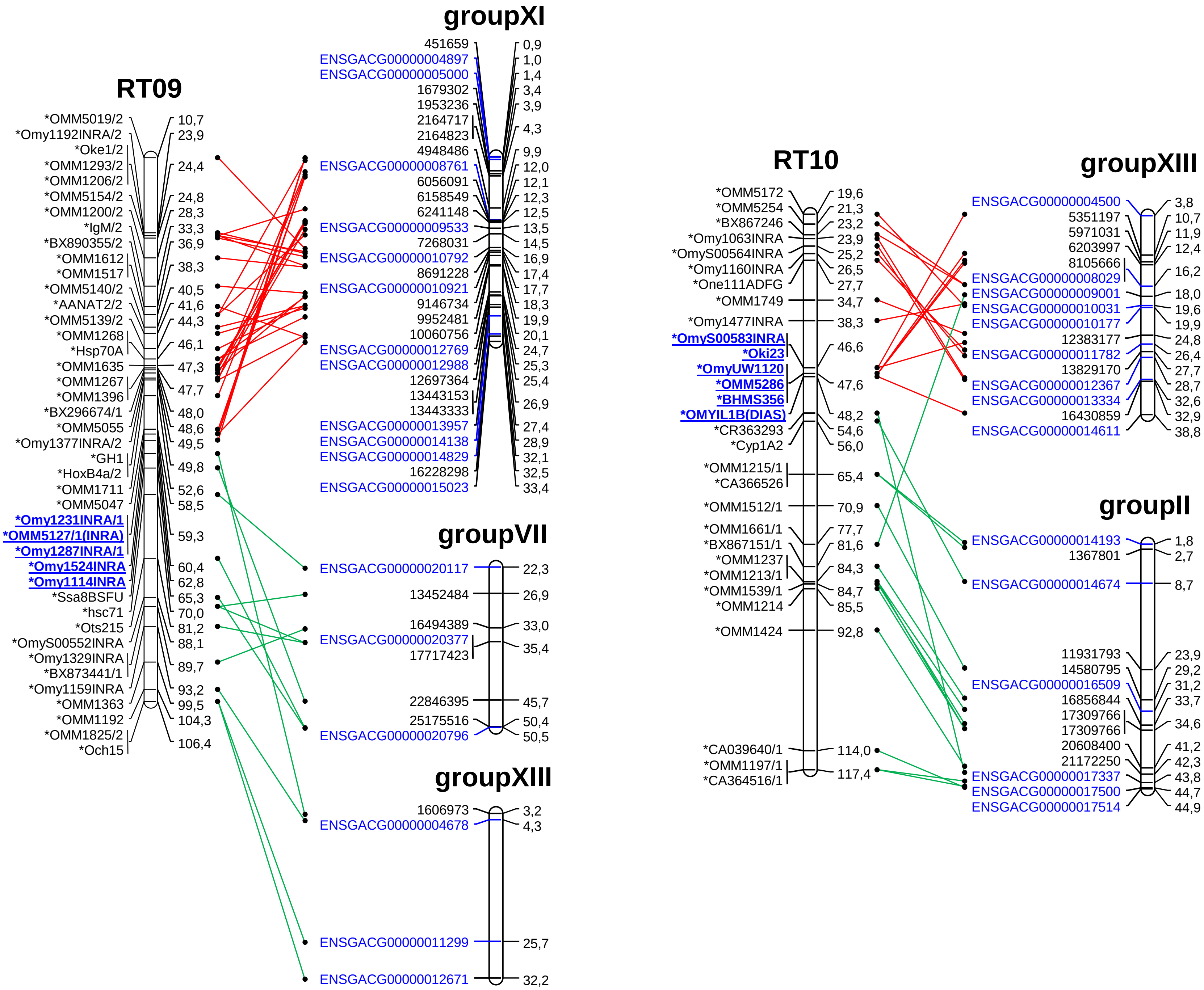

RT09
*OMM5019/2
*Omy1192INRA/2
*Oke1/2
*OMM1293/2
*OMM1206/2
*OMM5154/2
*OMM1200/2
*IgM/2
*BX890355/2
*OMM1612
*OMM1517
*OMM5140/2
*AANAT2/2
*OMM5139/2
*OMM1268
*Hsp70A
*OMM1635
*OMM1267
*OMM1396
*BX296674/1
*OMM5055
*Omy1377INRA/2
*GH1
*HoxB4a/2
*OMM1711
*OMM5047
*Omy1231INRA/1
*OMM5127/1(INRA)
*Omy1287INRA/1
*Omy1524INRA
*Omy1114INRA
*Ssa8BSFU
*hsc71
*Ots215
*OmyS00552INRA
*Omy1329INRA
*BX873441/1
*Omy1159INRA
*OMM1363
*OMM1192
*OMM1825/2
*Och15
10,7
23,9
24,4
24,8
28,3
33,3
36,9
38,3
40,5
41,6
44,3
46,1
47,3
47,7
48,0
48,6
49,5
49,8
52,6
58,5
59,3
60,4
62,8
65,3
70,0
81,2
88,1
89,7
93,2
99,5
104,3
106,4
groupXI
451659
ENSGACG00000004897
ENSGACG00000005000
1679302
1953236
2164717
2164823
4948486
ENSGACG00000008761
6056091
6158549
6241148
ENSGACG00000009533
7268031
ENSGACG00000010792
8691228
ENSGACG00000010921
9146734
9952481
10060756
ENSGACG00000012769
ENSGACG00000012988
12697364
13443153
13443333
ENSGACG00000013957
ENSGACG00000014138
ENSGACG00000014829
16228298
ENSGACG00000015023
0,9
1,0
1,4
3,4
3,9
4,3
9,9
12,0
12,1
12,3
12,5
13,5
14,5
16,9
17,4
17,7
18,3
19,9
20,1
24,7
25,3
25,4
26,9
27,4
28,9
32,1
32,5
33,4
groupVII
ENSGACG00000020117
13452484
16494389
ENSGACG00000020377
17717423
22846395
25175516
ENSGACG00000020796
22,3
26,9
33,0
35,4
45,7
50,4
50,5
groupXIII
1606973
ENSGACG00000004678
ENSGACG00000011299
ENSGACG00000012671
3,2
4,3
25,7
32,2
RT10
*OMM5172
*OMM5254
*BX867246
*Omy1063INRA
*OmyS00564INRA
*Omy1160INRA
*One111ADFG
*OMM1749
*Omy1477INRA
*OmyS00583INRA
*Oki23
*OmyUW1120
*OMM5286
*BHMS356
*OMYIL1B(DIAS)
*CR363293
*Cyp1A2
*OMM1215/1
*CA366526
*OMM1512/1
*OMM1661/1
*BX867151/1
*OMM1237
*OMM1213/1
*OMM1539/1
*OMM1214
*OMM1424
*CA039640/1
*OMM1197/1
*CA364516/1
19,6
21,3
23,2
23,9
25,2
26,5
27,7
34,7
38,3
46,6
47,6
48,2
54,6
56,0
65,4
70,9
77,7
81,6
84,3
84,7
85,5
92,8
114,0
117,4
groupXIII
ENSGACG00000004500
5351197
5971031
6203997
8105666
ENSGACG00000008029
ENSGACG00000009001
ENSGACG00000010031
ENSGACG00000010177
12383177
ENSGACG00000011782
13829170
ENSGACG00000012367
ENSGACG00000013334
16430859
ENSGACG00000014611
3,8
10,7
11,9
12,4
16,2
18,0
19,6
19,9
24,8
26,4
27,7
28,7
32,6
32,9
38,8
groupII
ENSGACG00000014193
1367801
ENSGACG00000014674
11931793
14580795
ENSGACG00000016509
16856844
17309766
17309766
20608400
21172250
ENSGACG00000017337
ENSGACG00000017500
ENSGACG00000017514
1,8
2,7
8,7
23,9
29,2
31,2
33,7
34,6
41,2
42,3
43,8
44,7
44,9

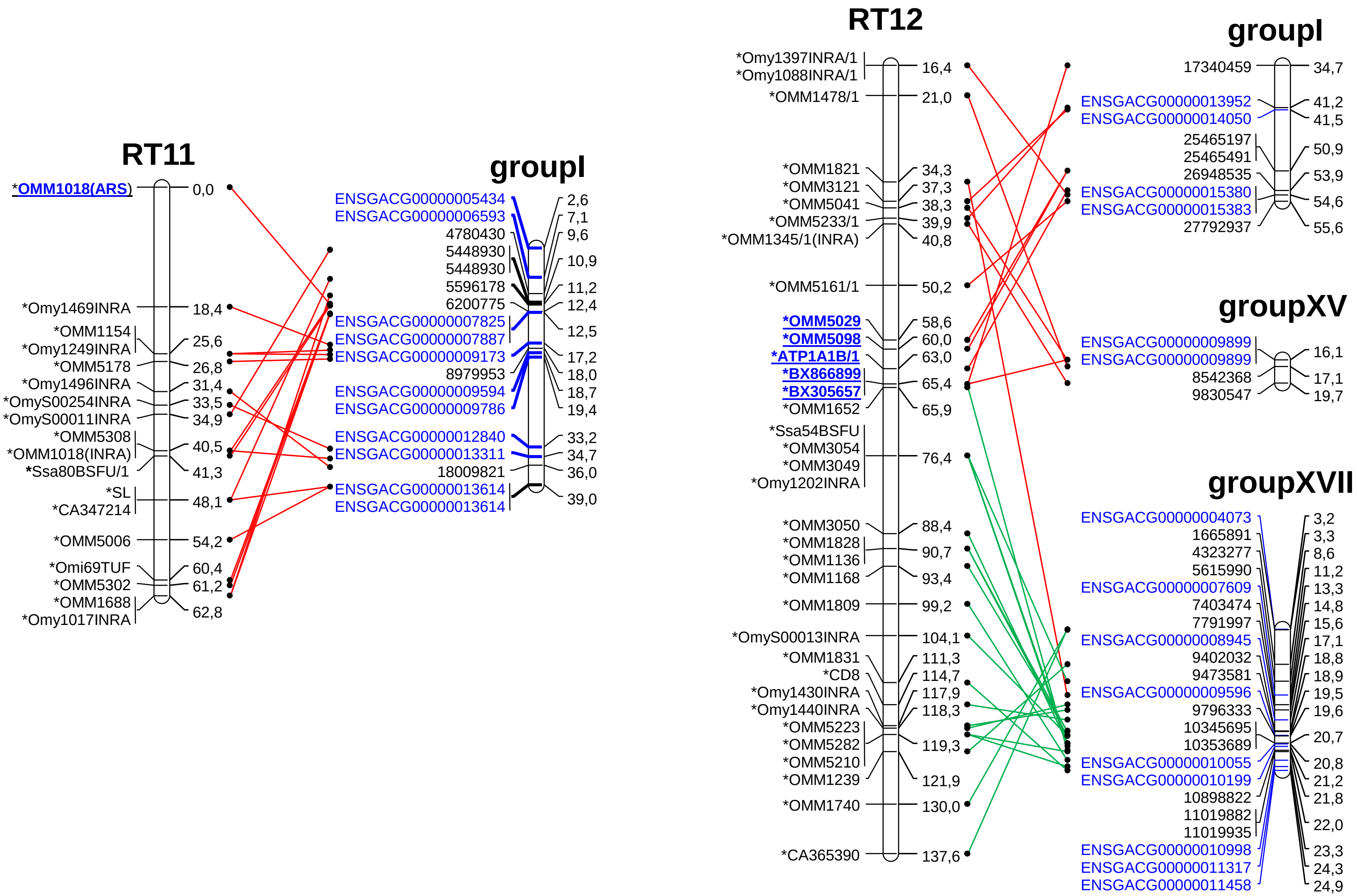
RT11
*OMM1018(ARS) 0,0
*Omy1469INRA 18,4
*OMM1154 25,6
*Omy1249INRA
*OMM5178 26,8
*Omy1496INRA 31,4
*OmyS00254INRA 33,5
*OmyS00011INRA 34,9
*OMM5308 40,5
*OMM1018(INRA)
*Ssa80BSFU/1 41,3
*SL 48,1
*CA347214
*OMM5006 54,2
*Omi69TUF 60,4
*OMM5302 61,2
*OMM1688 62,8
*Omy1017INRA
groupI
ENSGACG00000005434 2,6
ENSGACG00000006593 7,1
4780430 9,6
5448930 10,9
5448930 11,2
5596178 12,4
6200775 12,5
ENSGACG00000007825 17,2
ENSGACG00000007887 18,0
ENSGACG00000009173 18,7
8979953 19,4
ENSGACG00000009594
ENSGACG00000009786
ENSGACG00000012840 33,2
ENSGACG00000013311 34,7
18009821 36,0
ENSGACG00000013614 39,0
ENSGACG00000013614
RT12
*Omy1397INRA/1 16,4
*Omy1088INRA/1
*OMM1478/1 21,0
*OMM1821 34,3
*OMM3121 37,3
*OMM5041 38,3
*OMM5233/1 39,9
*OMM1345/1(INRA) 40,8
*OMM5161/1 50,2
*OMM5029 58,6
*OMM5098 60,0
*ATP1A1B/1 63,0
*BX866899 65,4
*BX305657 65,9
*OMM1652
*Ssa54BSFU
*OMM3054 76,4
*OMM3049
*Omy1202INRA
*OMM3050 88,4
*OMM1828 90,7
*OMM1136
*OMM1168 93,4
*OMM1809 99,2
*OmyS00013INRA 104,1
*OMM1831 111,3
*CD8 114,7
*Omy1430INRA 117,9
*Omy1440INRA 118,3
*OMM5223
*OMM5282 119,3
*OMM5210
*OMM1239 121,9
*OMM1740 130,0
*CA365390 137,6
groupI
17340459 34,7
ENSGACG00000013952 41,2
ENSGACG00000014050 41,5
25465197 50,9
25465491
26948535 53,9
ENSGACG00000015380 54,6
ENSGACG00000015383
27792937 55,6
groupXV
ENSGACG00000009899 16,1
ENSGACG00000009899 17,1
8542368 19,7
9830547
groupXVII
ENSGACG00000004073 3,2
1665891 3,3
4323277 8,6
5615990 11,2
ENSGACG00000007609 13,3
7403474 14,8
7791997 15,6
ENSGACG00000008945 17,1
9402032 18,8
9473581 18,9
ENSGACG00000009596 19,5
9796333 19,6
10345695 20,7
10353689
ENSGACG00000010055 20,8
ENSGACG00000010199 21,2
10898822 21,8
11019882 22,0
11019935
ENSGACG00000010998 23,3
ENSGACG00000011317 24,3
ENSGACG00000011458 24,9

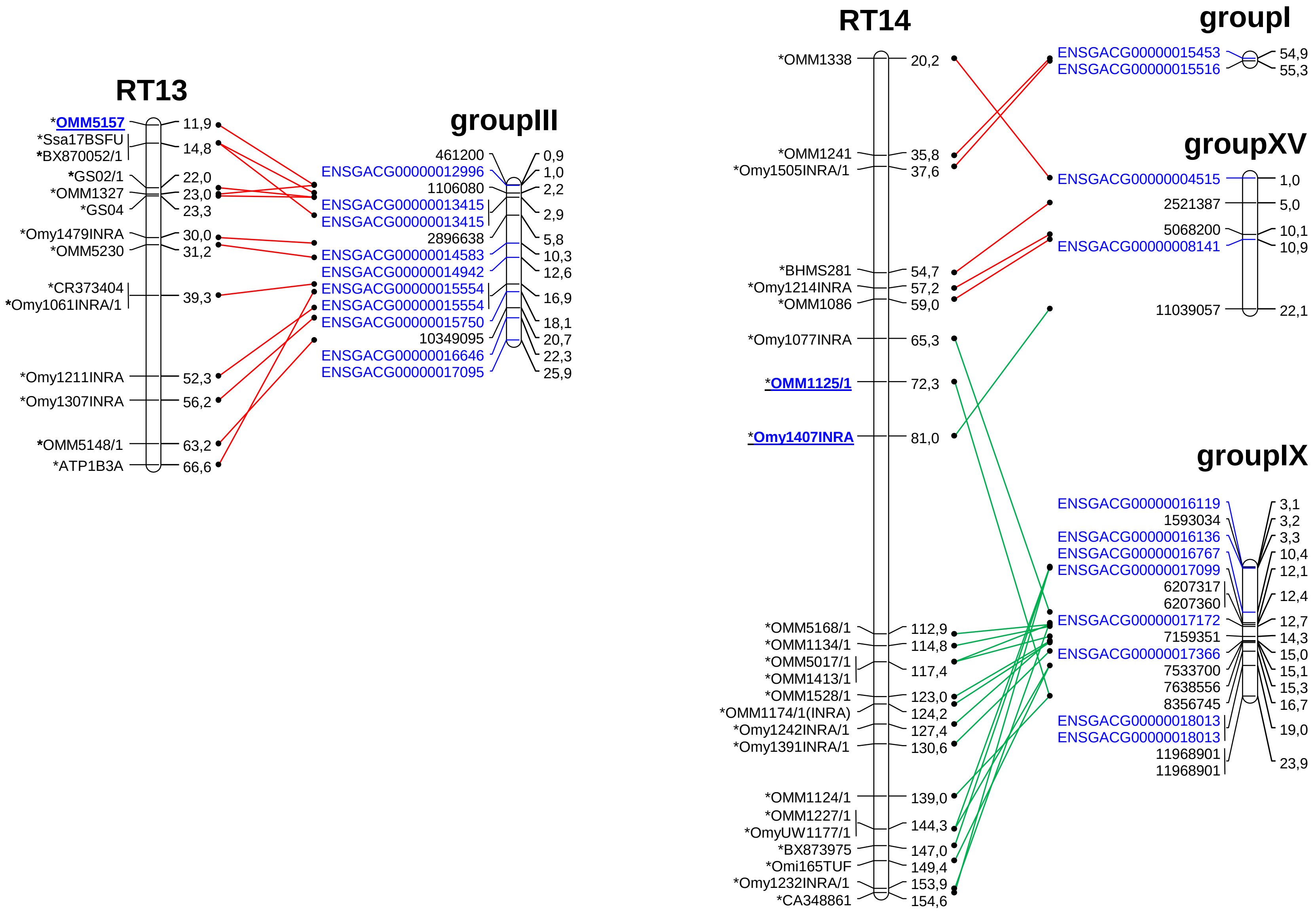
RT13
*OMM5157
*Ssa17BSFU
*BX870052/1
*GS02/1
*OMM1327
*GS04
*Omy1479INRA
*OMM5230
*CR373404
*Omy1061INRA/1
*Omy1211INRA
*Omy1307INRA
*OMM5148/1
*ATP1B3A
11,9
14,8
22,0
23,0
23,3
30,0
31,2
39,3
52,3
56,2
63,2
66,6
groupIII
461200
ENSGACG00000012996
1106080
ENSGACG00000013415
ENSGACG00000013415
2896638
ENSGACG00000014583
ENSGACG00000014942
ENSGACG00000015554
ENSGACG00000015554
ENSGACG00000015750
10349095
ENSGACG00000016646
ENSGACG00000017095
0,9
1,0
2,2
2,9
5,8
10,3
12,6
16,9
18,1
20,7
22,3
25,9
RT14
*OMM1338
*OMM1241
*Omy1505INRA/1
*BHMS281
*Omy1214INRA
*OMM1086
*Omy1077INRA
*OMM1125/1
*Omy1407INRA
*OMM5168/1
*OMM1134/1
*OMM5017/1
*OMM1413/1
*OMM1528/1
*OMM1174/1(INRA)
*Omy1242INRA/1
*Omy1391INRA/1
*OMM1124/1
*OMM1227/1
*OmyUW1177/1
*BX873975
*Omi165TUF
*Omy1232INRA/1
*CA348861
20,2
35,8
37,6
54,7
57,2
59,0
65,3
72,3
81,0
112,9
114,8
117,4
123,0
124,2
127,4
130,6
139,0
144,3
147,0
149,4
153,9
154,6
groupI
ENSGACG00000015453
ENSGACG00000015516
54,9
55,3
groupXV
ENSGACG00000004515
2521387
5068200
ENSGACG00000008141
11039057
1,0
5,0
10,1
10,9
22,1
groupIX
ENSGACG00000016119
1593034
ENSGACG00000016136
ENSGACG00000016767
ENSGACG00000017099
6207317
6207360
ENSGACG00000017172
7159351
ENSGACG00000017366
7533700
7638556
8356745
ENSGACG00000018013
ENSGACG00000018013
11968901
11968901
3,1
3,2
3,3
10,4
12,1
12,4
12,7
14,3
15,0
15,1
15,3
16,7
19,0
23,9

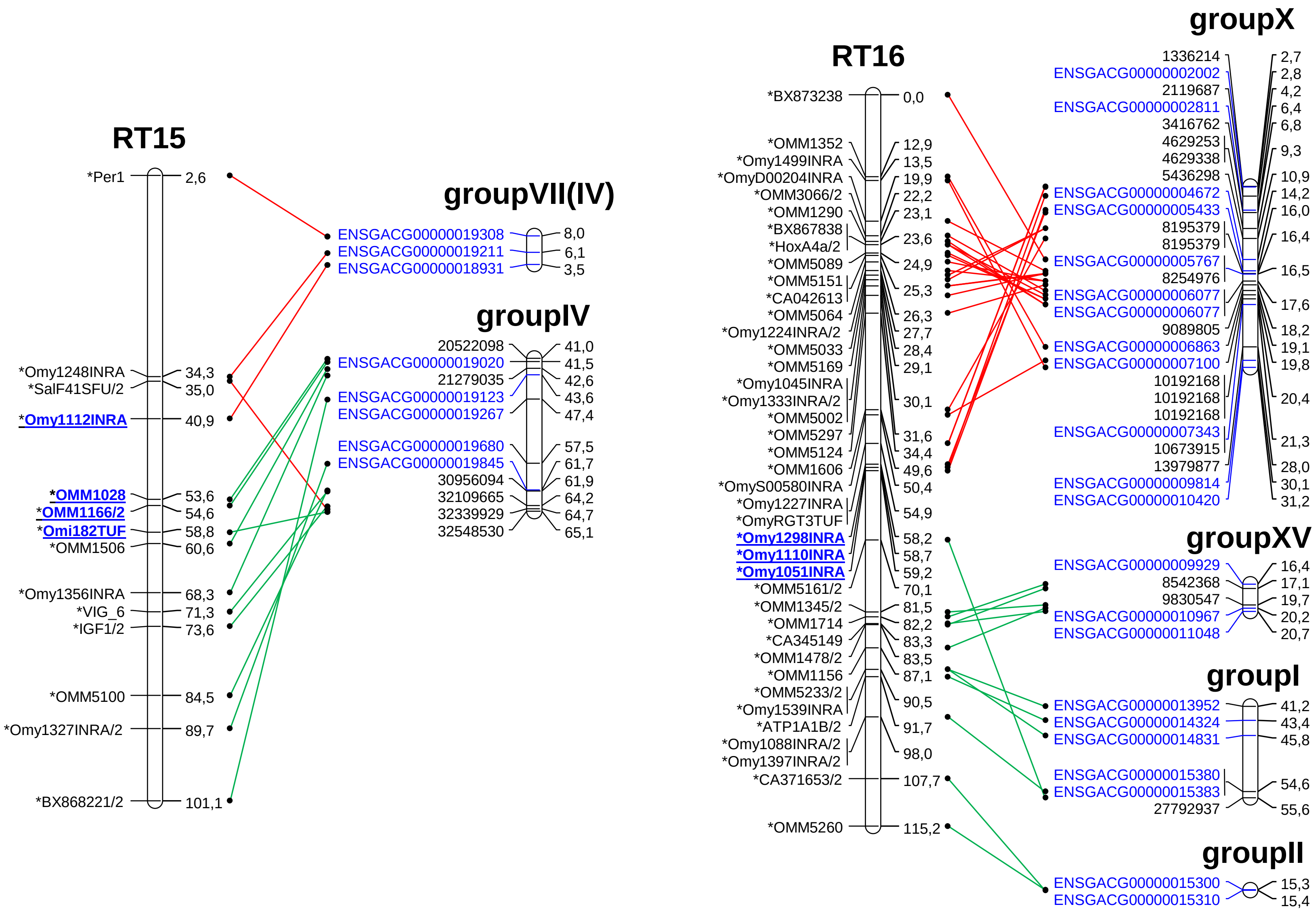
RT15
*Per1 2,6
*Omy1248INRA 34,3
*SalF41SFU/2 35,0
*Omy1112INRA 40,9
*OMM1028 53,6
*OMM1166/2 54,6
*Omi182TUF 58,8
*OMM1506 60,6
*Omy1356INRA 68,3
*VIG_6 71,3
*IGF1/2 73,6
*OMM5100 84,5
*Omy1327INRA/2 89,7
*BX868221/2 101,1
groupVII(IV)
ENSGACG00000019308 8,0
ENSGACG00000019211 6,1
ENSGACG00000018931 3,5
groupIV
20522098 41,0
ENSGACG00000019020 41,5
21279035 42,6
ENSGACG00000019123 43,6
ENSGACG00000019267 47,4
ENSGACG00000019680 57,5
ENSGACG00000019845 61,7
30956094 61,9
32109665 64,2
32339929 64,7
32548530 65,1
RT16
*BX873238 0,0
*OMM1352 12,9
*Omy1499INRA 13,5
*OmyD00204INRA 19,9
*OMM3066/2 22,2
*OMM1290 23,1
*BX867838 23,6
*HoxA4a/2 24,9
*OMM5089 25,3
*OMM5151 26,3
*CA042613 27,7
*OMM5064 28,4
*Omy1224INRA/2 29,1
*OMM5033 30,1
*OMM5169 31,6
*Omy1045INRA 34,4
*Omy1333INRA/2 49,6
*OMM5002 50,4
*OMM5297 54,9
*OMM5124 58,2
*OMM1606 58,7
*OmyS00580INRA 59,2
*Omy1227INRA 70,1
*OmyRGT3TUF
*Omy1298INRA
*Omy1110INRA
*Omy1051INRA
*OMM5161/2
*OMM1345/2 81,5
*OMM1714 82,2
*CA345149 83,3
*OMM1478/2 83,5
*OMM1156 87,1
*OMM5233/2 90,5
*Omy1539INRA 91,7
*ATP1A1B/2 98,0
*Omy1088INRA/2
*Omy1397INRA/2
*CA371653/2 107,7
*OMM5260 115,2
groupX
1336214 2,7
ENSGACG00000002002 2,8
2119687 4,2
ENSGACG00000002811 6,4
3416762 6,8
4629253 9,3
4629338 10,9
5436298 14,2
ENSGACG00000004672 16,0
ENSGACG00000005433 16,4
8195379 16,5
8195379 17,6
ENSGACG00000005767 18,2
8254976 19,1
ENSGACG00000006077 19,8
ENSGACG00000006077 20,4
9089805 21,3
ENSGACG00000006863 28,0
ENSGACG00000007100 30,1
10192168 31,2
10192168
10192168
ENSGACG00000007343
10673915
13979877
ENSGACG00000009814
ENSGACG00000010420
groupXV
ENSGACG00000009929 16,4
8542368 17,1
9830547 19,7
ENSGACG00000010967 20,2
ENSGACG00000011048 20,7
groupI
ENSGACG00000013952 41,2
ENSGACG00000014324 43,4
ENSGACG00000014831 45,8
ENSGACG00000015380 54,6
ENSGACG00000015383 55,6
27792937
groupII
ENSGACG00000015300 15,3
ENSGACG00000015310 15,4

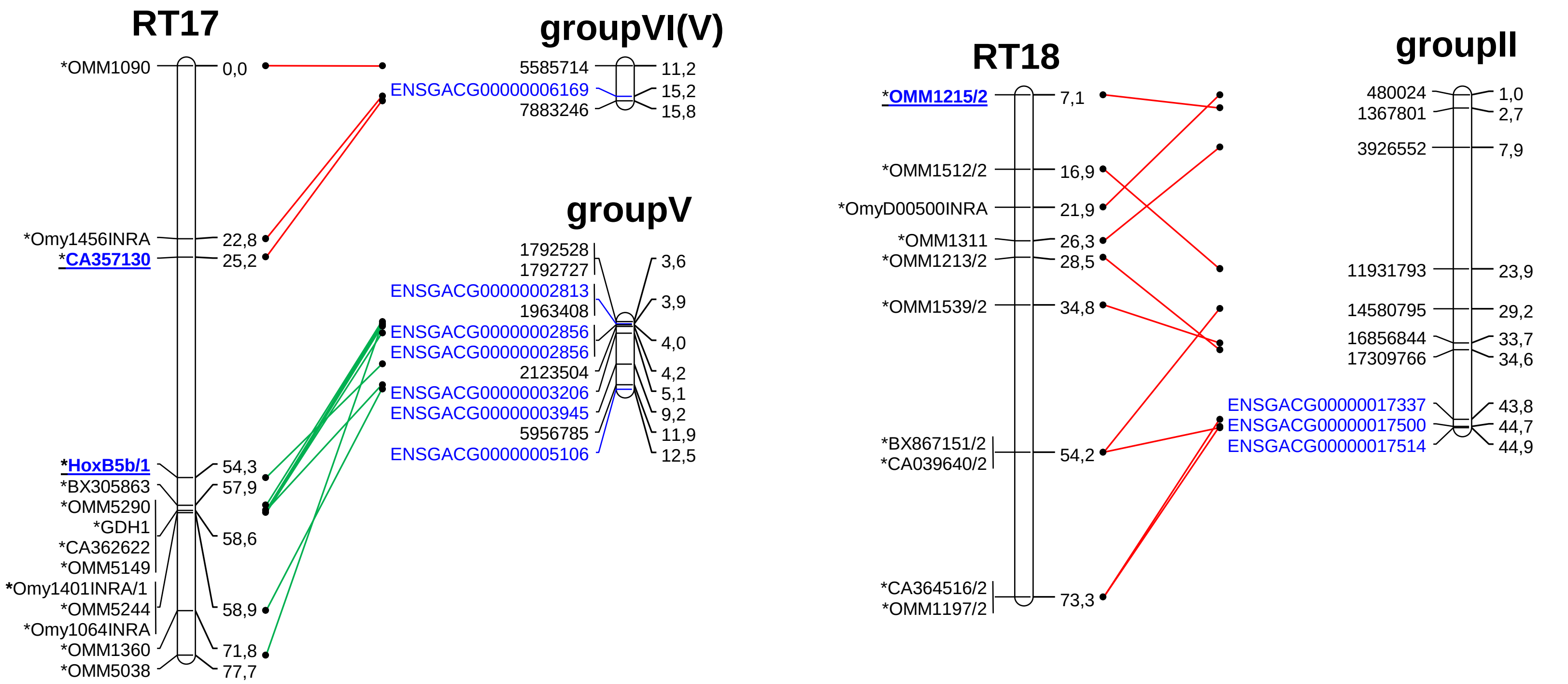
RT17
groupVI(V)
groupV
RT18
groupII
*OMM1090
0,0
*Omy1456INRA
22,8
*CA357130
25,2
*HoxB5b/1
54,3
*BX305863
57,9
*OMM5290
*GDH1
*CA362622
58,6
*OMM5149
*Omy1401INRA/1
*OMM5244
58,9
*Omy1064INRA
*OMM1360
71,8
*OMM5038
77,7
5585714
11,2
ENSGACG00000006169
15,2
7883246
15,8
1792528
1792727
3,6
ENSGACG00000002813
1963408
3,9
ENSGACG00000002856
ENSGACG00000002856
4,0
2123504
4,2
ENSGACG00000003206
5,1
ENSGACG00000003945
9,2
5956785
11,9
ENSGACG00000005106
12,5
*OMM1215/2
7,1
*OMM1512/2
16,9
*OmyD00500INRA
21,9
*OMM1311
26,3
*OMM1213/2
28,5
*OMM1539/2
34,8
*BX867151/2
*CA039640/2
54,2
*CA364516/2
*OMM1197/2
73,3
480024
1,0
1367801
2,7
3926552
7,9
11931793
23,9
14580795
29,2
16856844
33,7
17309766
34,6
ENSGACG00000017337
43,8
ENSGACG00000017500
44,7
ENSGACG00000017514
44,9

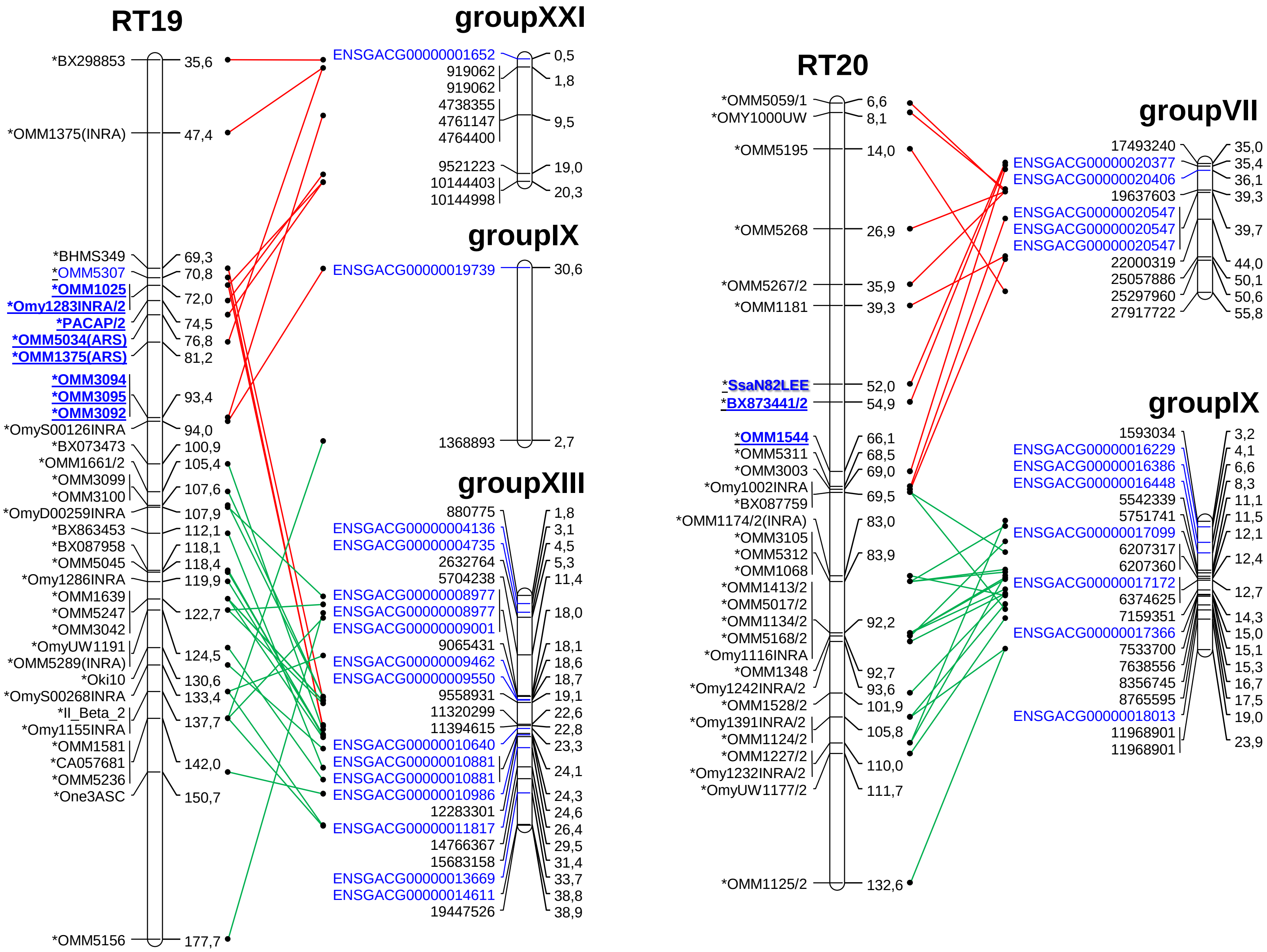
RT19
groupXXI
groupIX
groupXIII
RT20
groupVII
groupIX
*BX298853
35,6
*OMM1375(INRA)
47,4
*BHMS349
*OMM5307
*OMM1025
*Omy1283INRA/2
*PACAP/2
*OMM5034(ARS)
*OMM1375(ARS)
*OMM3094
*OMM3095
*OMM3092
*OmyS00126INRA
*BX073473
*OMM1661/2
*OMM3099
*OMM3100
*OmyD00259INRA
*BX863453
*BX087958
*OMM5045
*Omy1286INRA
*OMM1639
*OMM5247
*OMM3042
*OmyUW1191
*OMM5289(INRA)
*Oki10
*OmyS00268INRA
*II_Beta_2
*Omy1155INRA
*OMM1581
*CA057681
*OMM5236
*One3ASC
*OMM5156
ENSGACG00000001652
ENSGACG00000019739
ENSGACG00000004136
ENSGACG00000004735
ENSGACG00000008977
ENSGACG00000009001
ENSGACG00000009462
ENSGACG00000009550
ENSGACG00000010640
ENSGACG00000010881
ENSGACG00000010986
ENSGACG00000011817
ENSGACG00000013669
ENSGACG00000014611
*OMM5059/1
*OMY1000UW
*OMM5195
*OMM5268
*OMM5267/2
*OMM1181
*SsaN82LEE
*BX873441/2
*OMM1544
*OMM5311
*OMM3003
*Omy1002INRA
*BX087759
*OMM1174/2(INRA)
*OMM3105
*OMM5312
*OMM1068
*OMM1413/2
*OMM5017/2
*OMM1134/2
*OMM5168/2
*Omy1116INRA
*OMM1348
*Omy1242INRA/2
*OMM1528/2
*Omy1391INRA/2
*OMM1124/2
*OMM1227/2
*Omy1232INRA/2
*OmyUW1177/2
*OMM1125/2
ENSGACG00000020377
ENSGACG00000020406
ENSGACG00000020547
ENSGACG00000016229
ENSGACG00000016386
ENSGACG00000016448
ENSGACG00000017099
ENSGACG00000017172
ENSGACG00000017366
ENSGACG00000018013

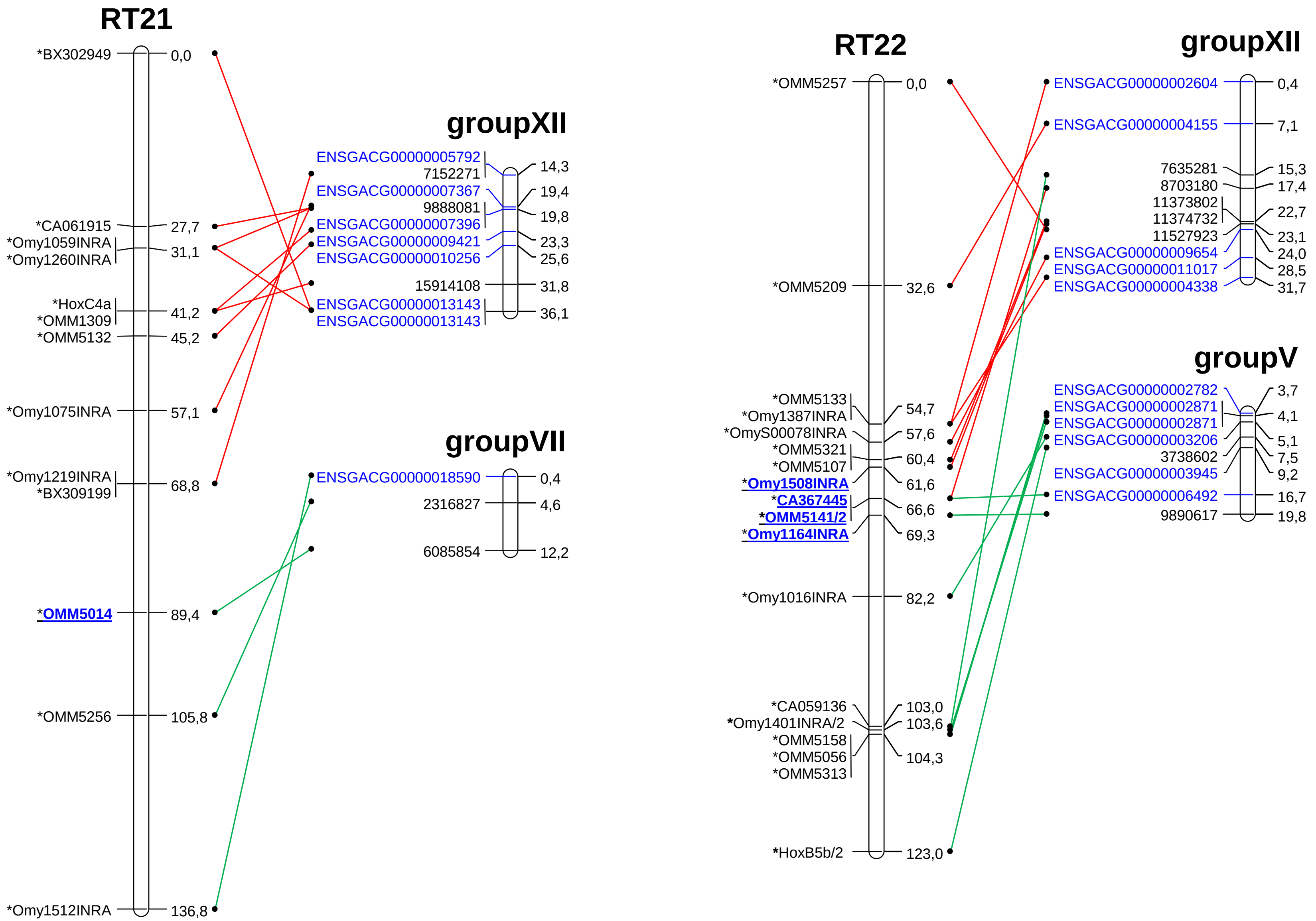
RT21
*BX302949 0,0
*CA061915 27,7
*Omy1059INRA
*Omy1260INRA 31,1
*HoxC4a
*OMM1309 41,2
*OMM5132 45,2
*Omy1075INRA 57,1
*Omy1219INRA
*BX309199 68,8
*OMM5014 89,4
*OMM5256 105,8
*Omy1512INRA 136,8
groupXII
ENSGACG00000005792
7152271 14,3
ENSGACG00000007367 19,4
9888081 19,8
ENSGACG00000007396
ENSGACG00000009421 23,3
ENSGACG00000010256 25,6
15914108 31,8
ENSGACG00000013143
ENSGACG00000013143 36,1
groupVII
ENSGACG00000018590 0,4
2316827 4,6
6085854 12,2
RT22
*OMM5257 0,0
*OMM5209 32,6
*OMM5133 54,7
*Omy1387INRA 57,6
*OmyS00078INRA 60,4
*OMM5321 61,6
*OMM5107 66,6
*Omy1508INRA 69,3
*CA367445
*OMM5141/2
*Omy1164INRA
*Omy1016INRA 82,2
*CA059136 103,0
*Omy1401INRA/2 103,6
*OMM5158 104,3
*OMM5056
*OMM5313
*HoxB5b/2 123,0
groupXII
ENSGACG00000002604 0,4
ENSGACG00000004155 7,1
7635281 15,3
8703180 17,4
11373802 22,7
11374732 23,1
11527923 24,0
ENSGACG00000009654 28,5
ENSGACG00000011017 31,7
ENSGACG00000004338
groupV
ENSGACG00000002782 3,7
ENSGACG00000002871 4,1
ENSGACG00000002871 5,1
ENSGACG00000003206 7,5
3738602 9,2
ENSGACG00000003945
ENSGACG00000006492 16,7
9890617 19,8

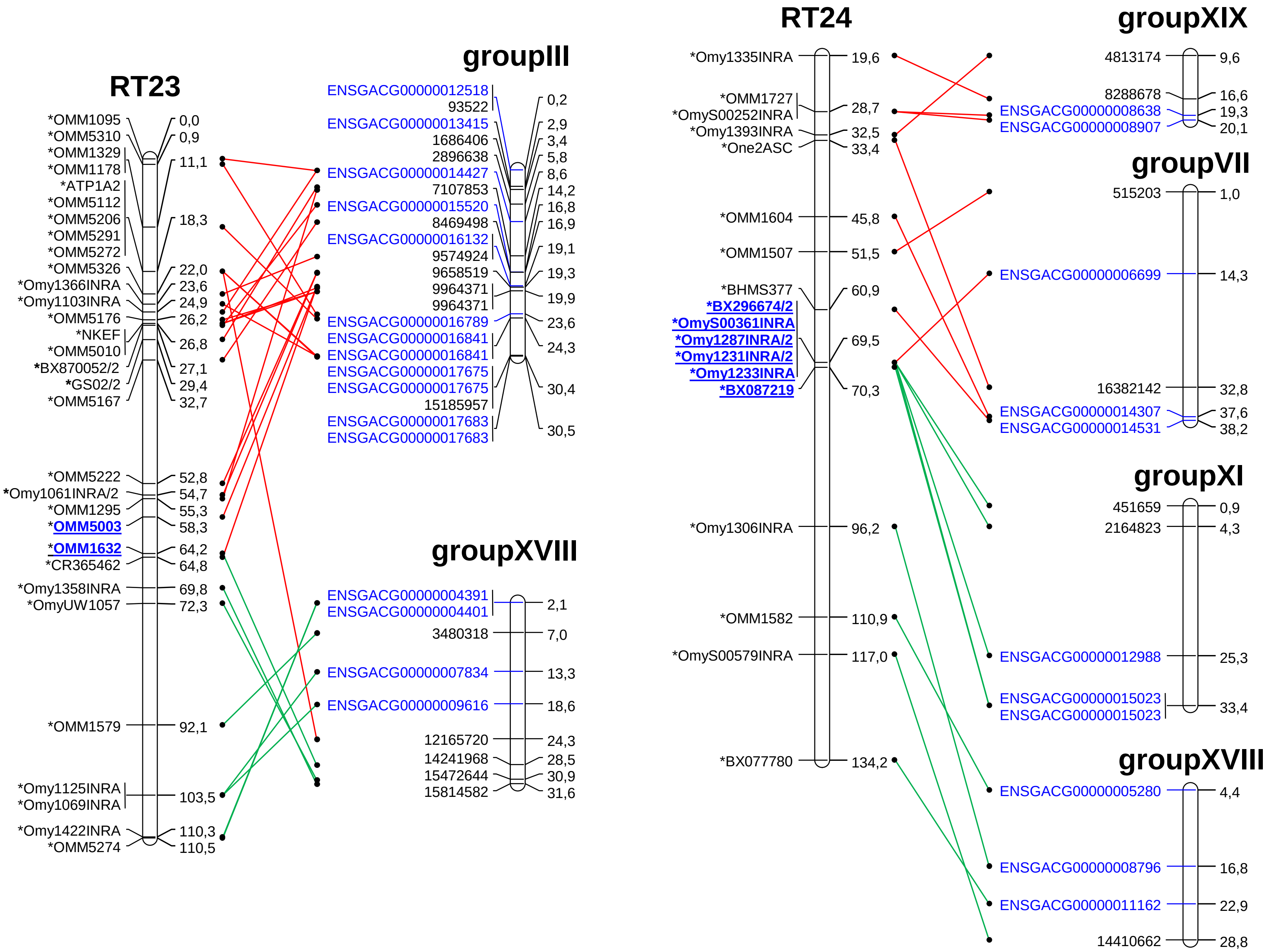

RT23
groupIII
groupXVIII
RT24
groupXIX
groupVII
groupXI
groupXVIII
*OMM1095 0,0
*OMM5310 0,9
*OMM1329
*OMM1178 11,1
*ATP1A2
*OMM5112
*OMM5206 18,3
*OMM5291
*OMM5272
*OMM5326 22,0
*Omy1366INRA 23,6
*Omy1103INRA 24,9
*OMM5176 26,2
*NKEF 26,8
*OMM5010 27,1
*BX870052/2 29,4
*GS02/2 32,7
*OMM5167
*OMM5222 52,8
*Omy1061INRA/2 54,7
*OMM1295 55,3
*OMM5003 58,3
*OMM1632 64,2
*CR365462 64,8
*Omy1358INRA 69,8
*OmyUW1057 72,3
*OMM1579 92,1
*Omy1125INRA 103,5
*Omy1069INRA
*Omy1422INRA 110,3
*OMM5274 110,5
ENSGACG00000012518 0,2
93522
ENSGACG00000013415 2,9
1686406 3,4
2896638 5,8
ENSGACG00000014427 8,6
7107853 14,2
ENSGACG00000015520 16,8
8469498 16,9
ENSGACG00000016132 19,1
9574924
9658519 19,3
9964371 19,9
9964371
ENSGACG00000016789 23,6
ENSGACG00000016841 24,3
ENSGACG00000016841
ENSGACG00000017675 30,4
ENSGACG00000017675
15185957
ENSGACG00000017683 30,5
ENSGACG00000017683
ENSGACG00000004391 2,1
ENSGACG00000004401
3480318 7,0
ENSGACG00000007834 13,3
ENSGACG00000009616 18,6
12165720 24,3
14241968 28,5
15472644 30,9
15814582 31,6
*Omy1335INRA 19,6
*OMM1727 28,7
*OmyS00252INRA
*Omy1393INRA 32,5
*One2ASC 33,4
*OMM1604 45,8
*OMM1507 51,5
*BHMS377 60,9
*BX296674/2
*OmyS00361INRA 69,5
*Omy1287INRA/2
*Omy1231INRA/2
*Omy1233INRA 70,3
*BX087219
*Omy1306INRA 96,2
*OMM1582 110,9
*OmyS00579INRA 117,0
*BX077780 134,2
4813174 9,6
8288678 16,6
ENSGACG00000008638 19,3
ENSGACG00000008907 20,1
515203 1,0
ENSGACG00000006699 14,3
16382142 32,8
ENSGACG00000014307 37,6
ENSGACG00000014531 38,2
451659 0,9
2164823 4,3
ENSGACG00000012988 25,3
ENSGACG00000015023 33,4
ENSGACG00000015023
ENSGACG00000005280 4,4
ENSGACG00000008796 16,8
ENSGACG00000011162 22,9
14410662 28,8

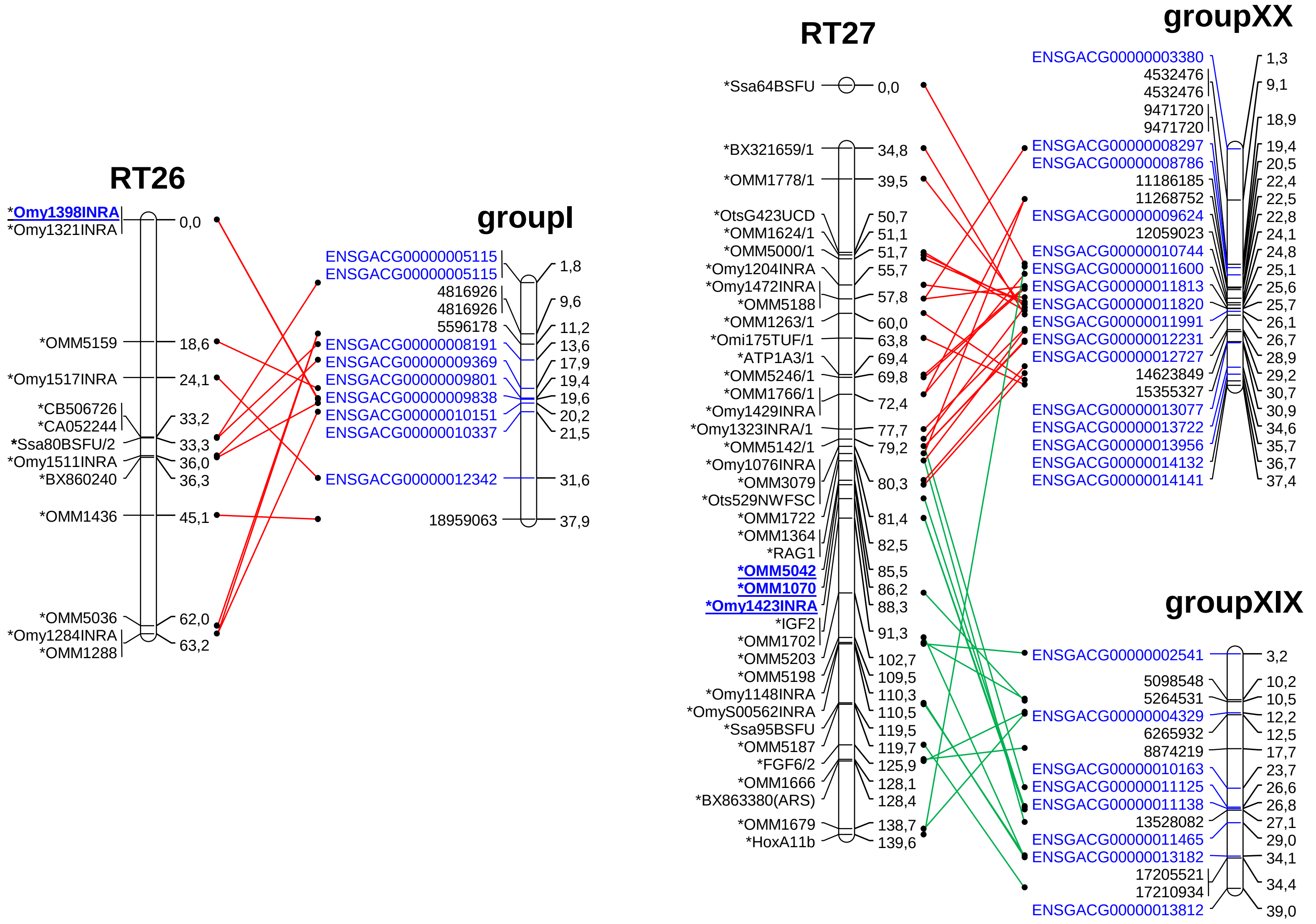
RT26
*Omy1398INRA
*Omy1321INRA
0,0
*OMM5159
18,6
*Omy1517INRA
24,1
*CB506726
*CA052244
*Ssa80BSFU/2
*Omy1511INRA
*BX860240
33,2
33,3
36,0
36,3
*OMM1436
45,1
*OMM5036
*Omy1284INRA
*OMM1288
62,0
63,2
groupI
ENSGACG00000005115
ENSGACG00000005115
4816926
4816926
5596178
ENSGACG00000008191
ENSGACG00000009369
ENSGACG00000009801
ENSGACG00000009838
ENSGACG00000010151
ENSGACG00000010337
ENSGACG00000012342
18959063
1,8
9,6
11,2
13,6
17,9
19,4
19,6
20,2
21,5
31,6
37,9
RT27
*Ssa64BSFU
0,0
*BX321659/1
34,8
*OMM1778/1
39,5
*OtsG423UCD
*OMM1624/1
*OMM5000/1
*Omy1204INRA
*Omy1472INRA
*OMM5188
*OMM1263/1
*Omi175TUF/1
*ATP1A3/1
*OMM5246/1
*OMM1766/1
*Omy1429INRA
*Omy1323INRA/1
*OMM5142/1
*Omy1076INRA
*OMM3079
*Ots529NWFSC
*OMM1722
*OMM1364
*RAG1
*OMM5042
*OMM1070
*Omy1423INRA
*IGF2
*OMM1702
*OMM5203
*OMM5198
*Omy1148INRA
*OmyS00562INRA
*Ssa95BSFU
*OMM5187
*FGF6/2
*OMM1666
*BX863380(ARS)
*OMM1679
*HoxA11b
50,7
51,1
51,7
55,7
57,8
60,0
63,8
69,4
69,8
72,4
77,7
79,2
80,3
81,4
82,5
85,5
86,2
88,3
91,3
102,7
109,5
110,3
110,5
119,5
119,7
125,9
128,1
128,4
138,7
139,6
groupXX
ENSGACG00000003380
4532476
4532476
9471720
9471720
ENSGACG00000008297
ENSGACG00000008786
11186185
11268752
ENSGACG00000009624
12059023
ENSGACG00000010744
ENSGACG00000011600
ENSGACG00000011813
ENSGACG00000011820
ENSGACG00000011991
ENSGACG00000012231
ENSGACG00000012727
14623849
15355327
ENSGACG00000013077
ENSGACG00000013722
ENSGACG00000013956
ENSGACG00000014132
ENSGACG00000014141
1,3
9,1
18,9
19,4
20,5
22,4
22,5
22,8
24,1
24,8
25,1
25,6
25,7
26,1
26,7
28,9
29,2
30,7
30,9
34,6
35,7
36,7
37,4
groupXIX
ENSGACG00000002541
5098548
5264531
ENSGACG00000004329
6265932
8874219
ENSGACG00000010163
ENSGACG00000011125
ENSGACG00000011138
13528082
ENSGACG00000011465
ENSGACG00000013182
17205521
17210934
ENSGACG00000013812
3,2
10,2
10,5
12,2
12,5
17,7
23,7
26,6
26,8
27,1
29,0
34,1
34,4
39,0

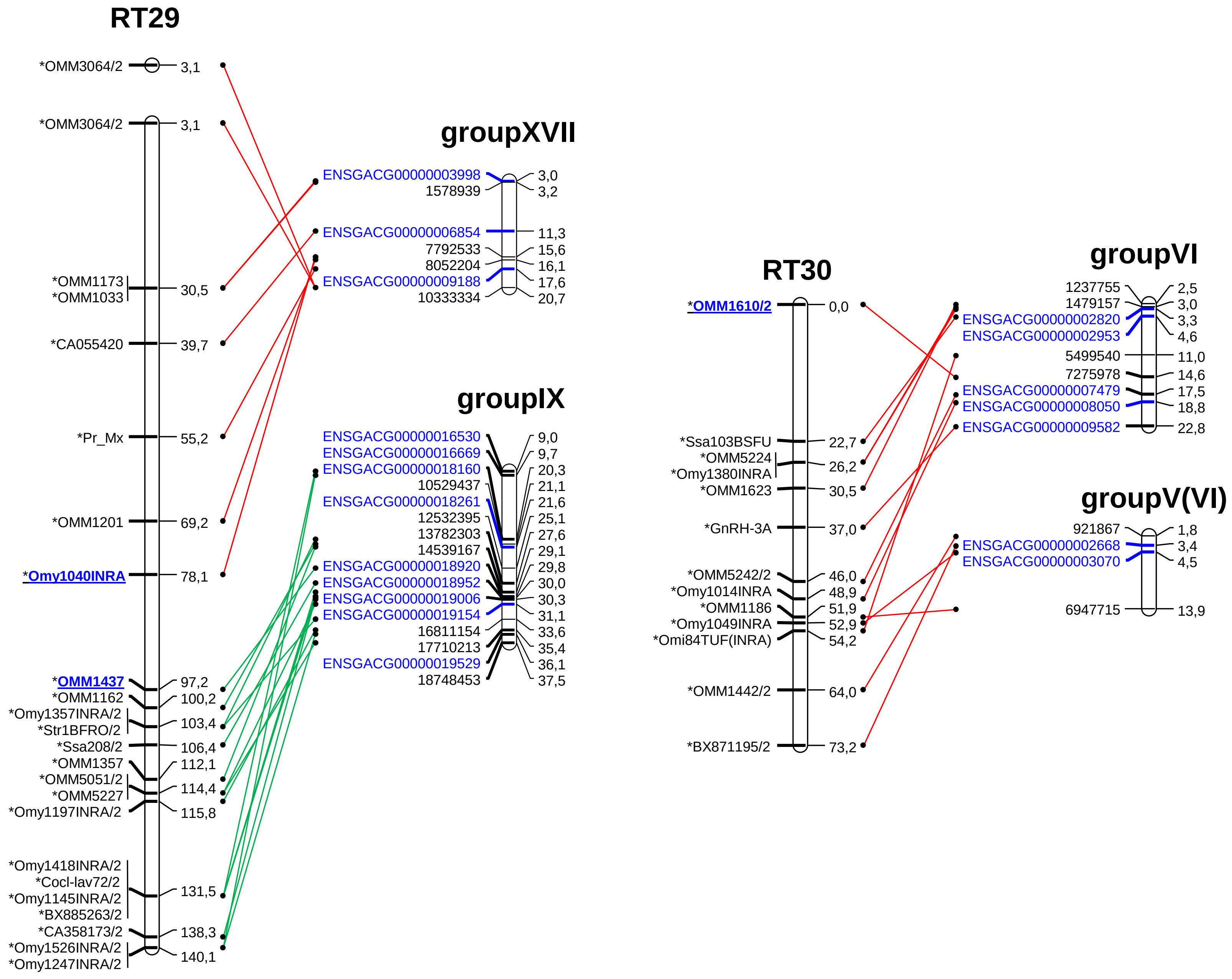

RT29
*OMM3064/2
3,1
*OMM3064/2
3,1
*OMM1173
*OMM1033
30,5
*CA055420
39,7
*Pr_Mx
55,2
*OMM1201
69,2
*Omy1040INRA
78,1
*OMM1437
97,2
*OMM1162
100,2
*Omy1357INRA/2
*Str1BFRO/2
103,4
*Ssa208/2
106,4
*OMM1357
112,1
*OMM5051/2
*OMM5227
114,4
*Omy1197INRA/2
115,8
*Omy1418INRA/2
*Cocl-lav72/2
*Omy1145INRA/2
*BX885263/2
131,5
*CA358173/2
138,3
*Omy1526INRA/2
*Omy1247INRA/2
140,1
groupXVII
ENSGACG00000003998
1578939
ENSGACG00000006854
7792533
8052204
ENSGACG00000009188
10333334
3,0
3,2
11,3
15,6
16,1
17,6
20,7
groupIX
ENSGACG00000016530
ENSGACG00000016669
ENSGACG00000018160
10529437
ENSGACG00000018261
12532395
13782303
14539167
ENSGACG00000018920
ENSGACG00000018952
ENSGACG00000019006
ENSGACG00000019154
16811154
17710213
ENSGACG00000019529
18748453
9,0
9,7
20,3
21,1
21,6
25,1
27,6
29,1
29,8
30,0
30,3
31,1
33,6
35,4
36,1
37,5
RT30
*OMM1610/2
0,0
*Ssa103BSFU
22,7
*OMM5224
*Omy1380INRA
26,2
*OMM1623
30,5
*GnRH-3A
37,0
*OMM5242/2
46,0
*Omy1014INRA
48,9
*OMM1186
51,9
*Omy1049INRA
52,9
*Omi84TUF(INRA)
54,2
*OMM1442/2
64,0
*BX871195/2
73,2
groupVI
1237755
1479157
ENSGACG00000002820
ENSGACG00000002953
5499540
7275978
ENSGACG00000007479
ENSGACG00000008050
ENSGACG00000009582
2,5
3,0
3,3
4,6
11,0
14,6
17,5
18,8
22,8
groupV(VI)
921867
ENSGACG00000002668
ENSGACG00000003070
6947715
1,8
3,4
4,5
13,9

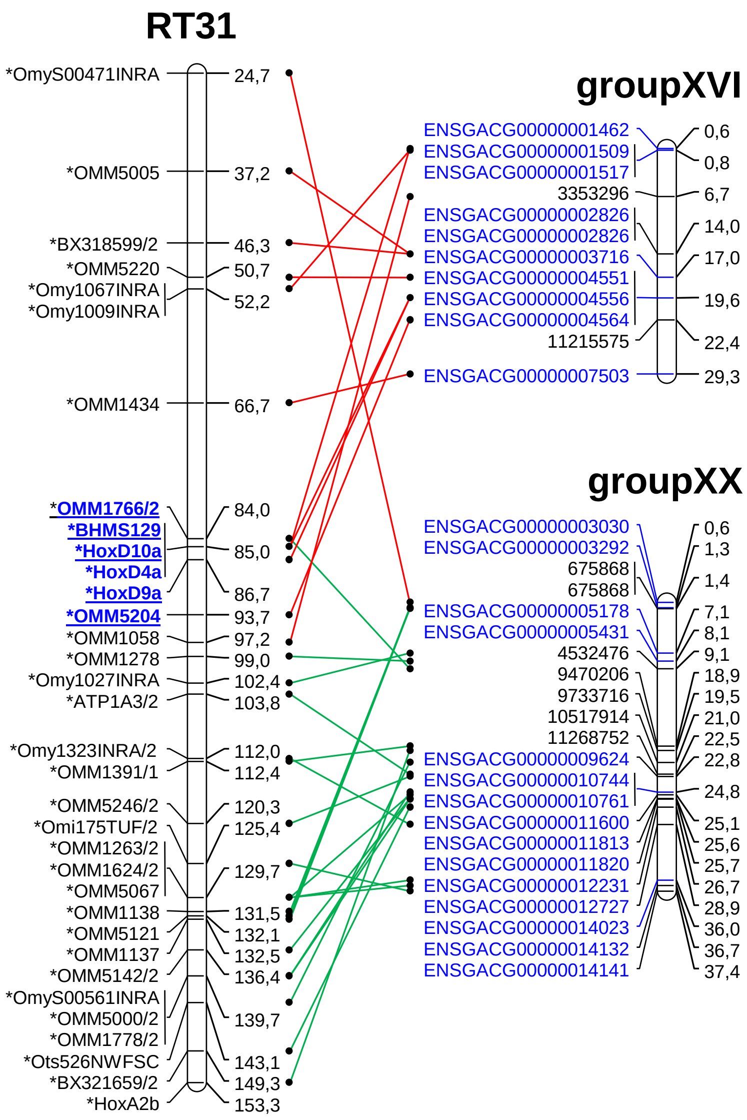
RT31
groupXVI
groupXX
*OmyS00471INRA
24,7
*OMM5005
37,2
*BX318599/2
46,3
*OMM5220
50,7
*Omy1067INRA
*Omy1009INRA
52,2
*OMM1434
66,7
*OMM1766/2
*BHMS129
*HoxD10a
*HoxD4a
*HoxD9a
84,0
85,0
86,7
*OMM5204
93,7
*OMM1058
97,2
*OMM1278
99,0
*Omy1027INRA
102,4
*ATP1A3/2
103,8
*Omy1323INRA/2
112,0
*OMM1391/1
112,4
*OMM5246/2
120,3
*Omi175TUF/2
125,4
*OMM1263/2
*OMM1624/2
129,7
*OMM5067
*OMM1138
131,5
*OMM5121
132,1
*OMM1137
132,5
*OMM5142/2
136,4
*OmyS00561INRA
*OMM5000/2
*OMM1778/2
139,7
*Ots526NWFSC
143,1
*BX321659/2
149,3
*HoxA2b
153,3
ENSGACG00000001462
ENSGACG00000001509
ENSGACG00000001517
3353296
ENSGACG00000002826
ENSGACG00000002826
ENSGACG00000003716
ENSGACG00000004551
ENSGACG00000004556
ENSGACG00000004564
11215575
ENSGACG00000007503
0,6
0,8
6,7
14,0
17,0
19,6
22,4
29,3
ENSGACG00000003030
ENSGACG00000003292
675868
675868
ENSGACG00000005178
ENSGACG00000005431
4532476
9470206
9733716
10517914
11268752
ENSGACG00000009624
ENSGACG00000010744
ENSGACG00000010761
ENSGACG00000011600
ENSGACG00000011813
ENSGACG00000011820
ENSGACG00000012231
ENSGACG00000012727
ENSGACG00000014023
ENSGACG00000014132
ENSGACG00000014141
0,6
1,3
1,4
7,1
8,1
9,1
18,9
19,5
21,0
22,5
22,8
24,8
25,1
25,6
25,7
26,7
28,9
36,0
36,7
37,4
